# Chemical synthesis of C6-tetrazole ᴅ-mannose building blocks and access to a bioisostere of mannuronic acid 1-phosphate

**DOI:** 10.3762/bjoc.17.110

**Published:** 2021-07-05

**Authors:** Eleni Dimitriou, Gavin J Miller

**Affiliations:** 1 Lennard-Jones Laboratory, School of Chemical and Physical Sciences, Keele University, Keele, Staffordshire, ST5 5BG, U. K.

**Keywords:** alginate, glycosyl 1-phosphate, non-native monosaccharide, tetrazole, uronate

## Abstract

Alginate is a biocompatible and industrially relevant polysaccharide that derives many of its important properties from the charged carboxylate groups within its polyuronic acid backbone. The design and inclusion of isosteric replacements for these carboxylates would underpin provision of new oligo-/polysaccharide materials with alternate physicochemical properties. Presented herein is our synthesis of mannuronic acid building blocks, appropriately modified at the carboxylate C6 position with a bioisosteric tetrazole. Thioglycosides containing a protected C6-tetrazole are accessed from a C6-nitrile, through dipolar cycloaddition using NaN_3_ with *n*-Bu_2_SnO. We also demonstrate access to orthogonally C4-protected donors, suitable for iterative oligosaccharide synthesis. The development of these building blocks is showcased to access anomeric 3-aminopropyl- and 1-phosphate free sugars containing this non-native motif.

## Introduction

**Figure 1:**
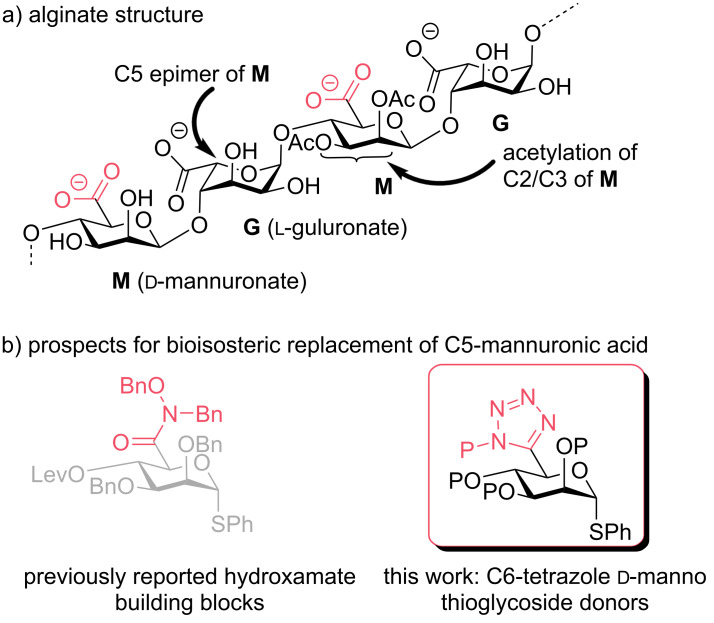
a) Chemical structure of alginate showing constituent **M** and **G** residues and C2/C3 acetylation for one **M** residue. b) Introduction of bioisosteric carboxylate groups at C6 within a ᴅ-manno thioglycoside donor, P = appropriate protecting group.

Alginate is an important, industrially relevant polysaccharide chemically composed of β-1,4-linked ᴅ-mannuronic acid (**M**) and α-ʟ-guluronic acid (**G**) monosaccharide building blocks (Figure 1a). Because of its biocompatibility, alginate has several commercial industrial applications; for example, it is widely used as a gelling agent [1–5]. Detailed consideration of the alginate sub-structure indicates non-uniform proportions of **M** and **G** units (and their homo- or heteropolymeric block-groupings) which, alongside acetylation of **M** residues, presents a structurally heterogeneous polysaccharide. Ultimately, this heterogeneity contributes to the physicochemical properties of the polysaccharide system, presenting an opportunity to explore modifying the chemical structure of alginate, with a view to both understanding and imparting changes upon its structure-to-function relationships.

To this end, synthetic chemistry strategies (both solution and solid phase) have demonstrated exciting capabilities for access to native alginate oligosaccharides in recent years [6–10]. As part of a program to access non-native alginate oligosaccharide sequences, we targeted a synthetic approach to provide structurally defined building blocks containing bioisosteres of ᴅ-mannuronic acid. Building on our recently reported synthesis and glycosylation capability of hydroxamate-modified ᴅ-mannuronate building blocks [11], we now demonstrate the synthesis of a second carboxylate C6-bioisostere, tetrazole (Figure 1b).

As an established bioisostere for a carboxylic acid, tetrazole has found significant application within medicinal chemistry [12]. The aromatic tetrazole ring is considered a non-classical bioisostere, differing in size and number of atoms to the carboxylic acid. The functional group has a similar p*K*_a_ to a carboxylate yet provides enhanced hydrogen bonding capability and alternative prospects for permeability due to a larger hydrophobic region enabling improved lipophilic contacts. These alternative properties and a prospect for their inclusion within new alginate fragments led us to explore the synthesis of a ᴅ-*manno* C6-tetrazole thioglycoside donor and examine subsequent installation of C1 phosphate and anomeric linker groups.

## Results and Discussion

**Scheme 1:**
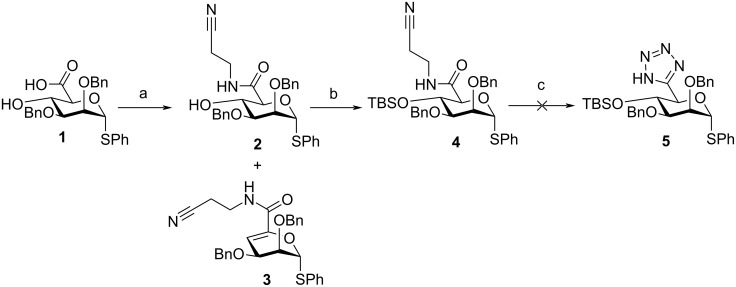
a) H_2_N(CH_2_)_2_CN, PyBOP, DIPEA, CH_2_Cl_2_, 0 °C, 40 min, 47% (+44% **3**); b) TBSOTf, imidazole, DMAP, DMF, 24 h, 80%; c) PPh_3_, DIAD, TMSN_3_, MeCN, 80 °C, 48 h.

An initial route towards a protected C6-tetrazole building block started from known mannuronic acid thioglycoside **1** (Scheme 1) [13], from which we recently effected coupling to yield a protected C6 hydroxamate [11]. Accordingly, **1** was stirred with PyBOP and DIPEA in CH_2_Cl_2_ for 5 minutes, before 3-aminopropionitrile was added. After stirring for 1.5 hours at room temperature an undesired C4–C5 elimination material **3** was isolated as the major product in 35% yield, with only a trace amount of the desired **2** formed (7% yield). The ability of 3-aminopropionitrile to act as a base and trigger this elimination was comparable to results we observed using *N*,*O*-dibenzylhydroxylamine as the coupling partner [11]. In the latter instance we were able to modify the nucleophile component to *O*-benzylhydroxylamine and supress unwanted elimination. Unable to do this here, we instead reduced the reaction temperature to 0 °C, maintaining this for 40 minutes. Pleasingly, the yield and product distribution were improved, affording separable amounts of **2** and **3** in 47% and 44% yields, respectively.

**Scheme 2:**
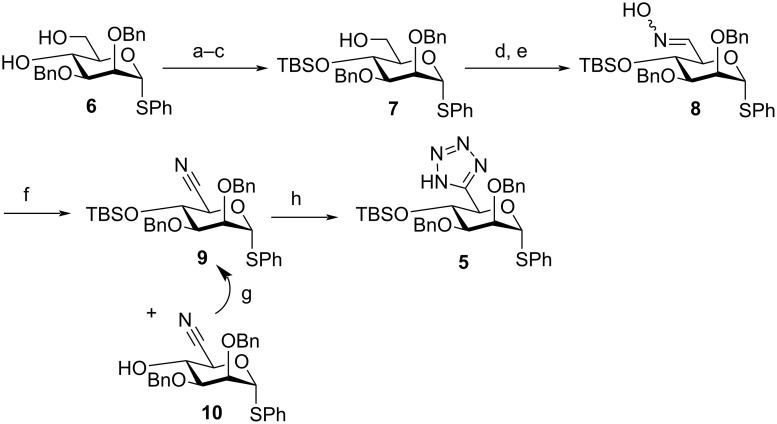
a) BzCl, DMAP, pyridine, CH_2_Cl_2_, rt, 24 h, 90%; b) TBSOTf, imidazole, DMAP, DMF, 40 °C, 24 h, 78%; c) Na(s), MeOH, THF, 16 h, 90%; d) DMSO, SO_3_·pyridine, Et_3_N, rt, 1 h, 98%; e) H_2_NOH·HCl, THF, H_2_O, Na_2_CO_3_, 24 h, 80%; f) POCl_3_, MeCN, 65 °C, 40%; g) TBSOTf, imidazole, DMAP, DMF, rt, 24 h, 87%; h) TMSN_3_, Bu_2_SnO, toluene, 120 °C, 51%.

Subsequent silyl protection of the C4-hydroxy group in **2** was completed using TBSOTf, furnishing **4** in 80% yield. Attempts to next convert **4** to **5** (via an *N*-cyanoethyl-protected tetrazole) using PPh_3_, DIAD and TMSN_3_ were unsuccessful, despite repeated attempts [14]. TLC and NMR analysis consistently indicated no conversion of **4**, even after stirring at 80 °C in MeCN for 48 hours. We therefore proposed an alternative route to **5**, directly from reaction of a C6 nitrile with NaN_3_, obviating the need for intermediate cyanoacetamide formation (Scheme 2).

This second route commenced with a three-step protecting group manipulation of primary alcohol **6**, delivering **7** in 63% yield over three steps (Scheme 2). Alcohol **7** was then subjected to Parikh–Doering oxidation to deliver a crude aldehyde in 98% yield, from which oxime **8** was subsequently formed in 80% yield as a 6.7:1 mixture of C=N isomers. Dehydration of **8** using POCl_3_ gave **9** in a low 40% yield, alongside **10** (26% yield). Formation of **10** was attributed to the acidic reaction conditions concomitantly effecting TBS removal. However, further amounts of **9** could be accessed through reprotection at C4 with TBSOTf in excellent yield (87%). C6-nitrile **9** was then successfully converted into C6-tetrazole **5** in 51% yield using TMSN_3_ and a catalytic amount of Bu_2_SnO [15]. This method was recently utilised successfully by Bräse and colleagues for ᴅ-gluco-configured C6-tetrazoles in their synthesis of modified hyaluronic acid fragments [16]. ^13^C NMR of **5** confirmed the presence of a new quaternary carbon (tetrazole C_q_, δ_C_ = 155.8 ppm) alongside disappearance of the C6-nitrile (δ_C_ = 117.0 ppm). Furthermore, ^1^H NMR analysis indicated the common H5 doublet was further downfield (δ_H_ = 5.64 ppm), compared to data observed previously for mannuronate ester (δ_H_ = 4.54 ppm) and hydroxamate (δ_H_ = 4.56 ppm) motifs [11]. Finally, the coupling constant calculated for H5 (^3^*J*_H5-H4_ = 8.9 Hz), indicated a solution-phase ^4^*C*_1_ pyranose conformation for newly formed **5**.

To explore improving the efficiency of the latter synthetic steps towards **5**, an alternative, one-pot three-component procedure (H_2_N-OH, NaN_3_ and catalytic [(NH_4_)_4_Ce(SO_4_)_4_]) was attempted from the crude C6-aldehyde [17]. TLC analysis indicated C6-nitrile formation was evident after 36 h, however, the desired C6-tetrazole **5** was not observed. Repeated attempts were unable to indicate progress beyond mixtures of **8** and **9** in 35% and 14% yields and the procedure was abandoned, instead reverting to the successful route developed in Scheme 2.

**Scheme 3:**
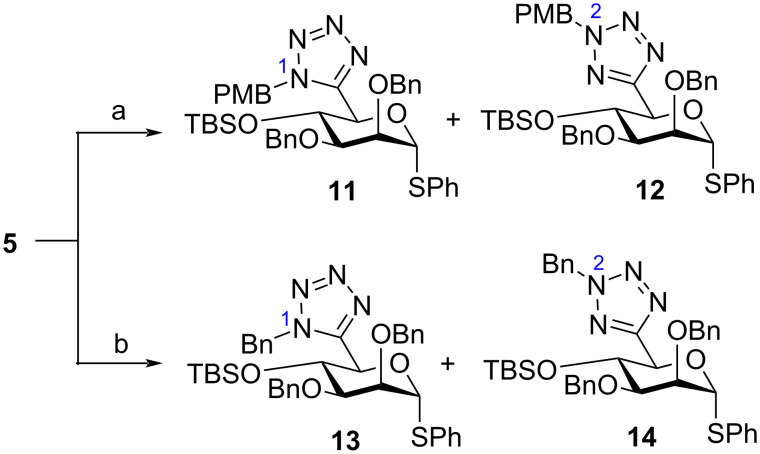
a) PMBCl, KI, K_2_CO_3_, DMF, rt, 53% for **11** and **12**; b) BnBr, DMF, Et_3_N, DCM, rt, 31% for **13** and **14**.

The final step towards the synthesis of a fully protected C6-tetrazole glycosyl donor required tetrazole nitrogen protection (Scheme 3). A first attempt here involved the reaction of **5** with PMBCl in DMF, using K_2_CO_3_ alongside KI. Two separable regioisomers **11** and **12** were isolated in an acceptable 53% overall yield and in a ratio of *N*_1_-PMB/*N*_2_-PMB = 1.1:1 (Scheme 3). HMBC NMR of **11** and **12** clarified the position of the PMB group on the tetrazole ring for each compound. For **11**, a correlation of tetrazole C_q_ (δ_C_ = 150.4 ppm) with the benzylic protons of the PMB group (δ_H_ = 5.66 ppm, see Supporting Information File 1, Figure S3), was observable. Similar analysis for **12** indicated no such correlation. In order to try and improve the *N*_1_-PMB/*N*_2_-PMB ratio, converting **5** to a triethylammonium salt form in 94% yield was adopted [18]. Subsequent reaction with PMBCl gave **11** and **12**, but in a largely unchanged ratio (*N*_1_-PMB/*N*_2_-PMB = 1:1.1). A comparative attempt to install a benzyl protecting group using this method afforded *N*_1_-Bn and *N*_2_-Bn tetrazoles **13** and **14** in low yield (31%) and again with little regiodiscrimination (*N*_1_-Bn/*N*_2_-Bn = 1:1.2).

The synthesis of appropriately protected C6-tetrazole donors **11**–**14** was accomplished to allow for regioselective deprotection and unveil C4-acceptor capability within a broader oligosaccharide synthesis strategy. With such capability effectively demonstrated, we next explored the provision of ᴅ-manno C6-tetrazoles without an orthogonal C4-protecting group.

**Scheme 4:**
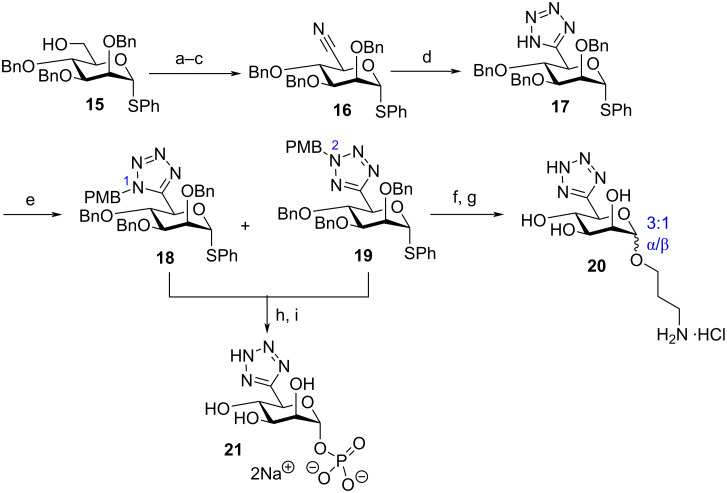
a) DMSO, SO_3_·pyridine, Et_3_N, rt, 1 h, 96%; b) H_2_NOH·HCl, THF, H_2_O, Na_2_CO_3_, 89%; c) POCl_3_, MeCN, 65 °C, 59%; d) TMSN_3_, Bu_2_SnO, toluene, 120 °C, 55%; e) PMBCl, KI, K_2_CO_3_, DMF, 76% for **18** and **19**; f) 3-(benzyloxycarbonylamino)-propan-1-ol, NIS, AgOTf, CH_2_Cl_2_, −40 to −10 °C, 3 h, 34%; g) H_2_(g), Pd/C, Pd(OH)_2_/C, HCl, EtOH, THF, rt, 56 h, 96%; h) dibenzyl phosphate, NIS, AgOTf, CH_2_Cl_2_, −30 to 0 °C, 3.5 h, 72%; i) H_2_(g), Pd/C, Pd(OH)_2_/C, 5% NaHCO_3_, EtOH, THF, rt, 24 h, 72%.

Accordingly, a synthesis initiating from alcohol **15** [19] enabled access to C6-nitrile **16** in three steps (Scheme 4) and an improved yield of 50% (compared to 31% in accessing **9** from **7**). Nitrile **16** then underwent dipolar cycloaddition with NaN_3_, converting it to C6-tetrazole thioglycoside **17** in 55% yield. This material was then protected at tetrazole nitrogen in 76% yield using PMBCl to give **18** and **19** (*N*_1_-PMB/*N*_2_-PMB = 1:1.2) as separable regioisomers and their structures were confirmed by HMBC, as previously demonstrated. Removal of the need to orthogonally protect C4 expectedly reduced the complexity of the synthetic route and nine steps for the synthesis of donors of type **11**/**12** was reduced to five steps for **18**/**19**. Moreover, the overall yield for the route increased from 9 to 21%.

To demonstrate capability for anomeric linker attachment and conversion to a biologically relevant analogue of mannuronic acid 1-phosphate, 3-aminopropyl glycoside **20** and glycosyl 1-phosphate **21** were synthesised (Scheme 4). The mixture **18**/**19** was used for glycosylation of 3-(benzyloxycarbonylamino)-1-propanol and furnished a regioisomeric and anomeric mixture in low yield (34%, with 20% recovered starting material and 18% hydrolysed donor, 3:1, α/β). The β-linked (minor) anomer was identified through ^1^*J*_C-H_ coupling constant data (*J* = 156 Hz), similar to products obtained using C6-mannuronate and C6-hydroxamate donors [11]. This initial result suggests a reduced capability using C6-tetrazole donors with a primary alcohol acceptor; comparative yields for glycosylation of 3-bromopropanol using C6-hydroxamate and C6-mannuronate donors were 65–85% and exclusively β-selective [11]. Isomeric separation of this complex mixture was not completed at this stage and the material was next deprotected using hydrogenolysis to remove the benzyl groups. This furnished **20** in excellent yield (96%) as a 3:1 α/β mixture (δ 100.5 ppm [^1^*J*_C1-H1_ = 172 Hz, for the α-anomer]).

Additionally, glycosylation of dibenzyl phosphate using the mixture **18**/**19** was successful and furnished the expected mixture of tetrazole *N*-regioisomers in 72% yield. These materials were not separated and instead exposed to hydrogenolysis conditions to deliver free ᴅ-manno C6-tetrazole 1-phosphate **21**. Deprotection utilised 0.6 equiv of Pd/C (0.1 equiv per benzyl group) and 0.6 equiv of Pd(OH)_2_/C to afford **21** in 72% yield after 24 h. NMR analysis of **21** confirmed the presence of a C6-tetrazole (δ_C_ = 160.8 ppm), alongside an anomeric phosphate (δ_P_ = −2.15 ppm), and ^1^H coupling constant data (δ 5.41 ppm [dd, *J* = 7.9, 1.7 Hz, H_1_]) supported an α-linked *manno* 1-phosphate derivative. This material complimented our recently reported C6-hydroxamic acid derivative as a bioisostere for mannuronic acid 1-phosphate [20], and will be enabling for evaluating non-native glycosyl 1-phosphates in appropriate chemoenzymatic syntheses [21–23].

## Conclusion

We have established synthetic access to a series of C6-tetrazole thioglycoside monosaccharide building blocks with capability for orthogonal C4- and tetrazole *N*-protecting groups. We demonstrate anomeric manipulation of these donors to new, biologically relevant 1-phosphate and conjugable, aminopropyl-tethered materials as mimics of mannuronic acid. Evaluation of these C6-tetrazole thioglycosides as donors for non-native alginate fragment synthesis is currently underway and will be reported in due course.

## Supporting Information

File 1 Detailed experimental protocols and characterisation data; spectral NMR data (^1^H, ^13^C, ^31^P and HSQC NMR for compounds **2**–**5**, **7**–**14**, **16**–**18**, **20** and **21**).
