# Supplementary material for: Chemical synthesis of C6-tetrazole ᴅ-mannose building blocks and access to a bioisostere of mannuronic acid 1-phosphate
Source: Beilstein J Org Chem. 2021 Jul 5;17:1527–32. doi: 10.3762/bjoc.17.110 (PMC8275867; doi:10.3762/bjoc.17.110)

BEILSTEIN JOURNAL OF ORGANIC CHEMISTRY

Supporting Information

for

Chemical synthesis of C6-tetrazole D-mannose building blocks and access to a bioisostere of mannuronic acid 1-phosphate

Eleni Dimitriou and Gavin J. Miller

Beilstein J. Org. Chem. **2021,** *17,* 1527–1532. doi:10.3762/bjoc.17.110

Detailed experimental protocols and characterisation data; spectral NMR data (^{1}H, ^{13}C, ^{31}P and HSQC NMR for compounds 2–5, 7–14, 16–18, 20 and 21)

License and Terms: This is a supporting information file under the terms of the Creative Commons Attribution License (https://creativecommons.org/licenses/by/4.0). Please note that the reuse, redistribution and reproduction in particular requires that the author(s) and source are credited and that individual graphics may be subject to special legal provisions.
The license is subject to the *Beilstein Journal of Organic Chemistry* terms and conditions: (https://www.beilstein-journals.org/bjoc/terms)

The following pages contain representative supporting information and data.

S1. General experimental

S2. Experimental procedures for compounds 2–21

S3. HMBC spectrum for N1-protected tetrazole 11

S4. References

S5. Spectral Data: ^{1}H, ^{13}C, ^{31}P and HSQC NMR for compounds 2–5, 7–14, 16–18, 20 and 21

S1. General experimental

All reagents and solvents which were available commercially were purchased from Acros, Alfa Aesar, Fisher Scientific, Sigma Aldrich or TCI. All reactions in non-aqueous solvents were conducted in oven dried glassware with a magnetic stirring device under an inert atmosphere of nitrogen passed through a drying column using a vacuum manifold. Solvents were purified by passing through activated alumina columns and used directly from a Pure Solv-MD solvent purification system and were transferred under nitrogen. Reactions were followed by thin layer chromatography (TLC) using Merck silica gel 60F254 analytical plates (aluminium support) and were developed using short wave UV radiation (245 nm) and 5% sulfuric acid in methanol/Δ. Purification via flash column chromatography was conducted manually using Sigma Aldrich silica gel 60 (0.043–0.063 mm) under a positive pressure of compressed air or via automation using a Büchi Reveleris X2 with pre-packed silica cartridges. Purification via strong ion exchange (SAX) chromatography was conducted on a Bio-Rad Biologic LP system using a Bio-Scale Mini UNOsphere Q (strong anion exchange) cartridge (column volume = 5 mL): flow rate (3.0 mL/min), 0 → 100% 1.0 M NH_4HCO_3 over 33 min. Optical activities were recorded on an automatic Rudolph Autopol I or Bellingham and Stanley ADP430 polarimeter (concentration in g/100mL). ^{1}H NMR spectra were recorded at 400 MHz, ^{13}C NMR spectra at 100 MHz and ^{31}P NMR spectra at 161 MHz respectively using Bruker AV-III spectrometers. ^{1}H NMR resonances were assigned with the aid of gDQCOSY. ^{13}C NMR resonances were assigned with the aid of gHSQCAD. Coupling constants are reported in Hertz. Chemical shifts (δ, in ppm) are standardised against the deuterated solvent peak. NMR data were analysed using Mestrenova or iNMR software. ^{1}H NMR splitting patterns were assigned as follows: br. s (broad singlet), s (singlet), d (doublet), dd (doublet of doublets), ddd (doublet of doublet of doublets), app. t (apparent triplet), t (triplet), quartet (q) or m (multiplet and/or multiple resonances). HRMS (ESI) were obtained on Agilent 6530 Q-TOF, LQT Orbitrap XL1 or Waters (Xevo, G2-XS TOF or G2-S ASAP) Micromass LCT spectrometers using a methanol mobile phase in positive/negative ionisation modes, as appropriate.

S2. Experimental procedures for compounds 2-21

S2.1. Compound 2

To a stirred solution of **1** (100 mg, 0.21 mmol, 1.0 equiv), PyBOP (280 mg, 0.53 mmol, 2.5 equiv) and DIPEA (75 µL, d = 0.742, 0.43 mmol, 2.0 equiv) in CH_2Cl_2 (2 mL), was added 3-aminopropionitrile (24 µL, d = 0.952, 0.32 mmol, 1.5 equiv) in CH_2Cl_2 (0.1 mL) at 0 °C. The mixture was left stirring for 40 min. and was diluted with CH_2Cl_2 (10 mL). The organic layer was washed 1.0 M aq. HCl (2 × 10 mL), sat. aq. $NaHCO_3$ solution (2 × 10 mL) and brine (10 mL), dried over Na_2SO_4, filtered and concentrated under reduced pressure. Purification using silica gel flash column chromatography, eluting with EtOAc/toluene (0/100, 5/95, 10/90, 20/80) afforded **2** as a white solid (51 mg, 0.1 mmol, 47%). R_f 0.29 (EtOAc/toluene, 3/7); $[\alpha]_D^{22}$ +56.4 (*c*. 7.5, $CHCl_3$); mp: 102-105 °C; **^{1}H NMR** (400 MHz; $CDCl_3$) δ 7.40 – 7.29 (15 H, m, Ar-H), 6.87 (1 H, t, *J* = 6.1 Hz, C(O)N(*H*)CH_2CH_2C≡N), 5.45 (1 H, d, *J* = 1.5 Hz, H_1), 4.88 (1 H, d, *J* = 12.0 Hz, C*H*$_2$Ph), 4.72 (1 H, d, *J* = 12.0 Hz, C*H*$_2$Ph), 4.68 (1 H, d, *J* = 12.0 Hz, C*H*$_2$Ph), 4.64 (1 H, d, *J* = 12.0 Hz, C*H*$_2$Ph), 4.53 (1 H, d, *J* = 9.8 Hz, H_5), 4.38 (1 H, s, C4-*OH*), 4.26 (1 H, app. t, *J* = 9.5 Hz, H_4), 3.94 (1 H, dd, *J* = 2.8, 1.8 Hz, H_2), 3.77 (1 H, dd, *J* = 9.3, 3.0 Hz, H_3), 3.64 (1 H, td, *J* = 12.6, 6.2 Hz, C(O)N(H)C*H*$_2$$CH_2$C≡N), 3.39 (1 H, ddt, *J* = 13.8, 7.8, 5.9 Hz, C(O)N(H)C*H*$_2$$CH_2$CN), 2.66 (1 H, dd, *J* = 11.7, 5.0 Hz, C(O)N(H)CH_2C*H*$_2$CN), 2.61 – 2.51 (1 H, m, C(O)N(H)CH_2C*H*$_2$C≡N); **^{13}C NMR** (101 MHz; $CDCl_3$) δ 172.1 (*C*(O)N(H)CH_2CH_2C≡N), 138.4 (C_q), 137.8 (C_q), 132.8 (C_q), 132.4, 129.4, 128.5, 128.4, 128.3, 128.0, 127.8, 127.7, 117.4 (C(O)N(H)CH_2CH_2*C*≡N), 86.8 (C1), 78.5 (C3), 77.2 (C2), 73.3 (*C*H$_2$Ph), 73.1 (*C*H$_2$Ph), 70.9 (C5), 69.8 (C4), 35.1 (C(O)N(H)*C*H$_2$$CH_2$C≡N), 18.3 (C(O)N(H)$CH_2$*C*H$_2$C≡N); **HRMS** (ES$^+$) *m/z* [Found: $(M+NH_4)^+$ 536.2217 $C_{29}H_{34}N_3O_5S$ requires $(M+NH_4)^+$, 536.2219]; **IR** ν_{max}/cm^{-1} 3401 (w, N-H_{amide}), 2249 (w, C≡N), 1655, 1530 (s, C=O_{amide}), 1496, 1454 (m, C=$C_{aromatic}$), 1102 (C-N).

S2.1.1. Elimination byproduct 3

Elimination by-product **3** was isolated from the crude mixture containing **2** and as a colourless oil (46 mg, 90 µmol, 44%). R_f 0.32 (EtOAc/toluene, 3/7); $[\alpha]_D^{22}$ +72.0 (*c*. 7.5, $CHCl_3$); **^{1}H NMR** (400 MHz; $CDCl_3$) δ 7.49 – 7.28 (15 H, m, Ar-H), 6.73 (1 H, t, *J* = 6.2 Hz, C(O)N(*H*)CH_2CH_2C≡N), 6.23 (1 H, dd, *J* = 3.5, 0.9 Hz, H_4), 5.59 (1 H, d, *J* = 5.3 Hz, H_1), 4.70 (1 H, d, *J* = 12.1 Hz, C*H*$_2$Ph), 4.69 (1 H, d, *J* = 12.0 Hz, C*H*$_2$Ph), 4.64 (1 H, d, *J* = 12.1 Hz, C*H*$_2$Ph), 4.60 (1 H, d, *J* = 11.9 C*H*$_2$Ph), 4.29 (1 H, app. t, *J* = 3.8 Hz, H_3), 3.85 (1 H, ddd, *J* = 5.1, 4.1, 0.9 Hz, H_2), 3.62 – 3.43 (2 H, m, C(O)N(H)C*H*$_2$$CH_2$C≡N), 2.69 – 2.52 (2 H, m, C(O)N(H)CH_2C*H*$_2$C≡N); **^{13}C NMR** (101 MHz; $CDCl_3$) δ 161.5 (*C*(O)N(H)CH_2CH_2C≡N), 143.6 (C5), 137.8 (C_q), 137.5 (C_q), 133.4 (C_q), 131.9, 129.4, 128.6, 128.5, 128.5, 128.1, 128.0, 127.9, 127.8, 117.9 (C(O)N(H)CH_2CH_2*C*≡N), 106.1 (C4), 85.1 (C1), 72.81 (C2), 72.5 (*C*H$_2$Ph), 71.2 (*C*H$_2$Ph), 68.3 (C3), 35. 6 (C(O)N(H)*C*H$_2$$CH_2$C≡N), 18.2 (C(O)N(H)$CH_2$*C*H$_2$C≡N); **HRMS** (ES$^+$) *m/z* [Found:

$(M+NH_4)^+$ 518.2115 $C_{29}H_{32}N_3O_4S$ requires $(M+NH_4)^+$, 518.2114]; **IR** ν_{max}/cm^{-1} 3354 (w, N-H_{amide}), 2248 (w, C≡N), 1655, 1517 (s, C=O_{amide}), 1454 (m, C=$C_{aromatic}$), 1057 (C-N).

S2.2. Compound 4

In a manner similar to [1]: to a mixture of **2** (50 mg, 0.1 mmol, 1.0 equiv), imidazole (20 mg, 0.29 mmol, 3.0 equiv) and DMAP (5.9 mg, 50 µmol, 0.5 equiv) in DMF (1 mL) was added TBDMSOTf (66 µL, d = 1.151, 0.29 mmol, 3.0 equiv) dropwise. The reaction mixture was left stirring overnight at room temperature and was quenched with H_2O (0.1 mL). The mixture was concentrated under reduced pressure, and the remaining crude was reconstituted in CH_2Cl_2 (10 mL) and H_2O (5 mL). The organic layer was washed, separated, dried over Na_2SO_4, filtered and concentrated under reduced pressure to furnish a colourless oil. Purification by silica gel flash column chromatography, eluting with EtOAc/hexane (0/100, 10/90, 20/80) afforded **4** as a white solid (50 mg, 79 µmol, 80%). R_f 0.46 (EtOAc/hexane, 1/2); $[\alpha]_D^{26}$ +14.0 (*c*. 1.0, $CHCl_3$); mp: 119-122 °C; **^{1}H NMR** (400 MHz; $CDCl_3$) δ 7.67 – 7.23 (15 H, m, Ar-H), 6.29 (1 H, t, *J* = 6.1 Hz, C(O)N(*H*)CH_2CH_2C≡N), 5.35 (1 H, d, *J* = 7.4 Hz, H_1), 4.57 (1 H, d, *J* = 12.1 Hz, C*H*$_2$Ph), 4.56 (1 H, d, *J* = 11.8 Hz, C*H*$_2$Ph), 4.50 (1 H, app. t, *J* = 3.4 Hz, H_4), 4.49 (1 H, d, *J* = 11.8 Hz, C*H*$_2$Ph), 4.47 (1 H, d, *J* = 12.0 Hz, C*H*$_2$Ph), 4.19 (1 H, d, *J* = 3.9 Hz, H_5), 3.81 (1 H, dd, *J* = 7.3, 2.6 Hz, H_2), 3.56 (1 H, dd, *J* = 5.2, 2.6 Hz, H_3), 3.28 (1 H, dq, *J* = 13.1, 6.6 Hz, C(O)N(H)C*H*$_2$$CH_2$C≡N), 3.19 (1 H, td, *J* = 13.2, 6.5 Hz, C(O)N(H)C*H*$_2$$CH_2$C≡N), 2.31 (1 H, dt, *J* = 16.6, 6.6 Hz, C(O)N(H)CH_2C*H*$_2$C≡N), 2.25 – 2.12 (1 H, m, C(O)N(H)CH_2C*H*$_2$C≡N), 0.80 (9 H, s, C(C*H*$_3$)$_3$), 0.00 (3 H, s, Si(C*H*$_3$)$_2$), -0.08 (3 H, s, Si(C*H*$_3$)$_2$); **^{13}C NMR** (101 MHz; $CDCl_3$) δ 169.3 (*C*(O)N(H)CH_2CH_2C≡N), 137.9 (C_q), 137.8 (C_q), 133.5 (C_q), 133.0, 129.2, 128.4, 128.3, 128.2, 128.1, 128.0, 127.9, 127.8, 117.6 (C(O)N(H)CH_2CH_2*C*≡N), 84.1 (C1), 77.5 (C5), 74.2 (C2), 72.4 (*C*H_2Ph), 72.4 (*C*H_2Ph), 68.7 (C4), 35.3 (C(O)N(H)*C*H_2CH_2C≡N), 25.8 (C(*C*H_3)$_3$), 18.0 (*C*(CH_3)$_3$), -4.8 (Si(C*H*$_3$)$_2$), -5.0 (Si(C*H*$_3$)$_2$); **HRMS** (ES^+) *m/z* [Found: $(M+NH_4)^+$ 650.3082 $C_{35}H_{48}N_3O_5SSi$ requires $(M+NH_4)^+$, 650.3078]; **IR** ν_{max}/cm^{-1} 3217 (w, N-H_{amide}), 2255 (w, C≡N), 1678, 1659 (s, C=O_{amide}), 1496, 1455 (m, C=$C_{aromatic}$), 1243 (s, Si-C), 1096 (s, C-N), 1068 (s, Si-O).

S2.3. Compound 5

Propionitrile **4** (60 mg, 0.11 mmol, 1.0 equiv) was dissolved in toluene (10 mL) and $TMSN_3$ (84 µL, d = 0.872, 0.64 mmol, 6.0 equiv) and Bu_2SnO (11 mg, 43 µmol, 0.4 equiv) were added. The mixture was heated to 120 °C and stirred for 16 h. Upon completion, the mixture was cooled down to room temperature, diluted with EtOAc (50 mL) and washed with 0.1 M aq. HCl solution (50 mL). The organic layer was dried over Na_2SO_4, filtered and concentrated under reduced pressure. Purification of this crude material by silica gel flash column chromatography, eluting with MeOH/CH_2Cl_2 (0/100, 1/99, 2/98) afforded **5** as a brown oil (34 mg, 56 µmol, 51%). R_f 0.71 (MeOH/CH_2Cl_2, 1/2); $[\alpha]_D^{22}$ +88.7 (*c*. 1.75, $CHCl_3$); **^{1}H NMR** (400 MHz; $CDCl_3$) δ 7.47 – 7.26 (15 H, m, Ar-H), 5.64 (1 H, d,

J = 8.9 Hz, H_5), 5.54 (1 H, d, J = 1.8 Hz, H_1), 4.74 (1 H, d, J = 11.7 Hz, CH_2Ph), 4.73 (1 H, d, J = 11.8Hz, CH_2Ph), 4.69 (1 H, d, J = 11.7 Hz, CH_2Ph), 4.65 (1 H, d, J = 11.8 Hz, CH_2Ph), 4.41 (1 H, app. t, J = 8.8 Hz, H_4), 4.09 (1 H, app. t, J = 2.6 Hz, H_2), 3.84 (1 H, dd, J = 8.6, 2.8 Hz, H_3), 0.78 (9 H, s, C(CH_3)$_3$), 0.00 (3 H, s, Si(CH_3)$_2$), -0.41 (3 H, s, Si(CH_3)$_2$); **^{13}C NMR** (101 MHz; $CDCl_3$) δ 155.8 (C_q tetrazole), 137.6 (C_q), 137.1 (C_q), 132.8 (C_q), 132.2, 129.2, 128.6, 128.5, 128.3, 128.1, 128.0, 86.8 (C1), 79.7 (C3), 76.5 (C2), 73.3 (CH_2Ph), 72.7 (CH_2Ph), 71.0 (C4), 68.5 (C5), 25.6 (C(CH_3)$_3$), 17.9 (C(CH_3)$_3$), -4.3 (Si(CH_3)$_2$), -5.9 (Si(CH_3)$_2$); **HRMS** (ES^+) *m/z* [Found: $(M+H)^+$ 605.2628 $C_{32}H_{41}N_4O_4SSi$ requires $(M+H)^+$,605.2618].

S2.4. Compound 7

S2.4.1. Phenyl 2,3-di-*O*-benzyl-6-*O*-benzoyl-1-thio-α-D-mannopyranoside

To a stirred solution of **6**[1] (1.0 g, 2.21 mmol, 1.0 equiv), pyridine (357 µL, d = 0.978, 4.42 mmol, 2.0 equiv), DMAP (81 mg, 0.7 mmol, 0.3 equiv) in CH_2Cl_2 (10 mL) was added BzCl dropwise (269 µL, d = 1.211, 2.32 mmol, 1.05 equiv) at 0 °C. The reaction was left stirring overnight at room temperature, and diluted with CH_2Cl_2 (15 mL). The mixture was washed with 1.0 M aq. HCl (10 mL), sat. aq. $NaHCO_3$ (10 mL) and brine (10 mL). The organic layer was dried over $MgSO_4$, filtered and concentrated under reduced pressure to afford the crude product. Purification by Reveleris® automated silica gel flash column chromatography (liquid injection onto column), eluting with EtOAc/hexane (0/100, 5/95 and 10/90) afforded phenyl 2,3-di-*O*-benzyl-6-*O*-benzoyl-1-thio-α-D-mannopyranoside as a colourless oil (1.1 g, 2.0 mmol, 90%). R_f 0.37 (EtOAc/hexane, 1/2); $[\alpha]_D^{22}$ +48.2 (*c*. 7.5, $CHCl_3$); **^{1}H NMR** (400 MHz; $CDCl_3$) δ 8.03 – 7.19 (20 H, m, Ar-H), 5.65 (1 H, d, J = 1.3 Hz, H_1), 4.69 (1 H, d, J = 12.1 Hz, CH_2Ph-attached to C2), 4.65 – 4.59 (2 H, m, $H_{6a,b}$), 4.61 (1 H, d, J = 12.7 Hz, CH_2Ph-attached to C2), 4.55 (1 H, d, J = 10.5 Hz, CH_2Ph-attached to C3), 4.52 (1 H, d, J = 10.1 Hz, CH_2Ph-attached to C3), 4.43 (1 H, dt, J = 9.6, 3.9 Hz, H_5), 4.15 (1 H, dd, J = 9.6 Hz, H_4), 4.04 (1 H, dd, J = 3.0, 1.6 Hz, H_2), 3.73 (1 H, dd, J = 9.5, 3.0 Hz, H_3), 2.64 (1 H, br. s, C4-O*H*); **^{13}C NMR** (101 MHz; $CDCl_3$) δ 166.7 (*C*(O)Ph), 137.8 (C_q), 137.7 (C_q), 134.1 (C_q), 133.0 (C_q), 131.5, 130.1, 129.8, 129.1, 128.6, 128.4, 128.3, 128.1, 128.0, 127.8, 127.5, 85.7 (C1), 79.6 (C3), 75.7 (C2), 72.1 (CH_2Ph), 71.9 (CH_2Ph), 71.7 (C5), 66.9 (C4), 64.1 (C6); **HRMS** (ES^+) *m/z* [Found: $(M+NH_4)^+$ 574.2257 $C_{33}H_{32}O_6SNH_4$ requires $(M+NH_4)^+$, 574.2258]; **IR** ν_{max}/cm^{-1} 3477 (br. s, C4-*OH*), 1718 (s, C=O_{ester}), 1273 (s, C-O_{ester}), 1070 (s, C-O_{ether}), 1025 (s, C-OH).

S2.4.2. Phenyl 2,3-di-*O*-benzyl-4-*O*-*tert*-butyldimethylsilyl-6-*O*-benzoyl-1-thio-α-D-mannopyranoside

In a manner similar to [1]: To a mixture of phenyl 2,3-di-*O*-benzyl-6-*O*-benzoyl-1-thio-α-D-mannopyranoside (900 mg, 1.62 mmol, 1.0 equiv), imidazole (330 mg, 4.85 mmol, 3.0 equiv) and DMAP (99 mg, 0.81 mmol, 0.5 equiv) in DMF (10 mL) was added TBDMSOTf (1.1 mL, d = 1.151, 4.85 mmol, 3.0 equiv) dropwise. The reaction mixture

was left stirring overnight at room temperature and was quenched with H_2O (2 mL). The mixture was concentrated under reduced pressure, and the remaining crude was reconstituted in CH_2Cl_2 (50 mL) and H_2O (30 mL). The organic layer was washed, separated, dried over $MgSO_4$, filtered and concentrated under reduced pressure to furnish a colourless oil. Purification by silica gel flash column chromatography, eluting with EtOAc/hexane (0/100, 5/95, 10/90) afforded phenyl 2,3-di-*O*-benzyl-4-*O*-*tert*-butyldimethylsilyl-6-*O*-benzoyl-1-thio-α-D- mannopyranoside, as a colourless oil (846 mg, 1.27 mmol, 78%). R_f 0.75 (EtOAc/hexane, 1/2); $[\alpha]_D^{26}$ +57.8 (*c*. 1.37, $CHCl_3$); **^{1}H NMR** (400 MHz; $CDCl_3$) δ 7.96 – 7.10 (20 H, m, Ar-H), 5.54 (1 H, d, *J* = 1.7, H_1), 4.61 (1 H, dd, *J* = 11.6, 1.8 Hz, H_{6b}), 4.57 (1 H, d, *J* = 12.0 Hz, CH_2Ph), 4.57 (1 H, s, *J* = 11.9 Hz, CH_2Ph), 4.52 (1 H, d, *J* = 11.8 Hz, CH_2Ph), 4.50 (1 H, d, *J* = 12.0 Hz, CH_2Ph), 4.39 (1 H, dd, *J* = 11.6, 5.8 Hz, H_{6a}), 4.35 – 4.29 (1 H, m, H_5), 4.19 (1 H, t, *J* = 9.1 Hz, H_4), 3.91 (1 H, dd, *J* = 2.7, 2.0 Hz, H_2), 3.61 (1 H, dd, *J* = 8.9, 2.9 Hz, H_3), 0.82 (9 H, s, C(CH_3)$_3$), 0.00 (6 H, d, *J* = 1.5 Hz, Si(CH_3)$_2$); **^{13}C NMR** (101 MHz; $CDCl_3$) δ 166.4 (*C*(O)Ph), 138.1 (C_q), 138.1 (C_q), 132.8 (C_q), 131.3 (C_q), 130.1, 129.7, 129.0, 128.3, 128.3, 127.9, 127.6, 127.6, 127.5, 127.3, 85.7 (C1), 80.3 (C3), 76.2 (C2), 72.6 (C5), 72.1 (*C*H_2Ph), 71.8 (*C*H_2Ph), 68.2 (C4), 64.2 (C6), 26.0 (C(*C*H_3)$_3$), 18.2 (*C*(CH$_3$)$_3$), -3.8 (Si(*C*H_3)$_2$), -5.0 (Si(*C*H_3)$_2$); **HRMS** (ES$^+$) *m/z* [Found: $(M+NH_4)^+$ 688.3127 $C_{39}H_{50}NO_6SSi$ requires $(M+NH_4)^+$, 688.3123]; **IR** ν_{max}/cm^{-1} 1713 (s, C=O$_{ester}$), 1276 (m, C-O$_{ester}$), 1253 (m, Si-C), 1096 (s, Si-O), 1024 (m, C-O$_{ether}$).

S2.4.3. Compound 7

In a manner similar to [1]: To a stirred solution of phenyl 2,3-di-*O*-benzyl-4-*O*-*tert*-butyldimethylsilyl-6-*O*-benzoyl-1-thio-α-D- mannopyranoside (800 mg, 1.19 mmol, 1.0 equiv) in anhydrous MeOH and THF (7 mL, 1/1 *v*/*v*), Na (14 mg, 0.60 mmol, 0.5 equiv) dissolved in anhydrous MeOH (2 mL) was added dropwise at room temperature. The mixture was stirred overnight, then neutralised with ion exchange Amberlite 120 (H^+) resin (approximately 0.7 g, 10 min), filtered, and concentrated under reduced pressure. Purification by silica gel flash column chromatography, eluting with Et_2O/hexane (0/100, 5/95, 10/90) afforded **7** as a colourless oil (596 mg, 1.07 mmol, 90%). R_f 0.69 (EtOAc/hexane, 1/2); $[\alpha]_D^{26}$ +81.3 (*c*. 1.0, $CHCl_3$); **^{1}H NMR** (400 MHz; $CDCl_3$) δ 7.35 – 7.13 (15 H, m, Ar-H), 5.37 (1 H, d, *J* = 1.8 Hz, H_1), 4.51 (2 H, d, *J* = 12.5 Hz, CH_2Ph), 4.49 (1 H, d, *J* = 11.8 Hz, CH_2Ph), 4.44 (1 H, d, *J* = 11.9 Hz, CH_2Ph), 4.02 – 3.96 (1 H, m, H_4), 3.96 – 3.91 (1 H, m, H_5), 3.82 (1 H, dd, *J* = 2.8, 2.0 Hz, H_2), 3.72 (1 H, ddd, *J* 11.5, 6.6, 2.4 Hz, H_{6b}), 3.64 (1 H, ddd, *J* = 11.6, 6.5, 5.2 Hz, H_{6a}), 3.53 (1 H, dd, *J* = 8.4, 2.9 Hz, H_3), 1.70 (1 H, t, *J* = 6.6 Hz, C6-*OH*), 0.78 (9 H, s, C(CH_3)$_3$), 0.00 (3 H, s, Si(CH_3)$_2$), -0.05 (3 H, s, Si(CH_3)$_2$); **^{13}C NMR** (101 MHz; $CDCl_3$) δ 138.1 (C_q), 138.0 (C_q), 134.0 (C_q), 132.0, 129.1, 128.4, 128.3, 127.9, 127.7, 127.7, 127.6, 86.2 (C1), 80.4 (C3), 76.4 (C2), 74.8 (C5), 72.5 (*C*H_2Ph), 72.0 (*C*H_2Ph), 67.9 (C4), 62.2 (C6), 26.0 (C(*C*H_3)$_3$), 18.2 (*C*(CH_3)$_3$), -3.8 (Si(*C*H_3)$_2$), -4.9 (Si(*C*H_3)$_2$); **HRMS** (ES$^+$) *m/z* [Found: $(M+NH_4)^+$ 584.2875

$C_{32}H_{46}NO_5SSi$ requires $(M+NH_4)^+$, 584.2850]; **IR** ν_{max}/cm^{-1} 1454 (w, $C=C_{aromatic}$), 1248 (m, C-Si), 1084 (s, Si-O).

S2.5. C-6 oxime thioglycoside 8

S2.5.1. C6-aldehyde thioglycoside intermediate

To a stirred solution of **7** (60 mg, 0.11 mmol, 1.0 equiv) in DMSO (1 mL) was added Et_3N (44 µL, d = 0.726, 0.32 mmol, 3.0 equiv) and sulfur trioxide pyridine complex (51 mg, 0.32 mmol, 3.0 equiv) at room temperature. The reaction mixture was left stirring for 1 h before it was diluted with EtOAc (30 mL) and H_2O (20 mL). The whole was extracted with EtOAc (3 × 15 mL) and the extracts were washed with H_2O (6 × 20 mL) and brine (20 mL). The organic layer was dried over Na_2SO_4, filtered and concentrated under reduced pressure. The crude aldehyde was obtained as a colourless oil (60 mg, 0.11 mmol, 98%) and was used immediately in the next step, without further purification. R_f 0.684 (EtOAc/hexane, 1/2); $[\alpha]_D^{22}$ -14.4 (*c*. 0.33, $CHCl_3$); **^{1}H NMR** (400 MHz; $CDCl_3$) δ 9.77 (1 H, s, C*H*O), 7.56 – 7.25 (15 H, m, Ar-H), 5.56 (1 H, d, *J* = 6.4 Hz, H_1), 4.57 (1 H, d, *J* = 11.9 Hz, C*H*$_2$Ph), 4.52 (2 H, s, C*H*$_2$Ph), 4.43 (1 H, d, *J* = 11.9 Hz, C*H*$_2$Ph), 4.26 – 4.20 (2 H, m, H_4, H_5), 3.83 (1 H, dd, *J* = 6.2, 2.3 Hz, H_2), 3.60 (1 H, dd, *J* = 5.8, 2.5 Hz, H_3), 0.82 (9 H, s, $C(CH_3)_3$), 0.00 (3 H, s, $Si(CH_3)_2$), -0.07 (3 H, s, $Si(CH_3)_2$); **^{13}C NMR** (101 MHz; $CDCl_3$) δ 198.0 (*C*HO), 137.8 (C_q), 137.6 (C_q), 133.5 (C_q), 132.4, 131.8, 129.0, 128.5, 128.4, 128.0, 127.9, 127.8, 127.5, 83.8 (C1), 81.15 (C5), 77.2 (C3), 73.8 (C2), 72.5 (*C*H$_2$Ph), 72.3 (*C*H$_2$Ph), 68.9 (C4), 25.7 ($C(CH_3)_3$), 18.0 ($C(CH_3)_3$), -4.6 ($Si(CH_3)_2$), -5.0 ($Si(CH_3)_2$); **HRMS** (ES$^+$) *m/z* [Found: $(M+NH_4)^+$ 582.2723 $C_{32}H_{44}NO_5SSi$ requires $(M+NH_4)^+$, 582.2704].

S2.5.2. C-6 oxime thioglycoside 8

The crude C-6 aldehyde (4.5 g, 7.97 mmol, 1.0 equiv) was dissolved in THF (790 mL) and a solution of $H_2NOH{\cdot}HCl$ (554 mg, 7.97 mmol, 1.0 equiv) dissolved in H_2O (15 mL) was added dropwise. The mixture was cooled to 0 °C and a solution of Na_2CO_3 (1.0 g, 9.56 mmol, 1.2 equiv) dissolved in H_2O (9.5 mL) was added dropwise. The solution was slowly warmed to room temperature and stirred for 24 h. The mixture was diluted with H_2O (30 mL) and then extracted with EtOAc (4 × 300 mL). The organic layers were combined, dried over Na_2SO_4, filtered and concentrated under reduced pressure. The obtained crude oil was purified using silica gel flash column chromatography, eluting with Et_2O/petroleum ether (0/100, 5/95, 10/90, 20/80) to furnish **8** as a colourless oil. *Cis* and *trans* (1/6.7) were isolated separately (major isomer: 2.82 g, 4.86 mmol, 71%, minor isomer: 416 mg, 0.72 mmol, 9%) and both were used in the next step; Major isomer R_f 0.78; minor isomer R_f 0.68; (EtOAc/petroleum ether, 1/2); major: $[\alpha]_D^{22}$ +57.7 (*c*. 0.46, $CHCl_3$); minor: $[\alpha]_D^{22}$ +41.3 (*c*. 1.0, $CHCl_3$); **^{1}H NMR** (400 MHz; $CDCl_3$) Major isomer δ 7.46 – 7.17 (16 H, m, Ar-H, *H*C=N), 5.40 (1 H, d, *J* = 1.8 Hz, H_1), 4.58 (1 H, d, *J* = 10.5 Hz, C*H*$_2$Ph), 4.57 (1 H, m, H_5), 4.55 (1 H, d, *J* = 10.1 Hz, C*H*$_2$Ph), 4.54 (1 H, d, *J* = 12.4

Hz, C*H*$_2$Ph), 4.50 (1 H, d, *J* = 11.9 Hz, C*H*$_2$Ph), 4.06 (1 H, app. t, *J* = 9.0 Hz, H$_4$), 3.89 – 3.86 (1 H, m, H$_2$), 3.59 (1 H, dd, *J* = 8.9, 2.9 Hz, H$_3$), 0.81 (9 H, s, C(C*H$_3$*)$_3$), 0.00 (3 H, s, Si(C*H$_3$*)$_2$), -0.01 (3 H, s, Si(C*H$_3$*)$_2$); **^{13}C NMR** (101 MHz; CDCl$_3$) δ 149.3 (H*C*=N), 138.1 (C$_q$), 137.9 (C$_q$), 134.0 (C$_q$), 131.9, 129.1, 128.4, 128.4, 127.9, 127.85, 127.8, 127.7, 127.6, 86.3 (C1), 79.8 (C3), 76.3 (C2), 72.5 (C5), 72.5 (*C*H$_2$Ph), 72.2 (*C*H$_2$Ph), 69.8 (C4), 25.8 (C(*CH$_3$*)$_3$), 18.1 (*C*(CH$_3$)$_3$), -4.0 (Si(*C*H$_3$)$_2$), -4.6 (Si(*C*H$_3$)$_2$); **HRMS** (ES$^+$) *m/z* [Found: (M+NH$_4$)$^+$ 597.2815 $C_{32}H_{45}N_2O_5SSi$ requires (M+NH$_4$)$^+$, 597.2813].

S2.6. C-6 nitrile thioglycoside 9

Oxime **8** (120 mg, 0.21 mmol, 1.0 equiv) was dissolved in dry acetonitrile (21 mL) and POCl$_3$ (19 µL, d = 1.645, 0.21 mmol, 1.0 equiv) was added at room temperature. The solution was stirred for 5 min. at room temperature, heated up to 65 °C and then stirred for 3 h. The reaction was quenched with sat. aq. NaHCO$_3$ solution (20 mL) and extracted with EtOAc (3 × 60 mL). The organic layers were combined, dried over Na$_2$SO$_4$, filtered and concentrated under reduced pressure. The crude product was purified using silica gel flash column chromatography, eluting with Et$_2$O/petroleum ether (0/100, 5/95, 10/90, 20/80) to furnish **9** as a yellow oil (47 mg, 84 µmol, 40%). R$_f$ 0.90 (EtOAc/hexane, 1/2); $[\alpha]_D^{22}$ +39.4 (*c*. 0.53, CHCl$_3$); **^{1}H NMR** (400 MHz; CDCl$_3$) δ 7.44 – 7.21 (15 H, m, Ar-H), 5.45 (1 H, d, *J* = 3.0 Hz, H$_1$), 4.76 (1 H, d, *J* = 8.3 Hz, H$_5$), 4.60 (1 H, d, *J* = 11.9 Hz, C*H*$_2$Ph), 4.58 (1 H, d, *J* = 12.1 Hz, C*H*$_2$Ph), 4.55 (1 H, d, *J* = 12.1 Hz, C*H*$_2$Ph), 4.53 (1 H, d, *J* = 12.0 Hz, C*H*$_2$Ph), 4.21 (1 H, app. t, *J* = 8.2 Hz, H$_4$), 3.84 (1 H, app. t, *J* = 2.9 Hz, H$_2$), 3.49 (1 H, dd, *J* = 8.2, 2.9 Hz, H$_3$), 0.89 (9 H, s, C(C*H$_3$*)$_3$), 0.18 (3 H, s, Si(C*H$_3$*)$_2$), 0.05 (3 H, s, Si(C*H$_3$*)$_2$); **^{13}C NMR** (101 MHz; CDCl$_3$) δ 137.5 (C$_q$), 137.1 (C$_q$), 132.9 (C$_q$), 131.5, 129.4, 129.3, 129.1, 128.5, 128.5, 128.4, 128.2, 128.1, 128.0, 127.9, 127.9, 127.6, 127.2, 124.4, 117.0 (*C*≡N), 85.9 (C1), 78.8 (C3), 75.3 (C2), 72.6 (*C*H$_2$Ph), 72.5 (*C*H$_2$Ph), 69.4 (C4), 64.7 (C5), 25.8 (C(*CH$_3$*)$_3$), 18.0 (*C*(C*H$_3$*)$_3$), -4.1 (Si(*C*H$_3$)$_2$), -4.8 (Si(*C*H$_3$)$_2$); **HRMS** (ES$^+$) *m/z* [Found: (M+NH$_4$)$^+$ 579.2732 $C_{32}H_{43}N_2O_4SSi$ requires (M+NH$_4$)$^+$, 579.2707].

S2.6.1. 4-postion deprotected byproduct 10

Alcohol **10** was also isolated as a yellow oil (24 mg, 54 µmol, 26%) from the crude mixture containing **9**. R$_f$ 0.82 (EtOAc/hexane, 1/2); $[\alpha]_D^{22}$ +15.4 (*c*. 0.95, CHCl$_3$); **^{1}H NMR** (400 MHz; CDCl$_3$) δ 7.41 – 7.28 (15 H, m, Ar-H), 5.51 (1 H, d, *J* = 1.7 Hz, H$_1$), 4.87 (1 H, d, *J* = 9.8 Hz, H$_5$), 4.66 (1 H, d, *J* = 12.0 Hz, C*H*$_2$Ph), 4.60 (1 H, d, *J* = 12.0 Hz, C*H*$_2$Ph), 4.57 (1 H, d, *J* = 12.2 Hz, C*H*$_2$Ph), 4.55 (1 H, d, *J* = 11.8 Hz, C*H*$_2$Ph), 4.32 (1 H, app. t, *J* = 9.6 Hz, H$_4$), 3.96 (1 H, dd, *J* = 2.7, 2.0 Hz, H$_2$), 3.57 (1 H, dd, *J* = 9.3, 2.9 Hz, H$_3$), 2.92 (1 H, br. s, C4-*OH*); **^{13}C NMR** (101 MHz; CDCl$_3$) δ 137.3 (C$_q$), 137.2 (C$_q$), 132.7 (C$_q$), 131.5, 129.4, 128.7, 128.6, 128.3, 128.3, 128.1, 128.0, 128.0, 116.6 (*C*≡N), 86.6 (C1), 78.4 (C3), 75.4 (C2), 72.5 (*C*H$_2$Ph), 72.4 (*C*H$_2$Ph), 68.3 (C4), 63.1 (C3); **HRMS** (ES$^+$) *m/z* [Found: (M+NH$_4$)$^+$ 465.1857 $C_{26}H_{29}N_2O_4S$ requires (M+NH$_4$)$^+$, 465.1843].

S2.7. PMB-protected C-6 tetrazole thioglycosides 11 and 12

To a stirred solution of **5** (130 mg, 0.21 mmol, 1.0 equiv) in DMF (2 mL) was added successively, KI (53 mg, 0.32 mmol, 1.5 equiv), K_2CO_3 (44 mg, 0.32 mmol, 1.5 equiv) and PMBCl (58 µL, d = 1.155, 0.43 mmol, 2.0 equiv). The reaction was left stirring for 4 h and was diluted with CH_2Cl_2 (10 mL). The organic layer was washed with 10% aq. $Na_2S_2O_3$ solution (10 mL) and brine (10 mL), dried over $MgSO_4$, filtered and concentrated under reduced pressure. The crude product was purified using silica gel flash column chromatography, eluting with acetone/toluene (1/250, 1/150, 1/100) to furnish isomers **11** and **12** (80 mg, 0.11 mmol, 53%) as oils.

S2.7.1. Compound 11

N_1-regioisomer **11** was isolated as a yellow oil (42 mg, 58 µmol, 28%). R_f 0.42 (acetone/toluene, 1/50); $[\alpha]_D^{22}$ +25.4 (*c*. 0.53, $CHCl_3$); **^{1}H NMR** (400 MHz; $CDCl_3$) δ 7.56 – 7.39 (16 H, m, Ar-H), 7.36 (2 H, d, *J* = 8.7 Hz, Ar-H PMB), 6.82 (2 H, d, *J* = 8.8 Hz, Ar-H PMB), 5.76 (1 H, d, *J* = 1.8 Hz, H_1), 5.68 (1 H, d, *J* = 9.4 Hz, H_5), 5.66 (1 H, d, *J* = 15.0 Hz, C*H*$_2$Ph-PMB), 5.63 (1 H, d, *J* = 15.0 Hz, C*H*$_2$Ph-PMB), 4.83 (1 H, d, *J* = 11.6 Hz, C*H*$_2$Ph-attached to C2), 4.81 (1 H, d, *J* = 12.0 Hz, C*H*$_2$Ph-attached to C3), 4.78 (1 H, d, *J* = 11.1 Hz, C*H*$_2$Ph-attached to C2), 4.75 (1 H, d, *J* = 11.7 Hz, C*H*$_2$Ph-attached to C3), 4.59 (1 H, app. t, *J* = 9.4 Hz, H_4), 4.22 – 4.19 (1 H, m, H_2), 3.87 (3 H, s, OC*H*$_3$), 3.86 – 3.84 (1 H, m, H_3), 0.79 (9 H, s, C(C*H*$_3$)$_3$), 0.00 (3 H, s, Si(C*H*$_3$)$_2$), -0.53 (3 H, s, Si(C*H*$_3$)$_2$); **^{13}C NMR** (101 MHz; $CDCl_3$) δ 159.7 (C_q PMB), 152.0 (C_q tetrazole), 137.8 (C_q), 137.5 (C_q), 133.5 (C_q), 131.1 (C_q), 129.9, 129.2, 128.5, 128.4, 128.0, 127.9, 127.8, 127.7, 125.7, 114.1, 86.5 (C1), 80.1 (C3), 76.4 (C2), 72.9 (*C*H$_2$Ph-attached to C2), 72.1 (*C*H$_2$Ph-attached to C3), 70.0 (C4), 67.7 (C5), 55.2 (*C*H$_2$Ph PMB), 50.8 (O*C*H$_3$), 25.6 (C(*C*H$_3$)$_3$), 17.8 (*C*(CH$_3$)$_3$), -4.5 (Si(*C*H$_3$)$_2$), -6.1 (Si(*C*H$_3$)$_2$); **^{13}C-GATED** (101 MHz; $CDCl_3$): 86.5 ($^1J_{C1-H1}$ =172 Hz, C1); **HRMS** (ES$^+$) *m/z* [Found: (M+H)$^+$ 725.3177 $C_{40}H_{49}N_4O_5SSi$ requires (M+H)$^+$, 725.3187].

S2.7.2. Compound 12

N1-regioisomer **12** was isolated as a yellow oil (38 mg, 52 µmol, 25%). R_f 0.48 (acetone/toluene, 1/50); $[\alpha]_D^{22}$ +40.6 (*c*. 0.86, $CHCl_3$); **^{1}H NMR** (400 MHz; $CDCl_3$) δ 7.43 – 7.21 (18 H, m, Ar-H), 6.87 (2 H, d, *J* = 8.7 Hz, Ar-H PMB), 5.67 (1 H, d, *J* = 14.1 Hz, C*H*$_2$Ph-PMB), 5.62 (1 H, d, *J* = 14.0 Hz, C*H*$_2$Ph-PMB), 5.56 (1 H, d, *J* = 1.9 Hz, H_1), 5.40 (1 H, d, *J* = 9.2 Hz, H_5), 4.67 (1 H, d, *J* = 12.3 Hz, C*H*$_2$Ph-attached to C2), 4.60 (1 H, app. t, *J* = 9.1 Hz, H_4), 4.57 (1 H, d, *J* = 12.4 Hz, C*H*$_2$Ph-attached to C2), 4.56 (1 H, d, *J* = 12.9 Hz, C*H*$_2$Ph-attached to C3), 4.52 (1 H, d, *J* = 12.0 Hz, C*H*$_2$Ph-attached to C3), 4.01 – 3.99 (1 H, m, H_2), 3.79 (3 H, s, OC*H*$_3$), 3.70 (1 H, dd, *J* = 8.9, 2.9 Hz, H_3), 0.50 (9H, s, C(C*H*$_3$)$_3$), -0.11 (3 H, s, Si(C*H*$_3$)$_2$), -0.55 (3 H, s, Si(C*H*$_3$)$_2$); **^{13}C NMR** (101 MHz; $CDCl_3$) δ 164.4 (C_q tetrazole), 160.1 (C_q PMB), 138.0 (C_q), 133.9 (C_q), 131.8 (C_q), 130.6 (C_q), 129.0, 128.3,

128.3, 127.8, 127.7, 127.6, 127.5, 125.0, 114.3, 86.5 (C1), 80.2 (C3), 75.7 (C2), 72.1 (CH_2Ph-attached to C2), 71.6 (CH_2Ph-attached to C3), 70.4 (C4), 69.1 (C5), 56.5 (CH_2Ph PMB), 55.3 (OCH_3), 25.5 ($C(CH_3)_3$), 17.7 ($C(CH_3)_3$), -4.1 ($Si(CH_3)_2$), -5.9 ($Si(CH_3)_2$); **^{13}C-GATED** (101 MHz; $CDCl_3$): 86.5 ($^{1}J_{C1\text{-}H1}$ = 168 Hz, C1); **HRMS** (ES^+) *m/z* [Found: $(M+H)^+$ 725.3192 $C_{40}H_{50}N_4O_5SSi$ requires $(M+H)^+$, 725.3187].

S2.8. Compound 11 triethylammonium salt

To a stirred solution of **5** (75 mg, 0.12 mmol, 1.0 equiv) in CH_2Cl_2 (1 mL) was added Et_3N (17 µL, d = 0.726, 0.12 mmol, 1.0 equiv). The reaction was left stirring for 1 h and then was dried *in vacuo*, giving the title compound as a yellow oil (80 mg, 0.11 mmol, 94%). $[\alpha]_D^{22}$ -42.4 (*c*. 0.46, $CHCl_3$); **^{1}H NMR** (400 MHz; $CDCl_3$) δ 7.51 – 7.26 (15 H, m, Ar-H), 5.62 (1 H, d, *J* = 9.3 Hz, H_5), 5.56 (1 H, d, *J* = 1.5 Hz, H_1), 4.77 (1 H, d, *J* = 11.9 Hz, CH_2Ph), 4.74 (1 H, d, *J* = 11.8 Hz, CH_2Ph), 4.71 (1 H, d, *J* = 12.0 Hz, CH_2Ph), 4.67 (1 H, d, *J* = 11.3 Hz, CH_2Ph), 4.53 (1 H, app. t, *J* = 9.1 Hz, H_4), 4.13 (1 H, app. t, *J* = 2.2 Hz), 3.86 (1 H, dd, *J* = 8.9, 2.9 Hz, H_3), 3.05 (6 H, q, *J* = 7.3 Hz, $N(CH_2CH_3)_3$), 1.23 (9 H, t, *J* = 7.3 Hz, $N(CH_2CH_3)_3$), 0.75 (9 H, s, $C(CH_3)_3$), 0.00 (3 H, s, $Si(CH_3)_2$), -0.43 (3 H, s, $Si(CH_3)_2$); **^{13}C NMR** (101 MHz; $CDCl_3$) δ 159.6 (C_q tetrazole), 138.2 (C_q), 138.1 (C_q), 134.3 (C_q), 131.9, 128.9, 128.4, 128.3, 128.1, 127.9, 127.8, 127.6, 127.4, 86.9 (C1), 81.0 (C3), 77.2 (C2), 73.1 (CH_2Ph), 72.3 (CH_2Ph), 71.7 (C4), 69.8 (C5), 45.2 ($N(CH_2CH_3)_3$), 25.7 ($C(CH_3)_3$), 17.9 ($C(CH_3)_3$), 8.5 ($N(CH_2CH_3)_3$), -4.4 ($Si(CH_3)_2$), -5.9 ($Si(CH_3)_2$).

S2.9. Benzyl-protected C-6 tetrazole thioglycosides 13 and 14

To a stirred solution of compound **11** triethylammonium salt (80 mg, 0.11 mmol, 1.0 equiv) in DMF (1.1 mL) was added BnBr (20 µL, d = 1.438, 0.17 mmol, 1.5 equiv). The reaction was left stirring for 3 h and was diluted with CH_2Cl_2 (10 mL). The organic layer was washed with brine (10 mL), dried over $MgSO_4$, filtered and concentrated under reduced pressure. The crude product was purified using silica gel flash column chromatography, eluting with acetone/toluene (1/250, 1/150, 1/100) to furnish the inseparable isomers **13** and **14** as colourless oil in 1/1.2 ratio (24 mg, 34 µmol, 31%). R_f 0.80 (EtOAc/petroleum ether, 1/50); **^{1}H NMR** (400 MHz; $CDCl_3$) δ 7.41 – 7.18 (40 H, m, Ar-H), 5.73 (1 H, d, *J* = 14.3 Hz, CH_2Ph benzyl tetrazole, N_2-isomer), 5.68 (1 H, d, *J* = 14.2 Hz, CH_2Ph benzyl tetrazole, N_2-isomer), 5.57 (1 H, d, *J* = 2.7 Hz, H_1 N_1-isomer), 5.56 (1 H, d, *J* = 2.4 Hz, H_1 N_2-isomer), 5.54 (2 H, d, *J* = 1.9 Hz, CH_2Ph benzyl tetrazole, N_1-isomer), 5.50 (1 H, d, *J* = 9.5 Hz, H_5 N_1-isomer), 5.40 (1 H, d, *J* = 9.2 Hz, H_5 N_2-isomer), 4.67 (1 H, d, *J* = 12.2 Hz, CH_2Ph), 4.65 (1 H, d, *J* = 11.6 Hz, CH_2Ph), 4.63 (1 H, d, *J* = 12.2 Hz, CH_2Ph), 4.61 (1 H, d, *J* = 11.9 Hz, CH_2Ph), 4.60 (1 H, m, H_4 N_2-isomer) 4.59 (1 H, d, *J* = 11.7 Hz, CH_2Ph), 4.57 (1 H, d, *J* = 12.3 Hz, CH_2Ph), 4.54 (2 H, s, CH_2Ph), 4.43 (1 H, app. t, *J* = 9.1 Hz, H_4 N_1-isomer), 4.04 (1 H, app. t, *J* = 2.8 Hz, H_2 N_1-isomer), 4.00 (1 H, app. t, *J* = 2.6 Hz, H_2 N_2-isomer), 3.70 (1 H, dd, *J* = 8.9, 2.9 Hz, H_3 N_1 and N_2-isomers), 0.63 (9 H, s, $C(CH_3)_3$), 0.51 (9 H, s, $C(CH_3)_3$), -0.11 (3 H, s, $Si(CH_3)_2$), -0.17

(3 H, s, Si(CH_3)$_2$), -0.54 (3 H, s, Si(CH_3)$_2$), -0.68 (3 H, s, Si(CH_3)$_2$); **^{13}C NMR** (101 MHz; $CDCl_3$) 164.5 (C_q tetrazole, N_2-isomer), 152.3 (C_q tetrazole, N_1-isomer), 138.0 (C_q), 137.9 (C_q), 137.8 (C_q), 137.5 (C_q), 134.5 (C_q), 133.9 (C_q), 133.6 (C_q), 133.5 (C_q), 132.8, 131.8, 131.0, 129.2, 129.1, 129.0, 128.9, 128.8, 128.6, 128.5, 128.4, 128.3, 128.3, 128.3, 128.0, 127.9, 127.8, 127.7, 127.7, 127.6, 127.5, 86.5 (C1, N_1 or N_2-isomer), 86.4 (C1, N_1 or N_2-isomer), 80.2 (C3, N_1 or N_2-isomer), 80.1 (C3, N_1 or N_2-isomer), 76.3 (C2, N_1-isomer), 75.7 (C2, N_2-isomer), 72.9 (*C*H_2Ph), 72.1 (2C, *C*H_2Ph), 71.6 (*C*H_2Ph), 70.4 (C4, N_2-isomer), 69.9 (C4, N_1-isomer), 69.1 (C5, N_2-isomer), 67.8 (C5, N_1-isomer), 56.9 (*C*H_2Ph tetrazole, N_2-isomer), 51.2 (*C*H_2Ph tetrazole, N_1-isomer), 25.6 (C(CH_3)$_3$), 25.5 (C(CH_3)$_3$), 17.8 (*C*(CH_3)$_3$), 17.7 (*C*(CH_3)$_3$), -4.2 (Si(CH_3)$_2$), -4.5 (Si(CH_3)$_2$), -5.9 (Si(CH_3)$_2$), -6.0 (Si(CH_3)$_2$); **^{13}C-GATED** (101 MHz; $CDCl_3$): 86.5 and 86.4 ($^1J_{C1\text{-}H1}$ = 168 Hz, C1); **HRMS** (ES^+) *m/z* [Found: $(M+H)^+$ 695.3084 $C_{39}H_{47}N_4O_4SSi$ requires $(M+H)^+$, 695.3082].

S2.10. C-6 nitrile thioglycoside 16

S2.10.1. C-6 aldehyde thioglycoside intermediate

To a stirred solution of **15**[2] (420 mg, 0.77 mmol, 1.0 equiv) in dimethyl sulfoxide (7.7 mL) was added Et_3N (323 µL, d = 0.726, 2.32 mmol, 3.0 equiv) and sulfur trioxide pyridine complex (369 mg, 2.32 mmol, 3.0 equiv) at room temperature. The reaction mixture was left stirring for 1 h before it was diluted with EtOAc (25 mL) and H_2O (20 mL). The whole was extracted with EtOAc (3 × 20 mL) and the extracts were washed with H_2O (5 × 30 mL) and brine (2 × 30 mL). The organic layer was dried over Na_2SO_4, filtered and concentrated under reduced pressure. The crude aldehyde was obtained as a yellow oil (400 mg, 0.74 mmol, 96%) and was carried on the next step without further purification. R_f 0.83 (EtOAc/hexane, 1/2); $[\alpha]_D^{22}$ +40.5 (*c*. 1.0, $CHCl_3$); **^{1}H NMR** (400 MHz; $CDCl_3$) δ 9.73 (1 H, s, C*H*O), 7.49 – 7.27 (20 H, m, Ar-H), 5.59 (1 H, t, *J* = 6.3 Hz, H_1), 4.69 (1 H, d, *J* = 12.9 Hz, CH_2Ph), 4.63 (1 H, d, *J* = 12.8 Hz, CH_2Ph), 4.59 (2 H, d, *J* = 12.0 Hz, CH_2Ph), 4.55 (1 H, d, *J* = 11.9 Hz, CH_2Ph), 4.54 (1 H, d, *J* = 12.5 Hz, CH_2Ph), 4.49 (1 H, d, *J* = 7.7 Hz, H_5), 4.08 (1 H, app. t, *J* = 7.7 Hz, H_4), 3.94 – 3.91 (1 H, m, H_2), 3.87 (1 H, dd, *J* = 7.6, 2.8 Hz, H_3); **^{13}C NMR** (101 MHz; $CDCl_3$) δ 197.6 (*C*HO), 137.6 (C_q), 137.6 (C_q), 137.6 (C_q), 133.5 (C_q), 131.6, 129.1, 128.5, 128.4, 128.0, 128.0, 128.0, 127.9, 127.9, 127.9, 127.6, 84.9 (C1), 77.4 (C3), 77.2 (C5), 75.0 (C2), 74.7 (C4), 74.3 (*C*H_2Ph), 72.3 (*C*H_2Ph), 72.2 (*C*H_2Ph). These data were consistent with literature values.[2]

S2.10.2. C-6 oxime thioglycoside intermediate

The crude aldehyde from the previous step (4.72 g, 8.73 mmol, 1.0 equiv) was dissolved in THF (873 mL) and a solution of $H_2NOH.HCl$ (606 mg, 8.73 mmol, 1.0 equiv) dissolved in H_2O (17.5 mL) was added dropwise. The mixture was cooled to 0 °C and a solution of Na_2CO_3 (1.1 g, 10.5 mmol, 1.2 equiv) dissolved in H_2O (10.5 mL) was added dropwise. The solution was slowly warmed to room temperature and stirred for 24 h. The mixture was diluted with H_2O (200 mL) and then extracted with EtOAc (4 × 400 mL). The

organic layers were combined, dried over Na_2SO_4, filtered and concentrated under reduced pressure. The obtained crude oil was purified using silica gel flash column chromatography, eluting with Et_2O/petroleum ether (0/100, 5/95, 10/90, 20/80) to furnish the title compound as a colourless oil. *Cis* and *trans* (1/7) isomers were isolated separately (major isomer: 3.8 g, 6.54 mmol, 78%, minor isomer: 550 mg, 0.99 mmol, 11%) and both were used for the next step. Major isomer R_f 0.70; minor isomer R_f 0.62 (EtOAc/petroleum ether, 1/2); major: $[\alpha]_D^{22}$ +87.7 (*c*. 3.1, $CHCl_3$); **^{1}H NMR** (400 MHz; $CDCl_3$) major isomer δ 7.46 (1 H, d, *J* = 6.5 Hz, *H*C=N), 7.47 – 7.25 (20 H, m, Ar-H), 5.49 (1 H, d, *J* = 1.5 Hz, H_1), 4.86 (1 H, d, *J* = 10.9 Hz, CH_2Ph), 4.73 (1 H, dd, *J* = 10.5, 5.6 Hz, H_5), 4.68 (1 H, d, *J* = 12.4 Hz, CH_2Ph), 4.66 (1 H, d, *J* = 11.7 Hz, CH_2Ph), 4.65 (1 H, d, *J* = 10.7 Hz, CH_2Ph), 4.64 (1 H, d, *J* = 12.3 Hz, CH_2Ph), 4.61 (1 H, d, *J* = 11.7 Hz, CH_2Ph), 4.01 (1 H, app. t, *J* = 9.4 Hz, H_4), 4.01– 3.98 (1 H, m, H_2), 3.87 (1 H, dd, *J* = 9.2, 2.9 Hz, H_3); **^{13}C NMR** (101 MHz; $CDCl_3$) δ 148.7 (H*C*=N), 138.1 (C_q), 137.7 (C_q), 133.9 (C_q), 131.7 (C_q), 129.1, 128.4, 128.4, 128.3, 128.5, 128.0, 127.8, 127.8, 127.8, 127.6, 86.1 (C1), 79.5 (C3), 76.4 (1 C, C2 or C4), 76.3 (1 C, C2 or C4), 75.1 (*C*H_2Ph), 72.4 (*C*H_2Ph), 72.3 (*C*H_2Ph), 70.6 (C5); **HRMS** (ES^+) *m/z* [Found: $(M+Na)^+$ 578.1993 $C_{33}H_{33}NO_5SNa$ requires $(M+Na)^+$, 578.1977].

S2.10.3 C-6 nitrile thioglycoside 16

The previously synthesised oxime (4.35 g, 7.83 mmol, 1.0 equiv) was dissolved in dry MeCN (783 mL) and $POCl_3$ (729 µL, d = 1.645, 7.83 mmol, 1.0 equiv) was added at room temperature. The solution was stirred for 5 min. at room temperature, heated up to 65 °C and then stirred for 3 h. The reaction was quenched with sat. aq. $NaHCO_3$ solution (20 mL) and extracted with EtOAc (3 × 300 mL). The organic layers were combined, dried over Na_2SO_4, filtered and concentrated under reduced pressure. The crude product was purified using silica gel flash column chromatography, eluting with EtOAc/petroleum ether (0/100, 5/95, 10/90, 20/80) to furnish **16** as a yellow oil (2.5 g, 4.65 mmol, 59%). R_f 0.76 (EtOAc/hexane, 1/2); $[\alpha]_D^{22}$ +71.0 (*c*. 0.93, $CHCl_3$); **^{1}H NMR** (400 MHz; $CDCl_3$) δ 7.38 – 7.28 (20 H, m, Ar-H), 5.48 (1 H, d, *J* = 2.2 Hz, H_1), 4.88 (1 H, d, *J* = 12.9 Hz, CH_2Ph), 4.88 (1H, d, *J* = 9.7 Hz, H_5), 4.68 (1 H, d, *J* = 12.1 Hz, CH_2Ph), 4.66 (2 H, d, *J* = 11.6 Hz, CH_2Ph), 4.63 (1 H, d, *J* = 11.9 Hz, CH_2Ph), 4.60 (1 H, d, *J* = 11.7 Hz, CH_2Ph), 4.19 (1 H, app.t, *J* = 9.2 Hz, H_4), 3.93 (1 H, app. t, *J* = 2.6 Hz, H_2), 3.71 (1 H, dd, *J* = 8.9, 2.9 Hz, H_3); **^{13}C NMR** (101 MHz; $CDCl_3$) δ 137.5 (C_q), 137.3 (C_q), 137.2 (C_q), 132.7 (C_q), 131.5 (C_q), 129.3, 128.5, 128.5, 128.5, 128.4, 128.2, 128.1, 128.0, 128.0, 127.9, 117.1 (*C*≡N), 86.2 (C1), 78.4 (C3), 76.1 (C4), 75.8 (C2 or *C*H_2Ph), 75.7 (C2 or *C*H_2Ph), 72.7 (*C*H_2Ph), 72.6 (*C*H_2Ph), 62.2 (C5); **HRMS** (ES^+) *m/z* [Found: $(M+H)^+$ 538.2068 $C_{33}H_{33}NO_4S$ requires $(M+H)^+$, 358.2052].

S2.11. Compound 17

C6-nitrile thioglycoside **16** (2.5 g, 4.65 mmol, 1.0 equiv) was dissolved in toluene (465 mL) and $TMSN_3$ (3.7 mL, d = 0.872, 27.9 mmol, 6.0 equiv) and Bu_2SnO (463 mg, 1.86 mmol, 0.4 equiv) were added. The mixture was heated to 120 °C and stirred for 16 h. Upon completion, the mixture was cooled down to room temperature, diluted with EtOAc (400 mL) and washed with 0.1 M aq. HCl (250 mL). The organic layer was dried over Na_2SO_4, filtered and concentrated under reduced pressure to afford the crude product. Purification of the crude material by silica gel flash column chromatography, eluting with MeOH/CH_2Cl_2 (0/100, 2/98, 5/95) afforded **17** as a brown oil (1.5 g, 2.58 mmol, 55%). R_f 0.65 (MeOH/CH_2Cl_2, 1/9); $[\alpha]_D^{22}$ +95.0 (*c*. 1.96, $CHCl_3$); **^{1}H NMR** (400 MHz; $CDCl_3$) δ 7.37 – 7.08 (20 H, m, Ar-H), 5.61 (1 H, d, *J* = 9.7 Hz, H_5), 5.54 (1 H, d, *J* = 1.7 Hz, H_1), 4.71 (2 H, d, *J* = 11.9 Hz, CH_2Ph), 4.70 (1 H, d, *J* = 10.5 Hz, CH_2Ph), 4.65 (2 H, d, *J* = 11.8 Hz, CH_2Ph), 4.38 (1 H, d, *J* = 10.7 Hz, CH_2Ph), 4.19 (1 H, app. t, *J* = 9.4 Hz, H_4), 4.08 (1 H, dd, *J* = 2.7, 2.1 Hz, H_2), 3.99 (1 H, dd, *J* = 9.2, 2.9 Hz, H_3); **^{13}C NMR** (101 MHz; $CDCl_3$) δ 155.0 (C_q tetrazole), 137.6 (C_q), 137.1 (C_q), 137.0 (C_q), 132.8 (C_q), 132.0, 129.3, 128.7, 128.6, 128.6, 128.4, 128.4, 128.4, 128.1, 128.1, 128.0, 127.9, 86.8 (C1), 79.4 (C3), 76.6 (C2 and C4), 75.0 (CH_2Ph), 73.3 (CH_2Ph), 72.8 (CH_2Ph), 66.5 (C5); **HRMS** (ES^+) *m/z* [Found: $(M+H)^+$ 581.2251 $C_{33}H_{34}N_4O_4S$ requires $(M+H)^+$, 581.2223].

S2.12. PMB-protected C-6 tetrazole thioglycosides 18 and 19

To a stirred solution of **17** (920 mg, 1.37 mmol, 1.0 equiv) in DMF (10 mL) was added successively, KI (341 mg, 2.06 mmol, 1.5 equiv), K_2CO_3 (227 mg, 1.65 mmol, 1.2 equiv) and PMBCl (279 µL, d = 1.155, 2.06 mmol, 1.5 equiv). The reaction was left stirring for 16 h and was diluted with CH_2Cl_2 (30 mL). The organic layer was washed with 10% aq. $Na_2S_2O_3$ solution (30 mL) and brine (30 mL), dried over $MgSO_4$, filtered and concentrated under reduced pressure. The crude product was purified using silica gel flash column chromatography, eluting with EtOAc/petroleum ether (5/95, 10/90, 15/85) to furnish isomers **18** and **19** (732 mg, 1.04 mmol, 76%) as colourless oils.

S2.12.1. Compound 18

N_1-regioisomer **18** was isolated as a yellow oil (374 mg, 0.53 mmol, 39%). R_f 0.72 (EtOAc/petroleum ether, 1/2); $[\alpha]_D^{22}$ +48.5 (*c*. 2.75, $CHCl_3$); **^{1}H NMR** (400 MHz; $CDCl_3$) δ 7.39 – 6.95 (21 H, m, Ar-H), 7.00 (2 H, d, *J* = 8.8 Hz, Ar-H PMB), 6.65 (2 H, d, *J* = 8.8 Hz, Ar-H PMB), 5.61 (1 H, d, *J* = 1.6 Hz, H_1), 5.45 (1 H, d, *J* = 9.9 Hz, H_5), 5.28 (1 H, d, *J* = 15.0 Hz, CH_2Ph-PMB), 5.19 (1 H, d, *J* = 15.0 Hz, CH_2Ph-PMB), 4.72 (2 H, d, *J* = 12.5 Hz, CH_2Ph), 4.70 (1 H, d, *J* = 11.7 Hz, CH_2Ph), 4.67 (1 H, d, *J* = 11.0 Hz, CH_2Ph), 4.65 (1 H, d, *J* = 12.3 Hz, CH_2Ph), 4.45 (1 H, app. t, *J* = 9.6 Hz, H_4), 4.33 (1 H, d, *J* = 10.6 Hz, CH_2Ph), 4.08 (1 H, dd, *J* = 2.7, 2.0 Hz, H_2), 3.93 (1 H, dd, *J* = 9.2, 2.8 Hz, H_3), 3.70 (3 H, s, OCH_3); **^{13}C NMR** (101 MHz; $CDCl_3$) δ 159.6 (C_q PMB), 152.2 (C_q tetrazole), 137.8 (C_q),

137.5 (C_q), 137.5 (C_q), 133.3 (C_q), 130.7 (C_q), 129.3, 129.3, 128.5, 128.3, 128.2, 128.1, 128.0, 127.9, 127.8, 127.8, 127.7, 125.6, 114.1, 86.3 (C1), 79.7 (C3), 76.4 (C2), 76.0 (C4), 75.1 (*C*H_2Ph), 72.8 (*C*H_2Ph), 72.5 (*C*H_2Ph), 65.6 (C5), 55.2 (O*C*H_3), 50.5 (*C*H_2Ph PMB); **^{13}C-GATED** (101 MHz; $CDCl_3$): 86.3 ($^1J_{C1\text{-}H1}$ =172 Hz, C1); **HRMS** (ES^+) *m/z* [Found: $(M+H)^+$ 701.2829 $C_{41}H_{42}N_4O_4S$ requires $(M+H)^+$, 701.2798].

S2.12.2. Compound 19

N_2-regioisomer **19** was isolated as a yellow oil (355 mg, 0.51 mmol, 37%). R_f 0.73 (EtOAc/petroleum ether, 1/2); **^{1}H NMR** (400 MHz; $CDCl_3$) δ 7.38 – 7.13 (21 H, m, Ar-H), 6.84 (2 H, d, *J* = 8.0 Hz, Ar-H PMB), 6.76 (2 H, d, *J* = 8.7 Hz, Ar-H PMB), 5.57 (1 H, d, *J* = 1.3 Hz, H_1), 5.52 (1 H, d, *J* = 9.9 Hz, H_5), 5.27 (1 H, d, *J* = 15.0 Hz, C*H_2*Ph-PMB), 5.18 (1 H, d, *J* = 15.0 Hz, C*H_2*Ph-PMB), 4.63 (1 H, d, *J* = 11.5 Hz, C*H_2*Ph), 4.63 (1 H, d, *J* = 11.6 Hz, C*H_2*Ph), 4.59 (1 H, d, *J* = 11.9 Hz, C*H_2*Ph), 4.58 (1 H, app. t, *J* = 9.9 Hz, H_4), 4.56 (1 H, d, *J* = 11.5 Hz, C*H_2*Ph), 4.08 – 4.06 (1 H, m, H_2), 3.94 (1 H, dd, *J* = 9.3, 1.2 Hz, H_3), 4.33 (1 H, d, *J* = 10.7 Hz, C*H_2*Ph), 4.23 (1 H, d, *J* = 10.8 Hz, C*H_2*Ph), 3.67 (1 H, s, OC*H_3*); **^{13}C NMR** (101 MHz; $CDCl_3$) δ 164.3 (C_q tetrazole), 160.0 (C_q PMB), 138.1 (C_q), 138.1 (C_q), 137.8 (C_q), 133.8 (C_q), 132.0 (C_q), 129.4, 129.3, 128.6, 128.5, 128.3, 128.1, 128.1, 127.9, 127.8, 127.7, 125.3, 114.3, 86.6 (C1), 79.8 (C3), 76.3 (C2), 76.0 (C4), 75.1 (*C*H_2Ph), 72.4 (*C*H_2Ph), 72.2 (*C*H_2Ph), 67.1 (C5), 56.5 (*C*H_2Ph PMB), 55.3 (O*C*H_3); **^{13}C-GATED** (101 MHz; $CDCl_3$): 86.6 ($^1J_{C1\text{-}H1}$ =168 Hz, C1); **HRMS** (ES^+) *m/z* [Found: $(M+H)^+$ 701.2829 $C_{41}H_{42}N_4O_4S$ requires $(M+H)^+$, 701.2798].

S2.13. Compound 20

S2.13.1. Protected aminopropyl derivative

A solution of **18** and **19** (290 mg, 0.41 mmol, 1.0 equiv) and 3-(benzyloxycarbonylamino)-1-propanol (259 mg, 1.24 mmol, 3.0 equiv) in in CH_2Cl_2 (4.1 mL) was stirred over activated MS 4Å for 1 h before NIS (139 mg, 0.62 mmol, 1.5 equiv) was added. The mixture was cooled to −40 °C before AgOTf (53 mg, 0.21 mmol, 0.5 equiv) was added. The reaction was warmed up to 0 °C and stirred for 3 h. Upon completion, Et_3N was added until pH = 7, and subsequently diluted with CH_2Cl_2 (20 mL). The organic layer was washed with 10% aq. $Na_2S_2O_3$ solution (20 mL), brine (20 mL), dried over $MgSO_4$, filtered and concentrated under reduced pressure. Flash column chromatography, eluting with EtOAc/petroleum (30/70, 40/60 and 50/50) afforded the title compound as a colourless oil in an anomeric mixture of α/β = 3/1 ratio (110 mg, 0.14 mmol, 34%). R_f 0.66 (EtOAc/toluene, 3/7); **^{1}H NMR** (400 MHz; $CDCl_3$) 7.40 – 7.22 (23 H, m, Ar-H), 7.19 – 7.09 (6 H, m, Ar-H PMB), 6.83 – 6.73 (4 H, m, Ar-H PMB), 5.65 (1 H, d, *J* = 14.4 Hz, C*H_2*Ph PMB), 5.60 (1 H, d, *J* = 14.6 Hz, C*H_2*Ph PMB), 5.60 (1 H, d, *J* = 14.6 Hz, C*H_2*Ph PMB), 5.55 (1 H, d, *J* = 14.4 Hz, C*H_2*Ph PMB), 5.09 (1 H, d, *J* = 12.0 Hz, C*H_2*Ph), 5.03 (1 H, d, *J* = 12.4 Hz, C*H_2*Ph), 4.96 (1 H, d, *J* = 12.6 Hz, C*H_2*Ph), 4.92 (1 H, d, *J* = 9.9 Hz, H_5 α-anomer), 4.85 (1 H, d, *J* = 2.2 Hz, H_1 α-anomer), 4.83 (1 H, d, *J* = 12.6

Hz, CH_2Ph), 4.78 (1 H, d, J = 12.5 Hz, CH_2Ph), 4.73 (1 H, d, J = 11.2 Hz, CH_2Ph), 4.69 (2 H, d, J = 10.7 Hz, CH_2Ph), 4.63 (2 H, d, J = 11.8 Hz, CH_2Ph), 4.62 (1 H, d, J = 9.6 Hz, H_5 β-anomer), 4.59 (1 H, d, J = 11.7 Hz, CH_2Ph), 4.55 (1 H, d, J = 3.0 Hz, H_1 β-anomer), 4.53 (1 H, d, J = 12.8 Hz, CH_2Ph), 4.49 (2 H, app. t, J = 9.7 Hz, H_4 α and β-anomer), 4.47 (1 H, d, J = 12.8 Hz, CH_2Ph), 4.28 (1 H, d, J = 10.7 Hz, CH_2Ph), 4.24 (1 H, d, J = 10.7 Hz, CH_2Ph), 3.96 (1 H, d, J = 2.7 Hz, H_2 β-anomer), 3.95 (1 H, dd, J = 9.4, 2.9 Hz, H_3 α-anomer), 3.93 – 3.88 (1 H, m, $OCH_2CH_2CH_2NHCbz$ α-anomer), 3.90 – 3.79 (2 H, m, H_2 α-anomer, $OCH_2CH_2CH_2NHCbz$ β-anomer), 3.72 (3 H, s, OCH_3 α-anomer), 3.70 (3 H, s, OCH_3 β-anomer), 3.59 (1 H, dd, J = 9.4, 2.7 Hz, H_3 β-anomer), 3.56 – 3.50 (1 H, m, $OCH_2CH_2CH_2NHCbz$ α-anomer), 3.47 – 3.40 (1 H, m, $OCH_2CH_2CH_2NHCbz$ β-anomer), 3.28 (4 H, m, $OCH_2CH_2CH_2NHCbz$ α and β-anomer), 1.78 (4 H, dt, J = 12.9, 6.9 Hz, $OCH_2CH_2CH_2NHCbz$ α and β-anomer); **^{13}C NMR** (101 MHz; $CDCl_3$) δ 164.5 (C_q tetrazole α-anomer), 164.0 (C_q tetrazole β-anomer), 160.0 (C_q PMB), 156.4 (C=O CBz), 138.6 (C_q), 138.4 (2 C, C_q), 138.2 (2 C, C_q), 138.1 (C_q), 138.1 (C_q), 136.6 (C_q), 130.0 (C_q), 130.0 (C_q), 129.1, 128.5, 128.5, 128.4, 128.4, 128.1, 128.1, 128.0, 127.7, 127.7, 127.7, 127.6, 127.6, 127.4, 114.3, 102.2 (C1 β-anomer), 98.9 (C1 α-anomer), 81.8 (C3 β-anomer), 79.9 (C3 α-anomer), 77.2 (2 C, C4, α and β-anomer), 75.0 (C2 α-anomer), 74.8 (CH_2Ph), 74.2 (CH_2Ph), 74.2 (C2 β-anomer), 74.1 (CH_2Ph), 72.9 (CH_2Ph), 72.5 (2 C, CH_2Ph), 71.7 (CH_2Ph), 69.8 (C5 β-anomer), 67.6 ($OCH_2CH_2CH_2NHCbz$ α-anomer), 66.6 (CH_2Ph), 66.4 (C5 α-anomer), 65.8 ($OCH_2CH_2CH_2NHCbz$ β-anomer), 56.4 (2 C, CH_2Ph PMB), 55.2 (2 C, OCH_3), 38.4 ($OCH_2CH_2CH_2NHCbz$ α-anomer), 38.2 ($OCH_2CH_2CH_2NHCbz$ β-anomer), 29.7 ($OCH_2CH_2CH_2NHCbz$ β-anomer), 29.5 ($OCH_2CH_2CH_2NHCbz$ α-anomer); **^{13}C-GATED** (101 MHz; $CDCl_3$): 102.2 ($^1J_{C1-H1}$ =156 Hz, C1 β-anomer); **HRMS** (ES^+) *m/z* [Found: $(M+H)^+$ 800.3693 $C_{46}H_{51}N_5O_8$ requires $(M+H)^+$, 800.3659].

S2.13.2. Compound 20

Protected aminopropyl derivative (30 mg, 38 μmol, 1.0 equiv) was dissolved in a mixture of EtOH/THF (0.6 mL, 1.5/1 *v/v*), after which Pd/C (10%) (20 mg, 19 μmol, 0.5 equiv), $Pd(OH)_2/C$ (20%) (13 mg, 19 μmol, 0.5 equiv) and 0.1 M aq. HCl (380 μL, 38 μmol, 1.0 equiv) were added. The mixture was stirred for 56 h under an atmosphere of hydrogen (1 atm, balloon) at room temperature. TLC analysis (hexane/EtOAc, 1/2) showed complete conversion of starting material to a lower R_f spot. The reaction mixture was filtered through Celite®, followed by solvent removal *in vacuo* to give white powder **20** in an anomeric mixture of α/β = 3/1 (11 mg, 36 μmol, 96%). R_f 0.27 (H_2O/MeCN, 1/2); **^{1}H NMR** (400 MHz; D_2O) δ 4.86 (1 H, s, H_1 α-anomer), 4.82 (1 H, d, J = 9.8 Hz, H_5), 4.74 (1 H, s, H_1 β-anomer), 4.61 (1 H, d, J = 9.9 Hz, H_5 β-anomer), 4.15 (1 H, app. t, J = 9.9 Hz, H_4 α-anomer), 4.08 (app. t, J = 9.9 Hz, H_4 β-anomer), 4.03 (1 H, d, J = 3.2 Hz, H_2 β-anomer), 4.01 – 3.98 (1 H, m, H_2 α-anomer), 3.87 (1 H, dd, J = 9.8, 3.4 Hz, H_3 α-anomer), 3.84 – 3.76 (2 H, m, $OCH_2CH_2CH_2NH_3.Cl$ α and β-anomer), 3.73 (1 H, dd, J = 9.8, 3.2 Hz, H_3 β-anomer), 3.56 (2 H, ddd, J = 17.3, 9.7, 4.5 Hz, $OCH_2CH_2CH_2NH_3.Cl$

α and β-anomer), 3.17 – 3.07 (2 H, m, $OCH_2CH_2CH_2NH_3$.Cl α-anomer), 3.02 (2 H, td, *J* = 12.6, 7.2 Hz, $OCH_2CH_2CH_2NH_3$.Cl β-anomer), 1.99 (2 H, dq, *J* = 13.6, 6.7 Hz, $OCH_2CH_2CH_2NH_3$.Cl α-anomer), 1.91 – 1.82 (2 H, m, $OCH_2CH_2CH_2NH_3$.Cl β-anomer); **^{13}C NMR** (101 MHz; D_2O) δ 160.1 (2 C, C_q tetrazole), 100.6 (C1 β-anomer), 100.5 (C1 α-anomer), 72.7 (C3 β-anomer), 70.5 (C3 α-anomer), 70.5 (C2 β-anomer), 70.2 (C5 β-anomer), 70.0 (C2 α-anomer), 69.6 (C4 β-anomer), 69.5 (C4 α-anomer), 67.6 ($OCH_2CH_2CH_2NH_3$.Cl β-anomer), 66.6 (C5 α-anomer), 65.3 ($OCH_2CH_2CH_2NH_3$.Cl α-anomer), 37.6 ($OCH_2CH_2CH_2NH_3$.Cl β-anomer), 37.4 ($OCH_2CH_2CH_2NH_3$.Cl α-anomer), 26.7 ($OCH_2CH_2CH_2NH_3$.Cl α-anomer), 26.6 ($OCH_2CH_2CH_2NH_3$.Cl β-anomer); **^{13}C-GATED** (101 MHz; D_2O): 100.5 ($^1J_{C1-H1}$ = 172 Hz, C1 α-anomer); **HRMS** (ES^+) *m/z* [Found: $(M+H)^+$ 276.1309 $C_9H_{19}N_5O_5$ requires $(M+H)^+$, 276.1308].

S2.14. Compound 21

S2.14.1. Fully protected C-6 tetrazole 1-phosphates

A mixture of **18** and **19** (730 mg, 1.04 mmol, 1.0 equiv) was stirred with activated MS4Å for 1h in CH_2Cl_2 (10 mL). Dibenzyl phosphate (580 mg, 2.08 mmol, 2.0 equiv) was added, and the solution was stirred for further 30 min. before being cooled down to -30 °C. NIS (350 mg, 1.56 mmol, 1.5 equiv) and AgOTf (133 mg, 0.52 mmol, 0.5 equiv) were added successively and the reaction mixture was stirred for further 3.5 h, allowing the temperature to reach 0 °C. When TLC analysis indicated conversion to a lower R_f value, the reaction was quenched by the addition of Et_3N (1.4 mL, d = 0.726, 10.4 mmol, 10.0 equiv) and diluted with CH_2Cl_2 (50 mL). The organic layer was washed with 10% aq. $Na_2S_2O_3$ solution (30 mL), brine (30 mL), dried over $MgSO_4$, filtered and concentrated under reduced pressure. Flash column chromatography, eluting with EtOAc/toluene (5/95, 10/90 and 30/70) afforded the protected 1-phosphate regiosiomeric mixture as a colourless oil in a 50/50 ratio (650 mg, 0.75 mmol, 72%). R_f 0.66 (EtOAc/toluene, 3/7); **^{1}H NMR** (400 MHz; $CDCl_3$) δ 7.38 – 7.27 (35 H, m, Ar-H), 7.14 (2 H, d, *J* = 7.2 Hz, Ar-H PMB), 7.07 (2 H, d, *J* = 8.7 Hz, Ar-H PMB), 6.93 (2 H, d, *J* = 6.7 Hz, Ar-H), 6.82 (2 H, d, *J* = 6.5 Hz, Ar-H), 6.75 (2 H, d, *J* = 8.8 Hz, Ar-H PMB), 6.65 (2 H, d, *J* = 8.8 Hz, Ar-H PMB), 5.76 (1 H, dd, *J* = 6.3, 1.8 Hz, H_1 N_2-isomer), 5.69 (1 H, dd, *J* = 6.3, 2.1 Hz, H_1 N_1-isomer), 5.65 (1 H, d, *J* = 14.0 Hz, CH_2Ph PMB), 5.61 (1 H, d, *J* = 14.2 Hz, CH_2Ph PMB), 5.33 (1 H, d, *J* = 15.0 Hz, CH_2Ph PMB), 5.16 (1 H, d, *J* = 13.4 Hz, CH_2Ph PMB), 5.15 (1 H, d, *J* = 10.0 Hz, H_5 N_1-isomer or N_2-isomer), 5.12 (1 H, d, *J* = 9.7 Hz, H_5 N_1-isomer or N_2-isomer), 5.04 (2 H, d, *J* = 8.6 Hz, OP(O)OCH_2Ph), 4.96 (2 H, d, *J* = 8.3 Hz, OP(O)OCH_2Ph), 4.95 (2 H, d, *J* = 8.3 Hz, OP(O)OCH_2Ph), 4.94 (2 H, d, *J* = 8.3 Hz, OP(O)OCH_2Ph), 4.72 (1 H, d, *J* = 11.9 Hz, CH_2Ph), 4.71 (1 H, d, *J* = 11.0 Hz, CH_2Ph), 4.69 (1 H, d, *J* = 11.2 Hz, CH_2Ph), 4.64 (1 H, d, *J* = 11.8 Hz, CH_2Ph), 4.64 (1 H, d, *J* = 10.1 Hz, CH_2Ph), 4.60 (1 H, d, *J* = 11.8 Hz, CH_2Ph), 4.57 (1 H, d, *J* = 10.6 Hz, CH_2Ph), 4.54 (2 H, d, *J* = 11.7 Hz, CH_2Ph), 4.53 (1 H, app. t, *J* = 9.6 Hz, H_4 N_2-isomer), 4.49 (1 H, d, *J* = 11.6 Hz, CH_2Ph), 4.39 (1 H, app. t, *J* = 9.8 Hz, H_4 N_1-isomer), 4.29 (1 H, d, *J* = 10.6

Hz, CH_2Ph), 4.21 (1 H, d, *J* = 10.7 Hz, CH_2Ph), 3.89 (1 H, dd, *J* = 9.6, 3.2 Hz, H_3 N_2-isomer), 3.86 (1 H, dd, *J* = 9.6, 3.0 Hz, H_3 N_1-isomer), 3.78 (1 H, dd, *J* = 4.8, 2.2 Hz, H_2 N_2-isomer), 3.74 – 3.72 (1 H, m, H_2 N_1-isomer), 3.71 (3 H, s, OCH_3), 3.70 (3 H, s, OCH_3); **^{13}C NMR** (101 MHz; $CDCl_3$) δ 163.8 (C_q tetrazole N_2-isomer), 160.0 (C_q PMB), 159.7 (C_q PMB), 151.5 (C_q tetrazole N_1-isomer), 138.1 (C_q), 138.0 (C_q), 137.9 (C_q), 137.7 (C_q), 137.4 (C_q), 137.4 (C_q), 135.6 (d, *J* = 6.7 Hz, C_q OP(O)OBn), 135.5 (d, *J* = 6.7 Hz, C_q OP(O)OBn), 135.2 (d, *J* = 6.2 Hz, C_q OP(O)OBn), 135.1 (d, *J* = 6.4 Hz, C_q OP(O)OBn), 130.0 (C_q), 129.6 (C_q), 128.9, 128.7, 128.7, 128.6, 128.6, 128.6, 128.5, 128.4, 128.4, 128.3, 128.2, 128.2, 128.2, 128.1, 128.1, 128.0, 128.0, 128.0, 127.9, 127.9, 127.8, 127.7, 127.7, 127.6, 127.6, 127.4, 125.8, 125.2, 114.3, 114.2, 96.2 (d, *J* = 6.1 Hz, C1 N_2-isomer), 96.1 (d, *J* = 6.1 Hz, C1 N_1-isomer), 78.6 (C3 N_1-isomer or N_2-isomer), 78.4 (C3 N_1-isomer or N_2-isomer), 76.4 (C4 N_2-isomer), 75.1 (2 C, *C*H_2Ph), 76.0 (C4 N_1-isomer), 74.5 (d, *J* = 9.6 Hz, C2 N_1-isomer), 74.3 (d, *J* = 9.3 Hz, C2 N_2-isomer), 73.5 (*C*H_2Ph), 72.9 (*C*H_2Ph), 72.6 (*C*H_2Ph), 72.5 (*C*H_2Ph), 70.1 (d, *J* = 5.6 Hz, OP(O)O*C*H_2Ph, 70.0 (d, *J* = 5.7 Hz, OP(O)O*C*H_2Ph), 69.7 (d, *J* = 5.4 Hz, OP(O)O*C*H_2Ph), 69.6 (d, *J* = 5.5 Hz, OP(O)O*C*H_2Ph), 67.9 (C5 N_2-isomer), 66.5 (C5 N_1-isomer), 56.5 (*C*H_2Ph PMB), 55.2 (2 C, O*C*H_3), 50.4 (*C*H_2Ph PMB); **^{31}P NMR** δ_P (162 MHz, $CDCl_3$) -2.88 (s), -2.79 (s); **HRMS** (ES^+) *m/z* [Found: $(M+H)^+$ 869.3370 $C_{49}H_{51}N_4O_9P$ requires $(M+H)^+$, 869.3315].

S2.14.2. Compound 21

A suspension of the protected 1-phosphate regiosiomeric mixture (190 mg, 0.22 mmol, 1.0 equiv), 10% Pd/C (140 mg, 0.13 mmol, 0.6 equiv), 20% $Pd(OH)_2$/C (92 mg, 0.13 mmol, 0.6 equiv) and 5% aq. $NaHCO_3$ (739 µL, 0.44 mmol, 2.0 equiv) in a mixture of EtOH/THF (4.4 mL, 1.5/1 *v/v*) was stirred under an atmosphere of hydrogen (1 atm, balloon) at room temperature for 24 h. TLC analysis (hexane/EtOAc, 1/2) showed complete conversion of starting material to a lower R_f spot. The reaction mixture was filtered through Celite®, followed by solvent removal *in vacuo*. Purification *via* strong anion exchange chromatography was conducted manually using a Bio-Scale™ Mini UNOsphere™ Q (strong anion exchange) cartridge) and lyophilisation afforded **21** as a white powder (53 mg, 0.16 mmol, 72%). R_f 0.42 (H_2O/MeCN, 1/2); $[\alpha]_D^{22}$ -3.0 (*c*. 1.0, H_2O); **^{1}H NMR** (400 MHz; D_2O) δ 5.41 (1 H, dd, *J* = 7.9, 1.7 Hz, H_1), 5.08 (1 H, d, *J* = 9.7 Hz, H_5), 4.05 (1 H, app. t, *J* = 9.6 Hz, H_4), 4.02 – 3.97 (2 H, m, H_2 and H_3); **^{13}C NMR** (101 MHz; D_2O) δ 160.8 (C_q tetrazole), 96.1 (C1), 70.5 (C2), 69.7 (C3), 69.4 (C4), 67.1 (C5); **^{31}P NMR** δ_P (162 MHz, D_2O) -2.15 (s); **HRMS** (ES^-) *m/z* [Found: $(M-H)^-$ 297.0236 $C_6H_{10}N_4O_8P$ requires $(M-H)^-$, 297.0233].

S3. HMBC spectrum for N_1-protected tetrazole 11

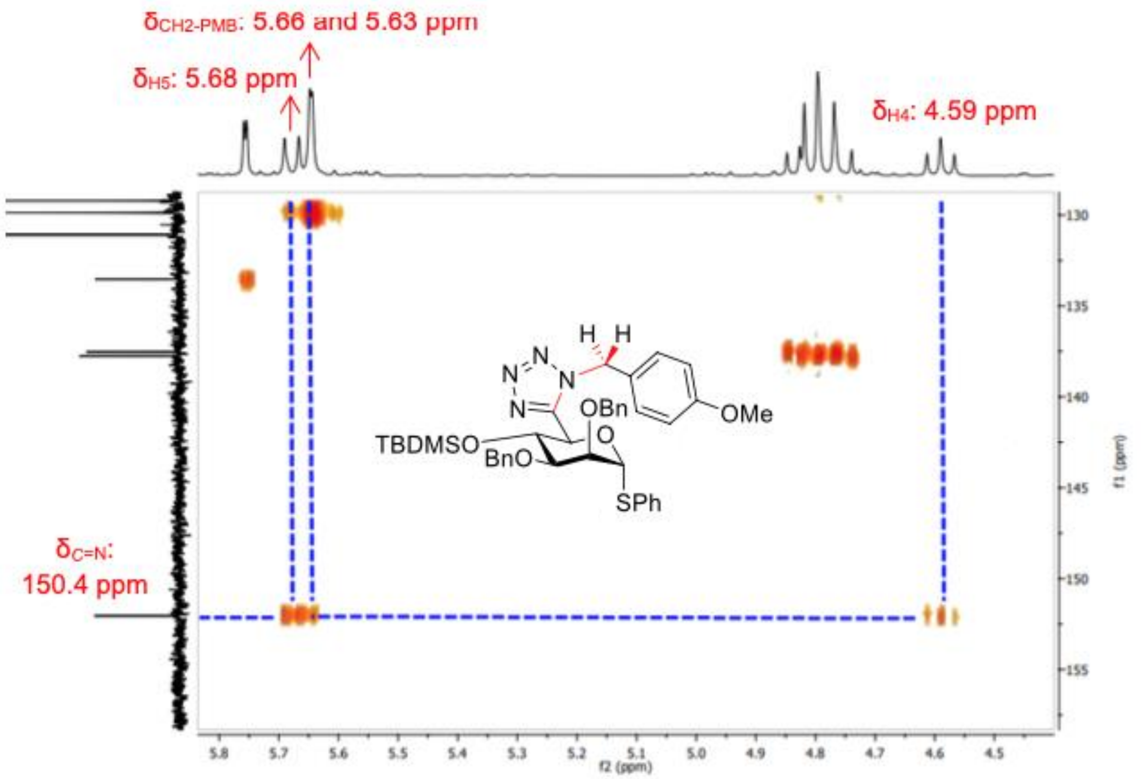

S4. References

(1) Dimitriou, E.; Miller, G. J. *Org. Biomol. Chem.* **2019**, *17*, 9321–9335.

(2) Ahmadipour, S.; Pergolizzi, G.; Rejzek, M.; Field, R. A.; Miller, G. J. *Org. Lett.* **2019**, *21*, 4415–4419.

S5. Spectral Data: ^{1}H, ^{13}C, ^{31}P and HSQC NMR for compounds 2-5, 7-14, 16-18 and 20-21

Compound 2

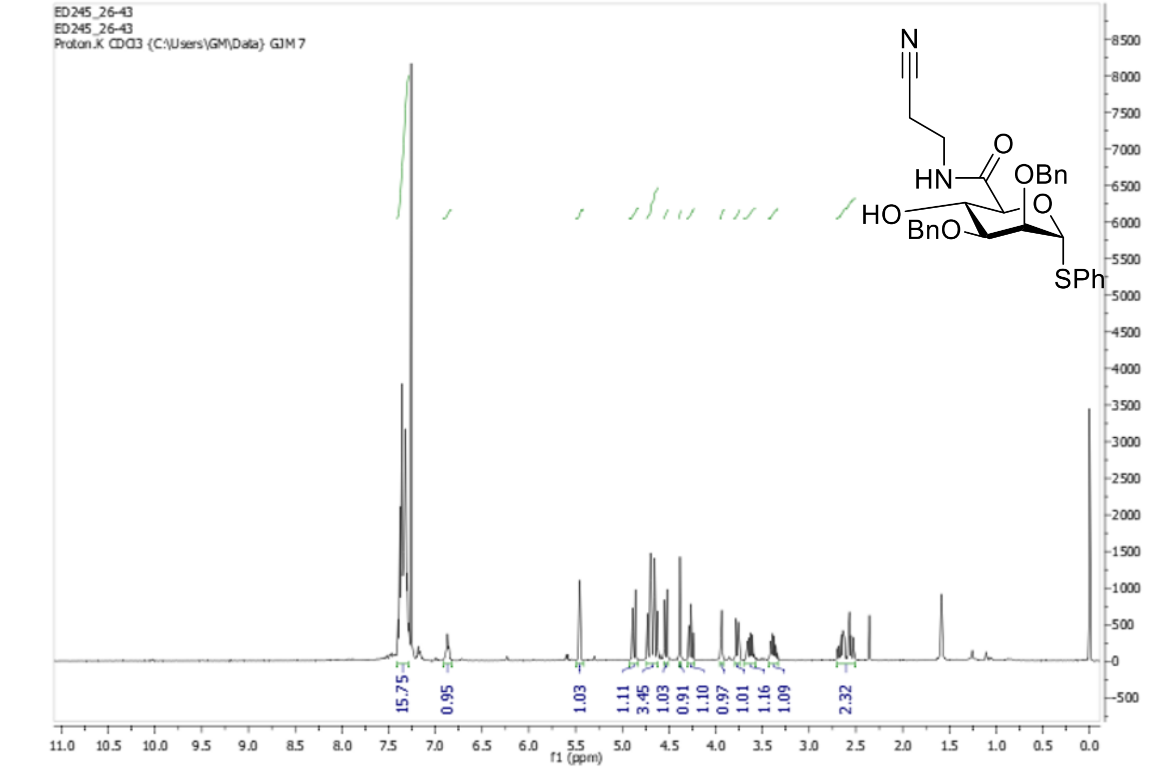
ED245_26-43
ED245_26-43
Proton.K CDCl3 {C:\Users\GM\Data} GJM 7
HN
O
OBn
HO
BnO
O
N
SPh
15.75
0.95
1.03
1.11
3.45
1.03
0.91
1.10
0.97
1.01
1.16
1.09
2.32
f1 (ppm)

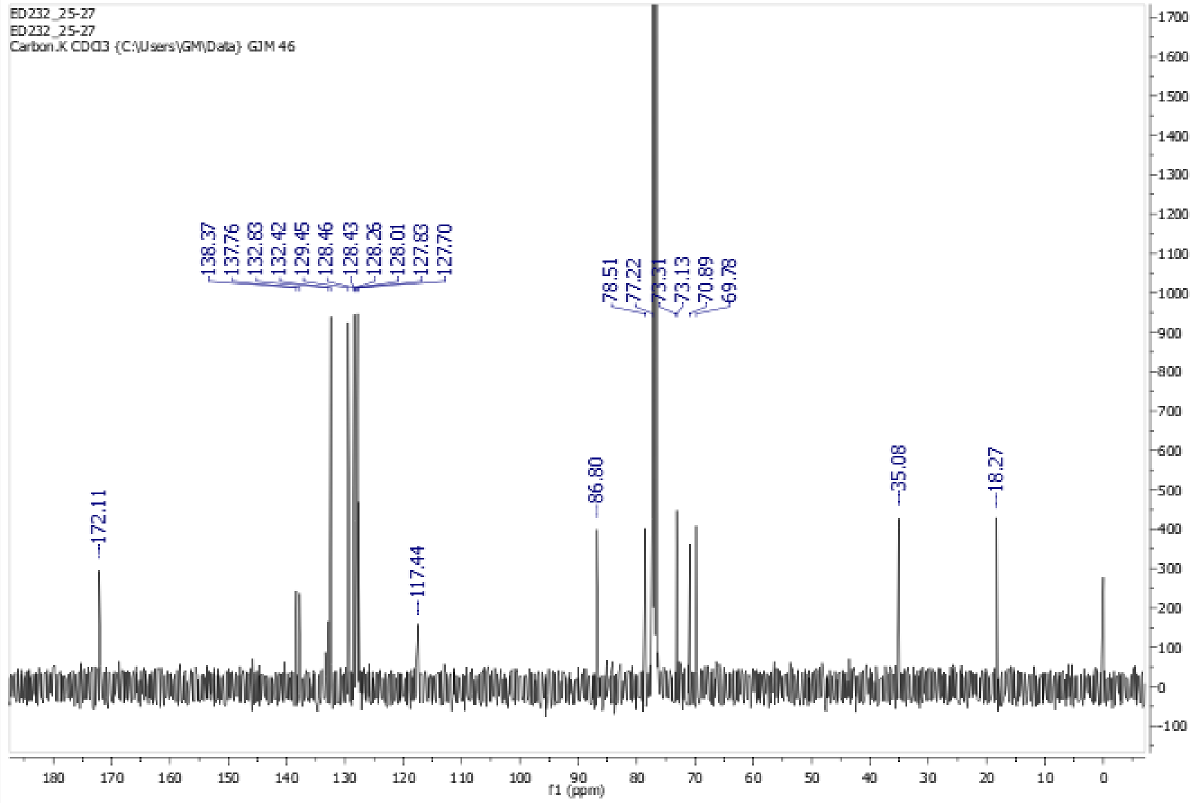
ED232_25-27
ED232_25-27
Carbon.K CDCl3 {C:\Users\GM\Data} GJM 46
172.11
138.37
137.76
132.83
132.42
129.45
128.46
128.43
128.26
128.01
127.83
127.70
117.44
86.80
78.51
77.22
73.31
73.13
70.89
69.78
35.08
18.27
f1 (ppm)

Compound 3

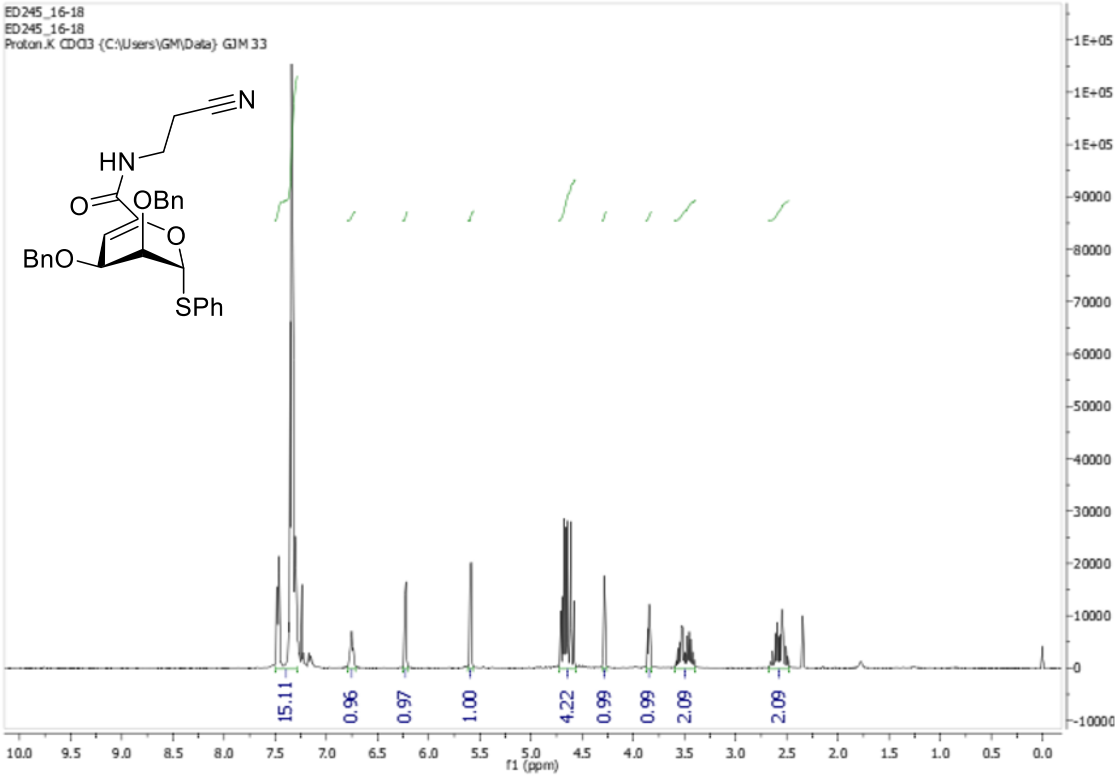
ED245_16-18
ED245_16-18
Proton.K CDCl3 {C:\Users\GM\Data} GJM 33
HN
N
O
OBn
O
BnO
SPh
15.11
0.96
0.97
1.00
4.22
0.99
0.99
2.09
2.09
f1 (ppm)

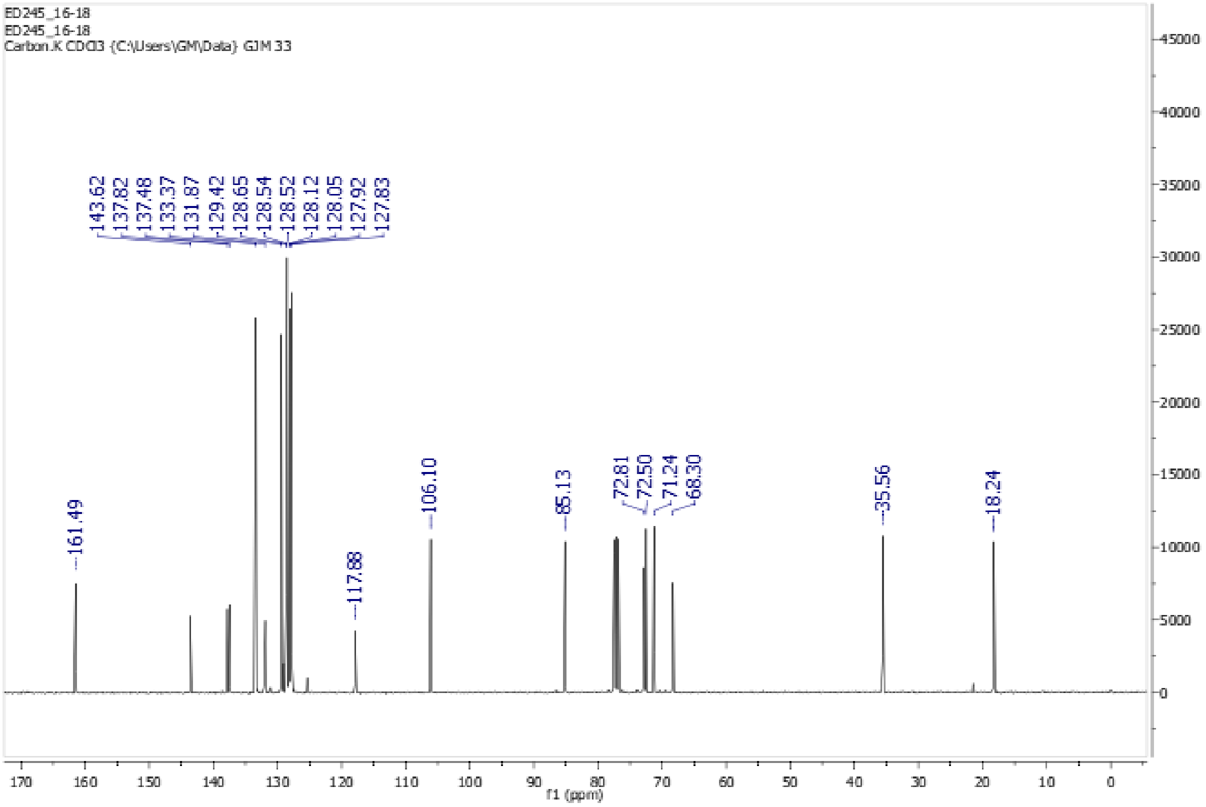
ED245_16-18
ED245_16-18
Carbon.K CDCl3 {C:\Users\GM\Data} GJM 33
143.62
137.82
137.48
133.37
131.87
129.42
128.65
128.54
128.52
128.12
128.05
127.92
127.83
161.49
117.88
106.10
85.13
72.81
72.50
71.24
68.30
35.56
18.24
f1 (ppm)

Compound 4

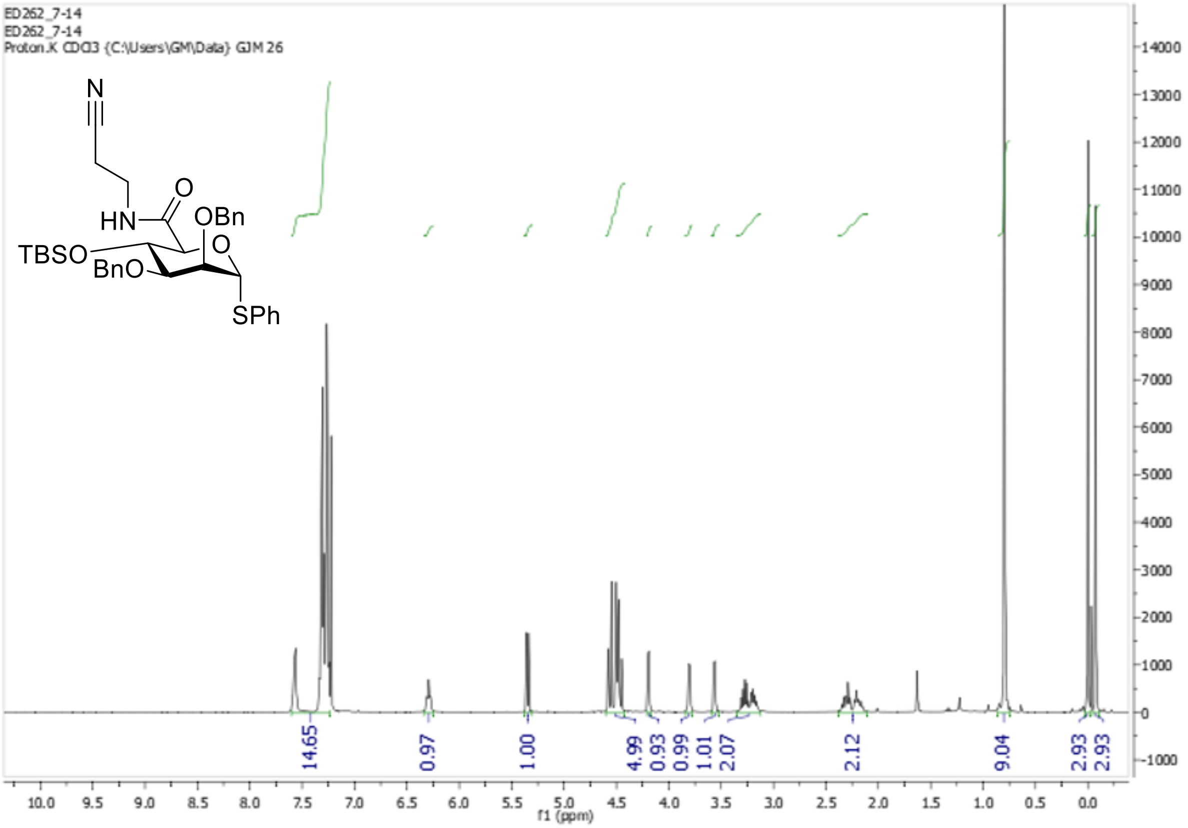

ED262_7-14
ED262_7-14
Proton.K CDCl3 {C:\Users\GM\Data} GJM 26
N
HN
O
OBn
TBSO
BnO
O
SPh
14.65
0.97
1.00
4.99
0.93
0.99
1.01
2.07
2.12
9.04
2.93
2.93
f1 (ppm)

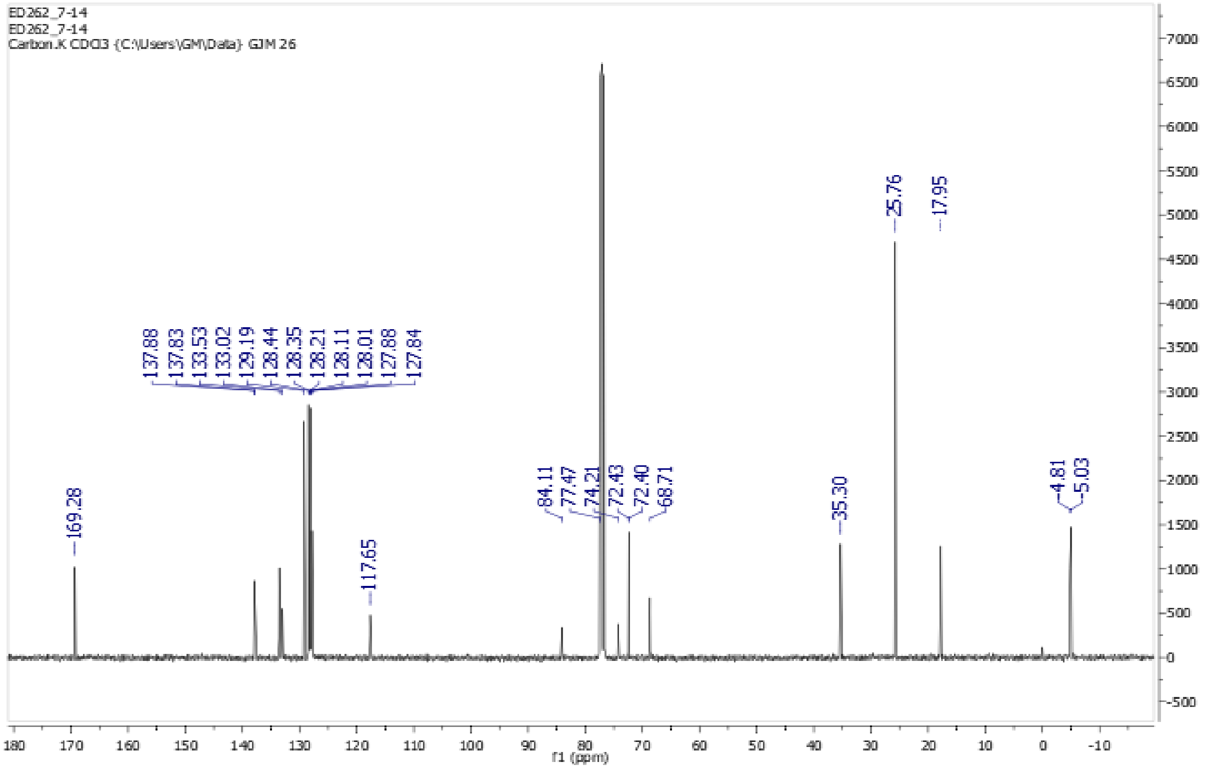

ED262_7-14
ED262_7-14
Carbon.K CDCl3 {C:\Users\GM\Data} GJM 26
169.28
137.88
137.83
133.53
133.02
129.19
128.44
128.35
128.21
128.11
128.01
127.88
127.84
117.65
84.11
77.47
74.21
72.43
72.40
68.71
35.30
25.76
17.95
4.81
5.03
f1 (ppm)

Compound 5

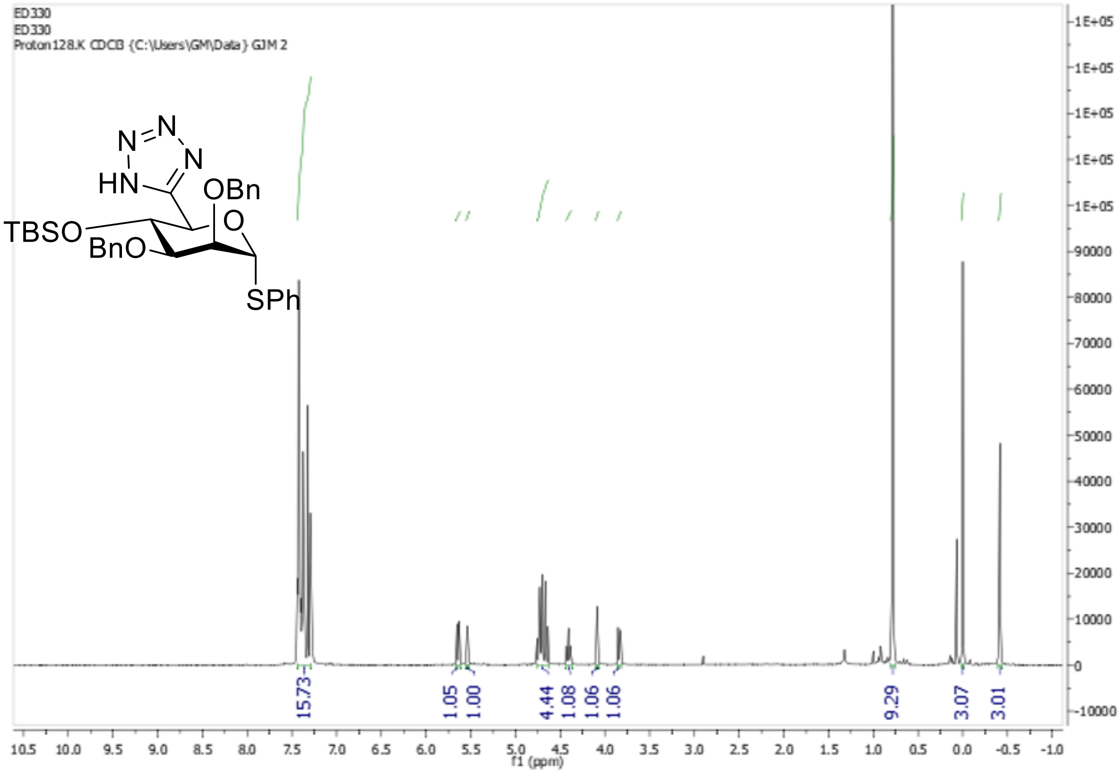
ED330
ED330
Proton128.K CDCl3 {C:\Users\GM\Data} GJM 2
N=N
N
HN
OBn
O
TBSO
BnO
SPh
15.73
1.05
1.00
4.44
1.08
1.06
1.06
9.29
3.07
3.01
f1 (ppm)

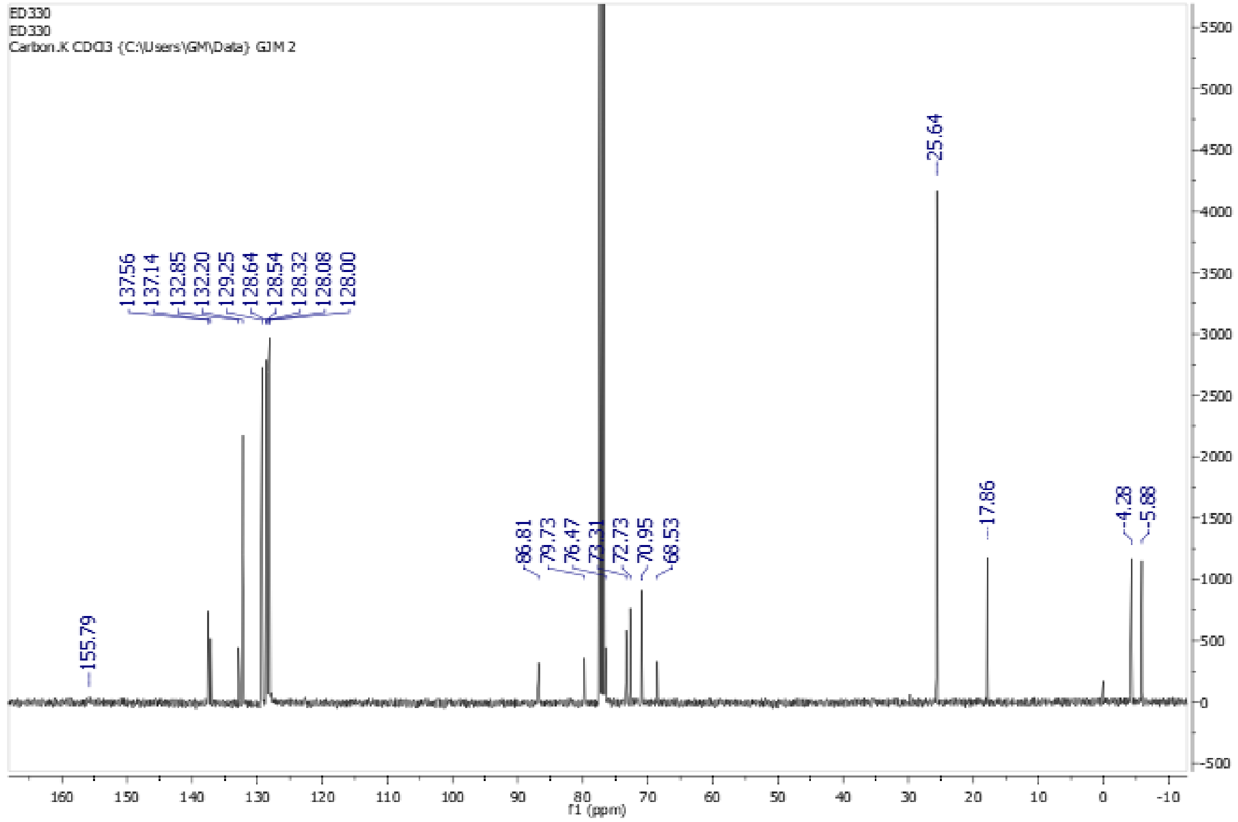
ED330
ED330
Carbon.K CDCl3 {C:\Users\GM\Data} GJM 2
155.79
137.56
137.14
132.85
132.20
129.25
128.64
128.54
128.32
128.08
128.00
86.81
79.73
76.47
73.31
72.73
70.95
68.53
25.64
17.86
-4.28
-5.88
f1 (ppm)

Phenyl 2,3-di-*O*-benzyl-6-*O*-benzoyl-1-thio-α-D-mannopyranoside

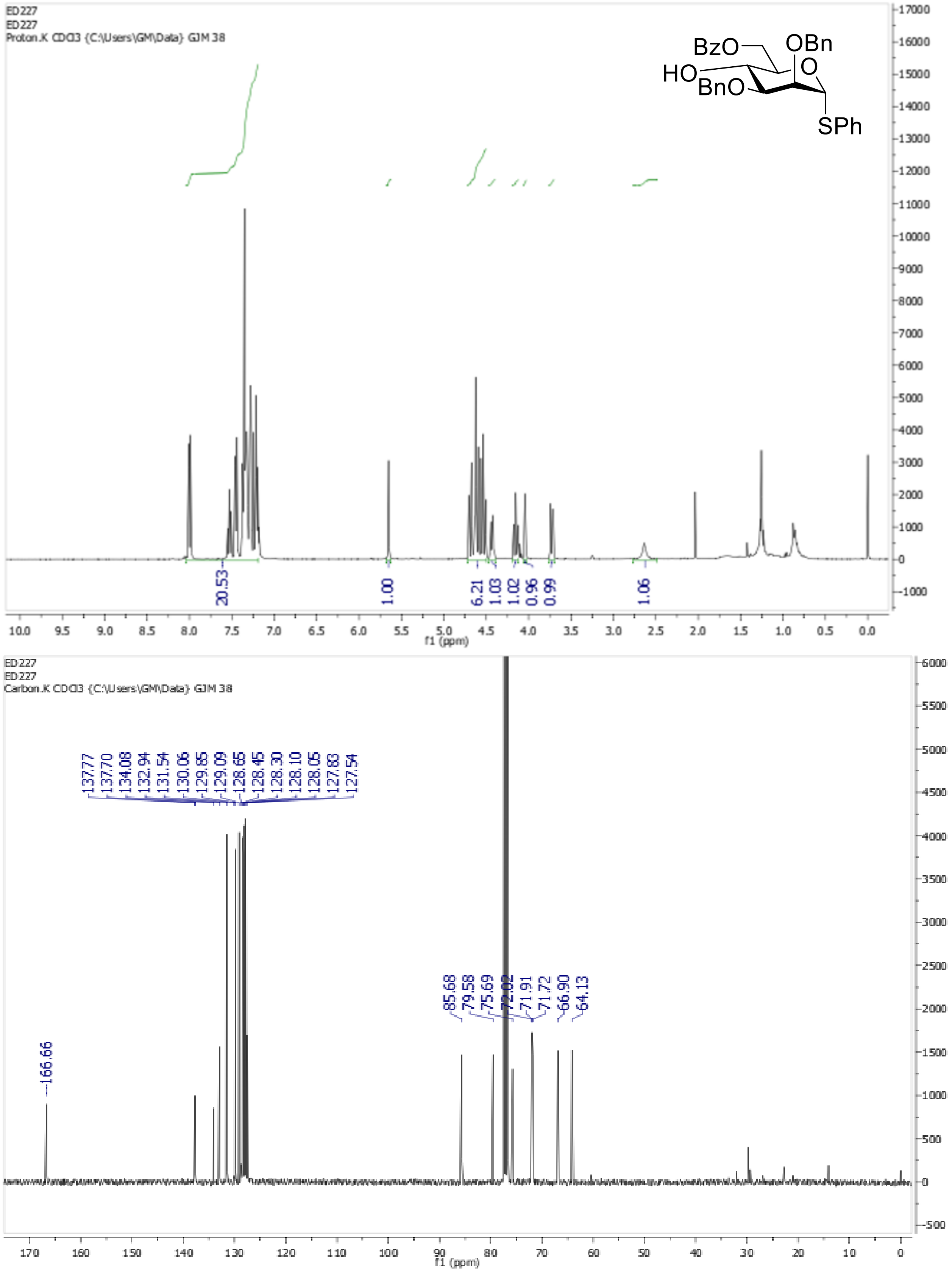

Phenyl 2,3-di-*O*-benzyl-4-*O*-*tert*-butyldimethylsilyl-6-*O*-benzoyl-1-thio-α-D-mannopyranoside

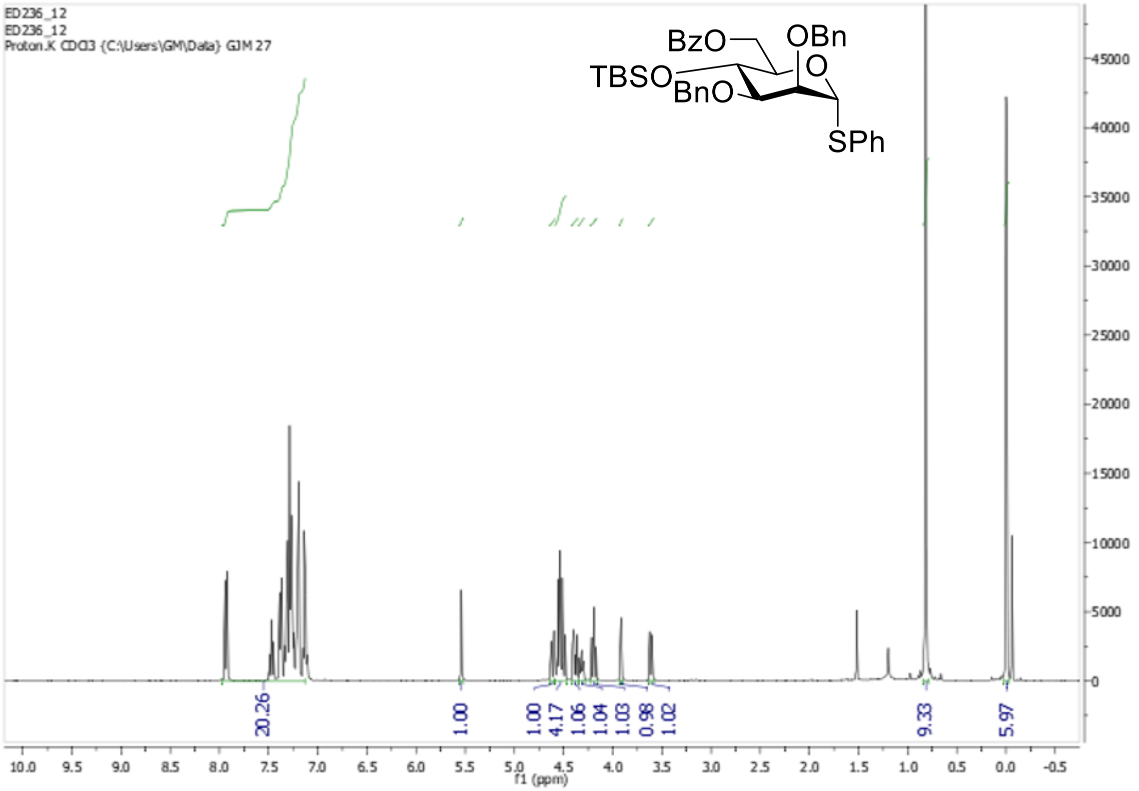

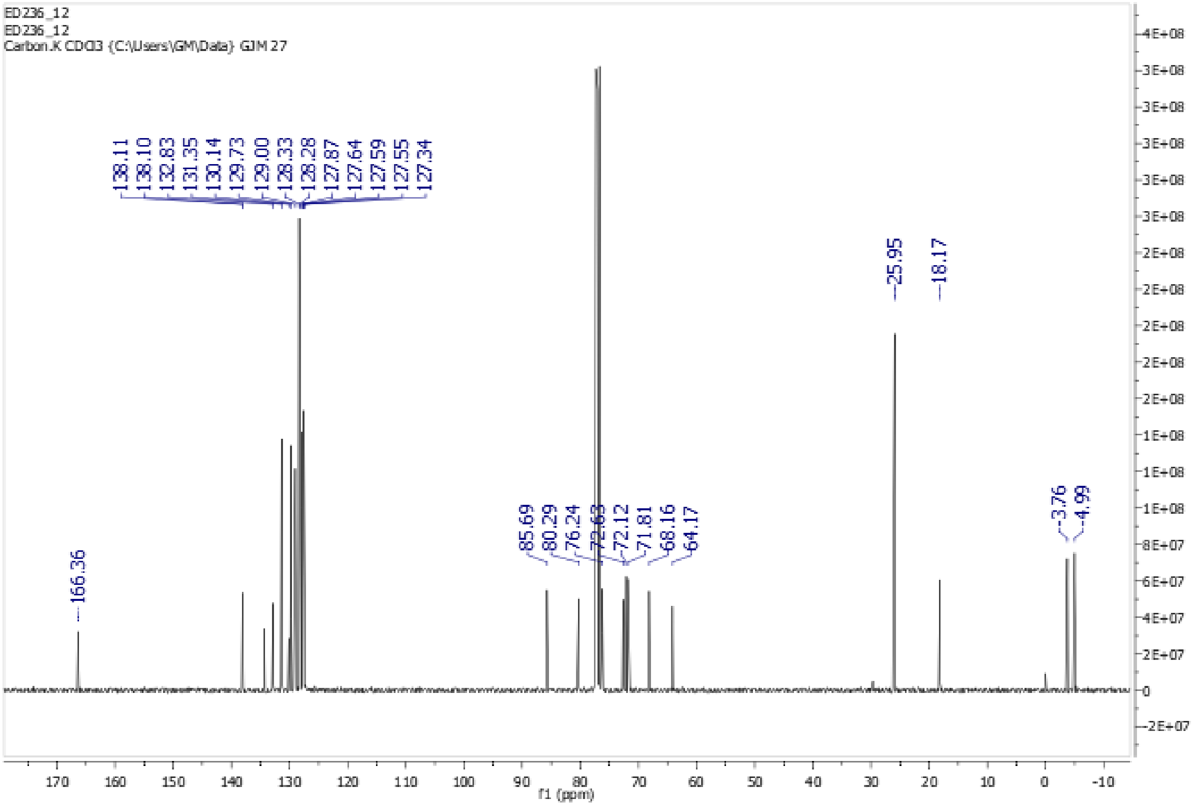

Compound 7

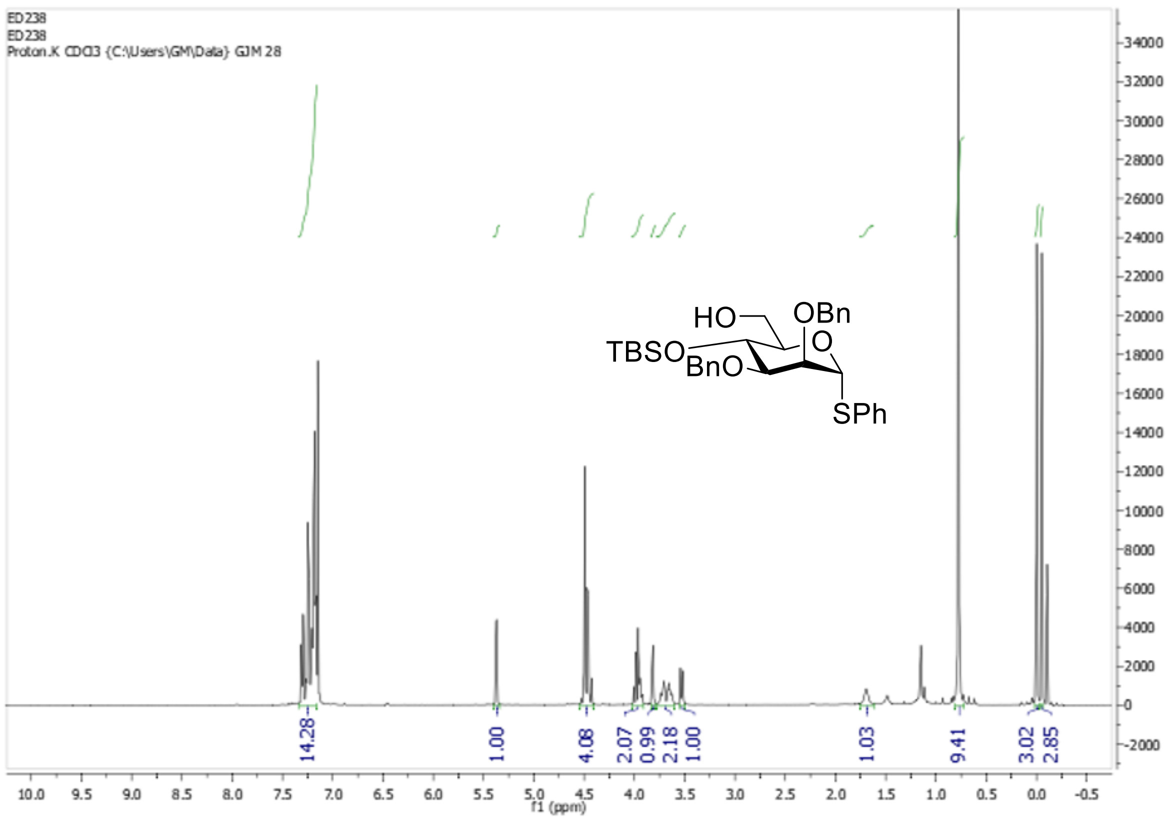
ED238
ED238
Proton.K CDCl3 {C:\Users\GM\Data} GJM 28
HO
OBn
O
TBSO
BnO
SPh
14.28
1.00
4.08
2.07
0.99
2.18
1.00
1.03
9.41
3.02
2.85
f1 (ppm)

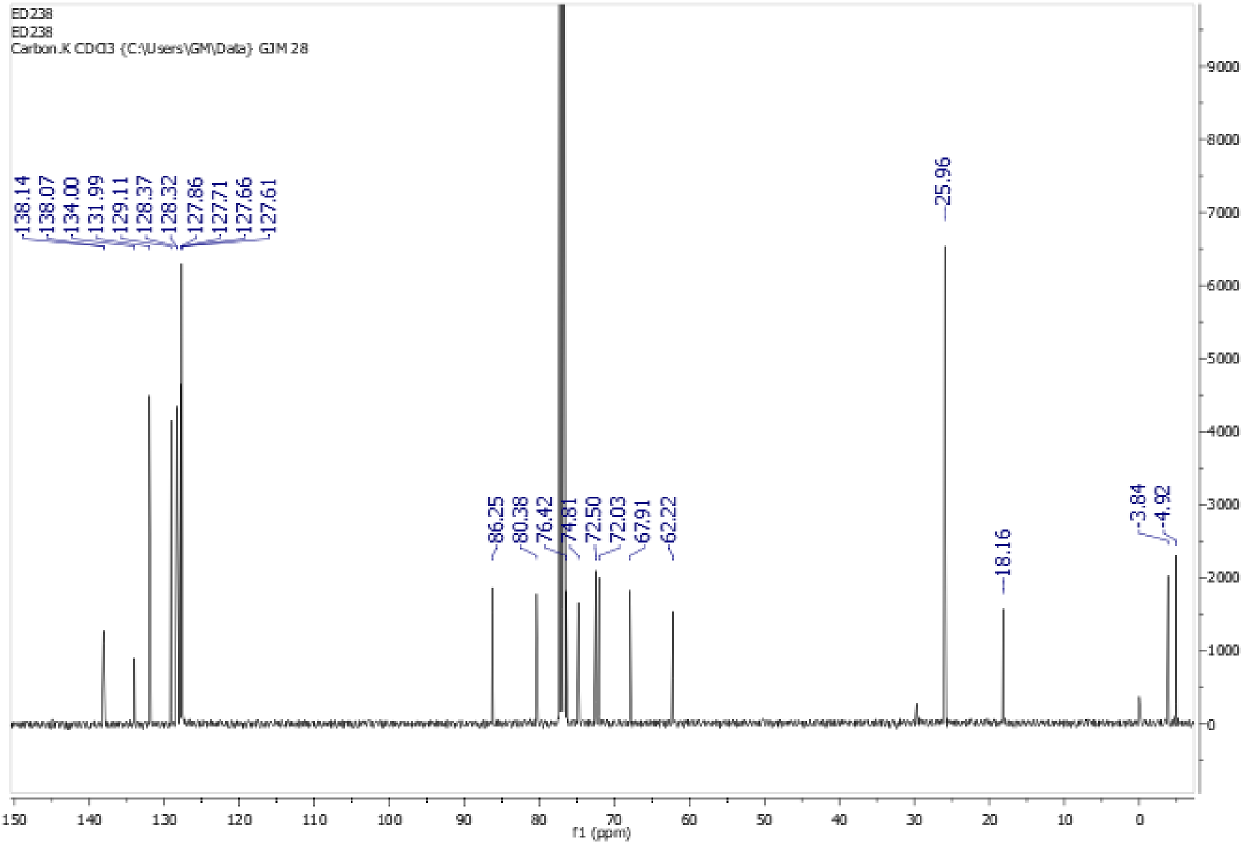
ED238
ED238
Carbon.K CDCl3 {C:\Users\GM\Data} GJM 28
138.14
138.07
134.00
131.99
129.11
128.37
128.32
127.86
127.71
127.66
127.61
86.25
80.38
76.42
74.81
72.50
72.03
67.91
62.22
25.96
18.16
-3.84
-4.92
f1 (ppm)

C6 aldehyde thioglycoside intermediate

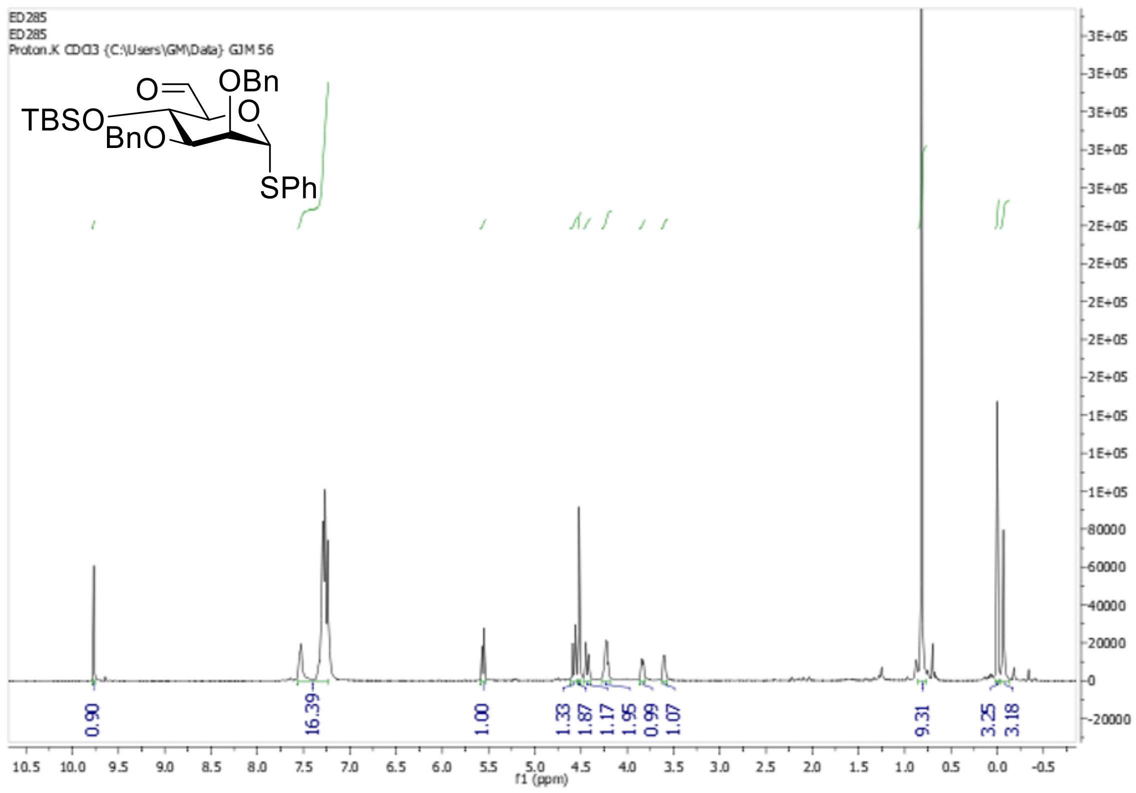

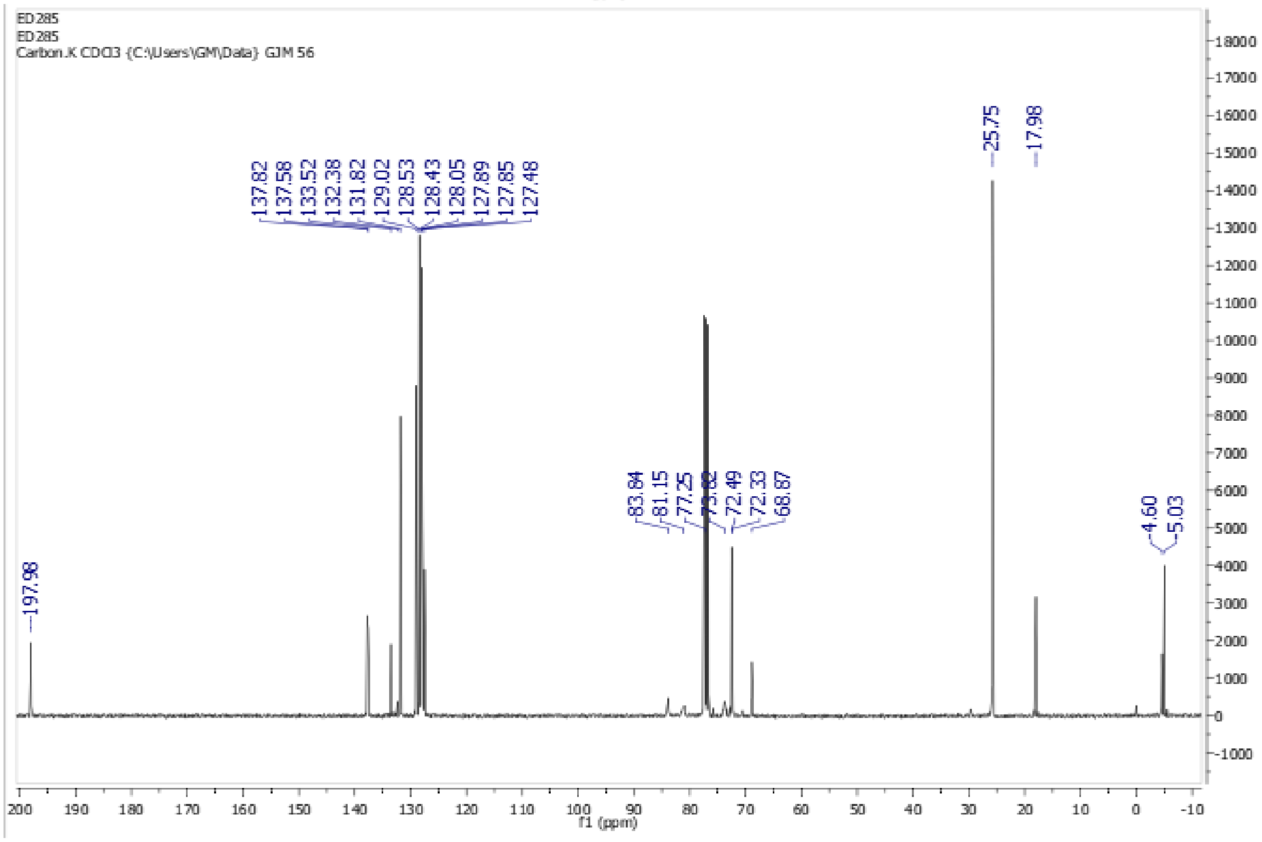

Compound 8

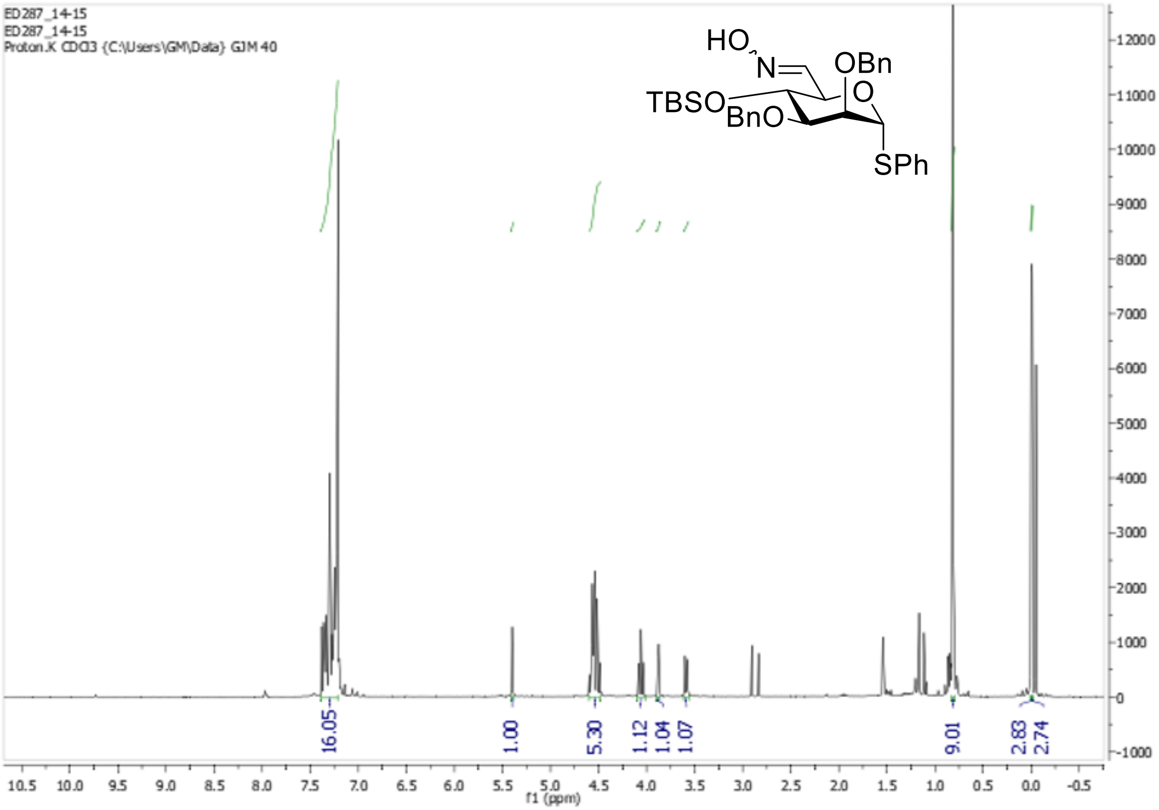
ED287_14-15
ED287_14-15
Proton.K CDCl3 {C:\Users\GM\Data} GJM 40
HO
N
OBn
O
TBSO
BnO
SPh
16.05
1.00
5.30
1.12
1.04
1.07
9.01
2.83
2.74
f1 (ppm)

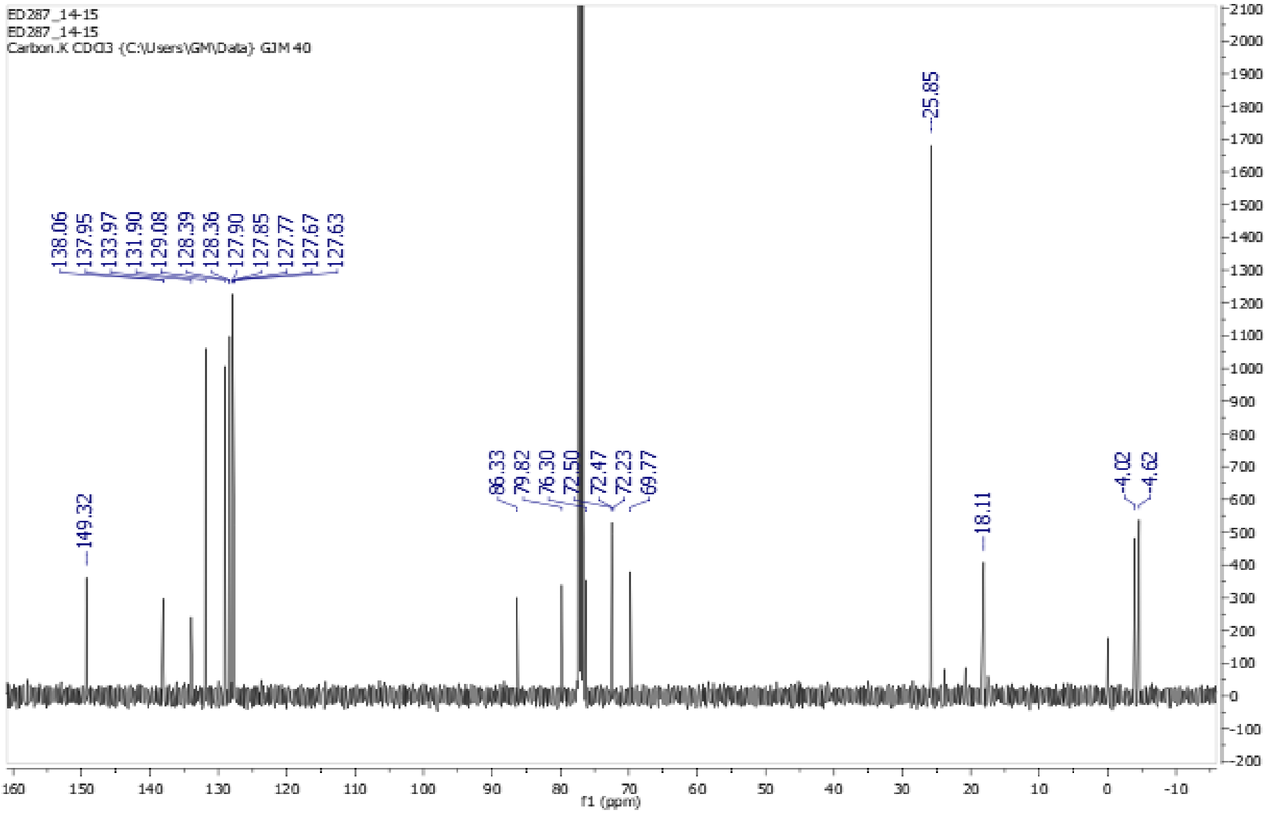
ED287_14-15
ED287_14-15
Carbon.K CDCl3 {C:\Users\GM\Data} GJM 40
149.32
138.06
137.95
133.97
131.90
129.08
128.39
128.36
127.90
127.85
127.77
127.67
127.63
86.33
79.82
76.30
72.50
72.47
72.23
69.77
25.85
18.11
-4.02
-4.62
f1 (ppm)

Compound 9

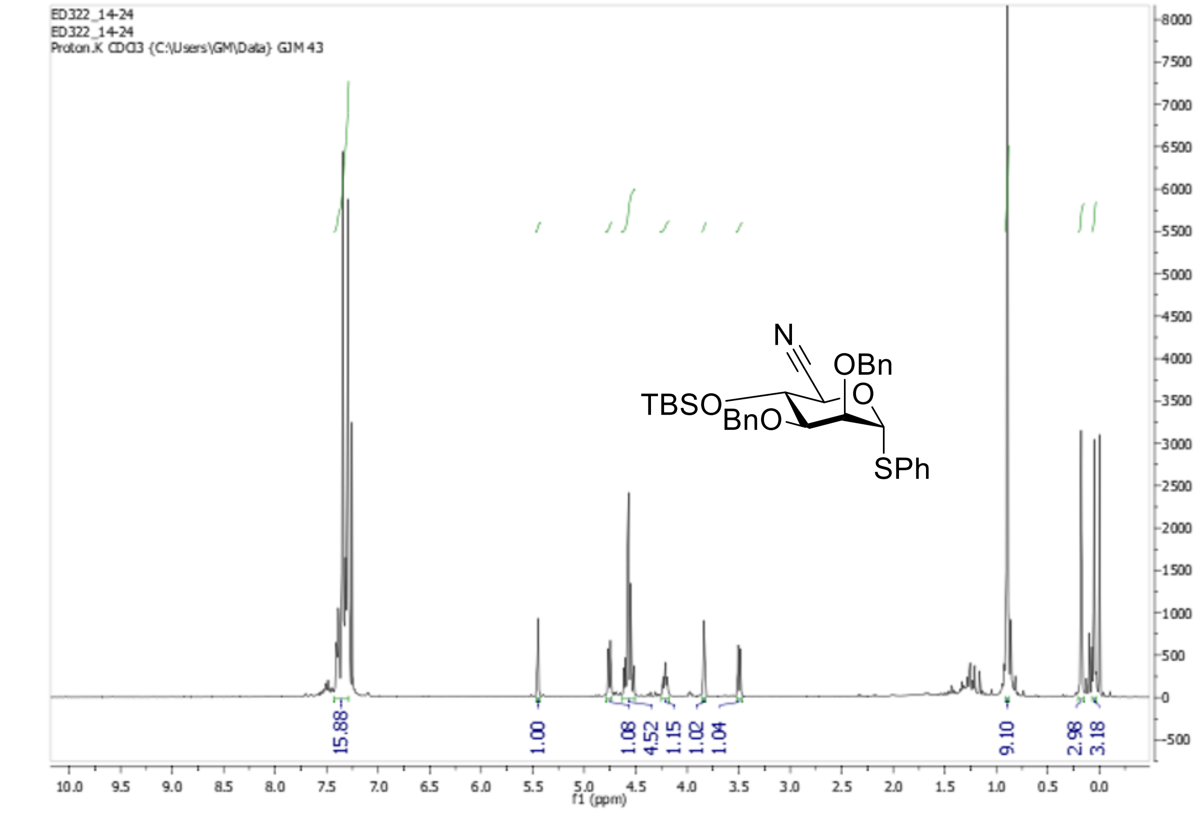
ED322_14-24
ED322_14-24
Proton.K CDCl3 {C:\Users\GM\Data} GJM 43
TBSO
BnO
N
OBn
O
SPh
15.88
1.00
1.08
4.52
1.15
1.02
1.04
9.10
2.98
3.18
f1 (ppm)

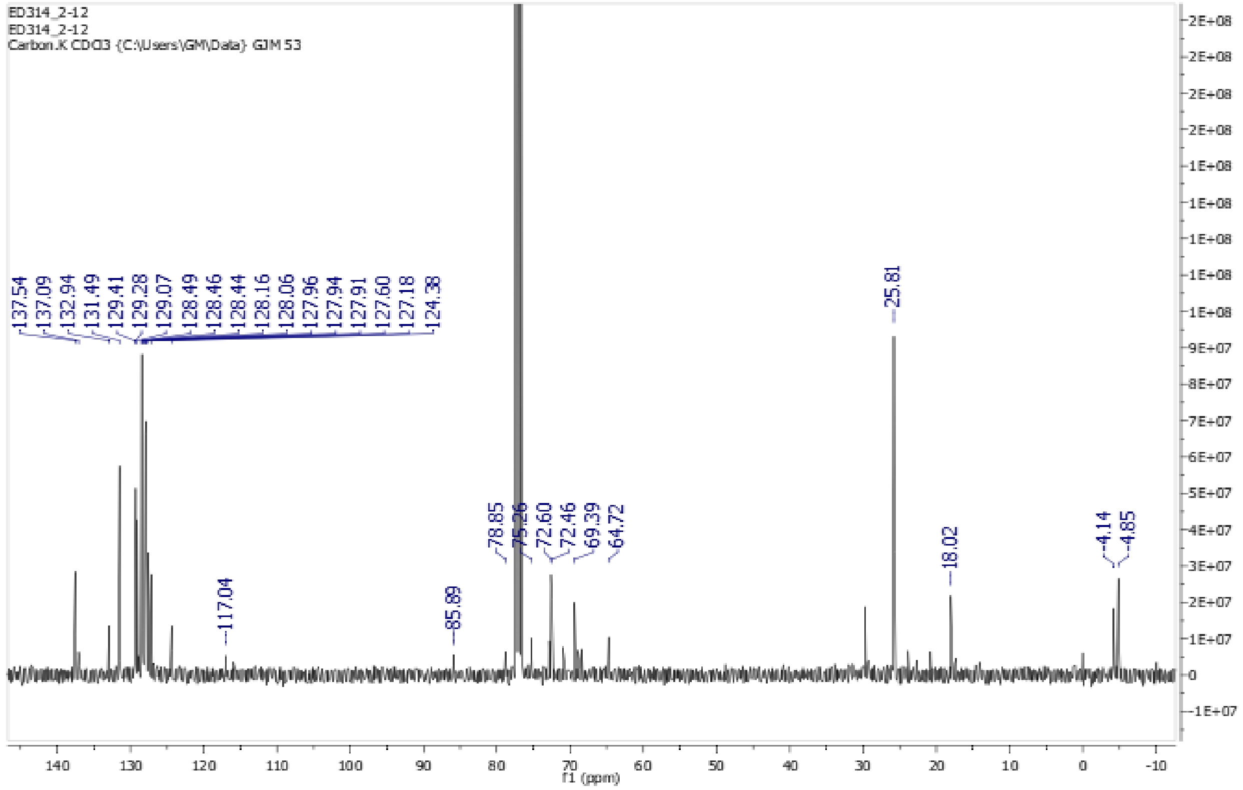
ED314_2-12
ED314_2-12
Carbon.K CDCl3 {C:\Users\GM\Data} GJM 53
137.54
137.09
132.94
131.49
129.41
129.28
129.07
128.49
128.46
128.44
128.16
128.06
127.96
127.94
127.91
127.60
127.18
124.38
117.04
85.89
78.85
75.26
72.60
72.46
69.39
64.72
25.81
18.02
-4.14
-4.85
f1 (ppm)

Compound 10

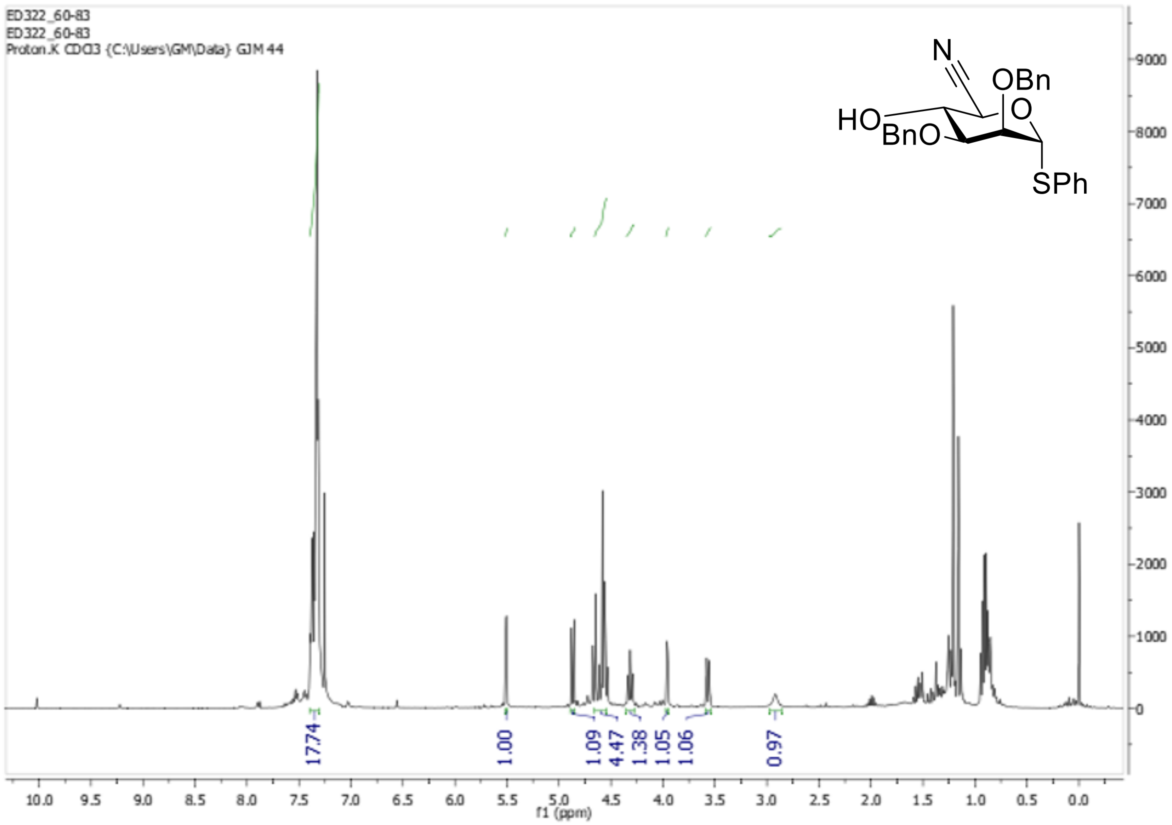
ED322_60-83
ED322_60-83
Proton.K CDCl3 {C:\Users\GM\Data} GJM 44
N
OBn
HO
BnO
O
SPh
17.74
1.00
1.09
4.47
1.38
1.05
1.06
0.97
f1 (ppm)

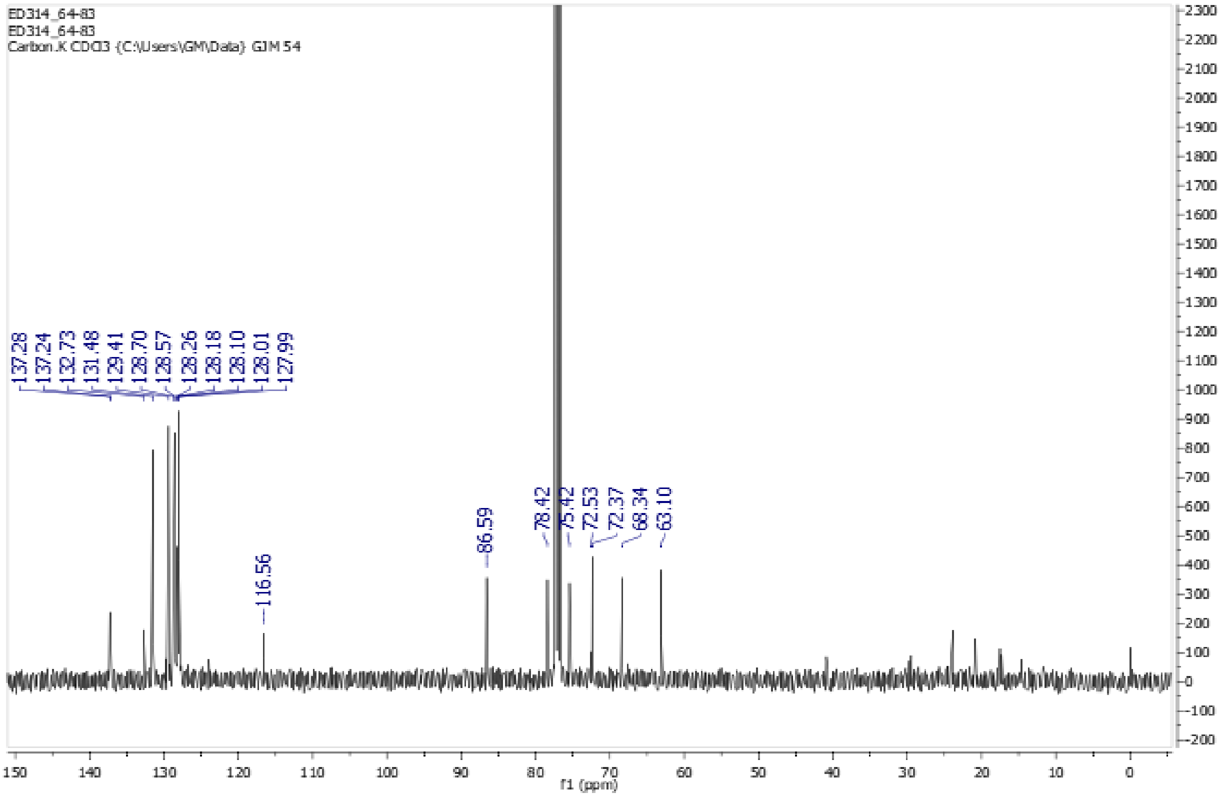
ED314_64-83
ED314_64-83
Carbon.K CDCl3 {C:\Users\GM\Data} GJM 54
137.28
137.24
132.73
131.48
129.41
128.70
128.57
128.26
128.18
128.10
128.01
127.99
116.56
86.59
78.42
75.42
72.53
72.37
68.34
63.10
f1 (ppm)

Compound 11

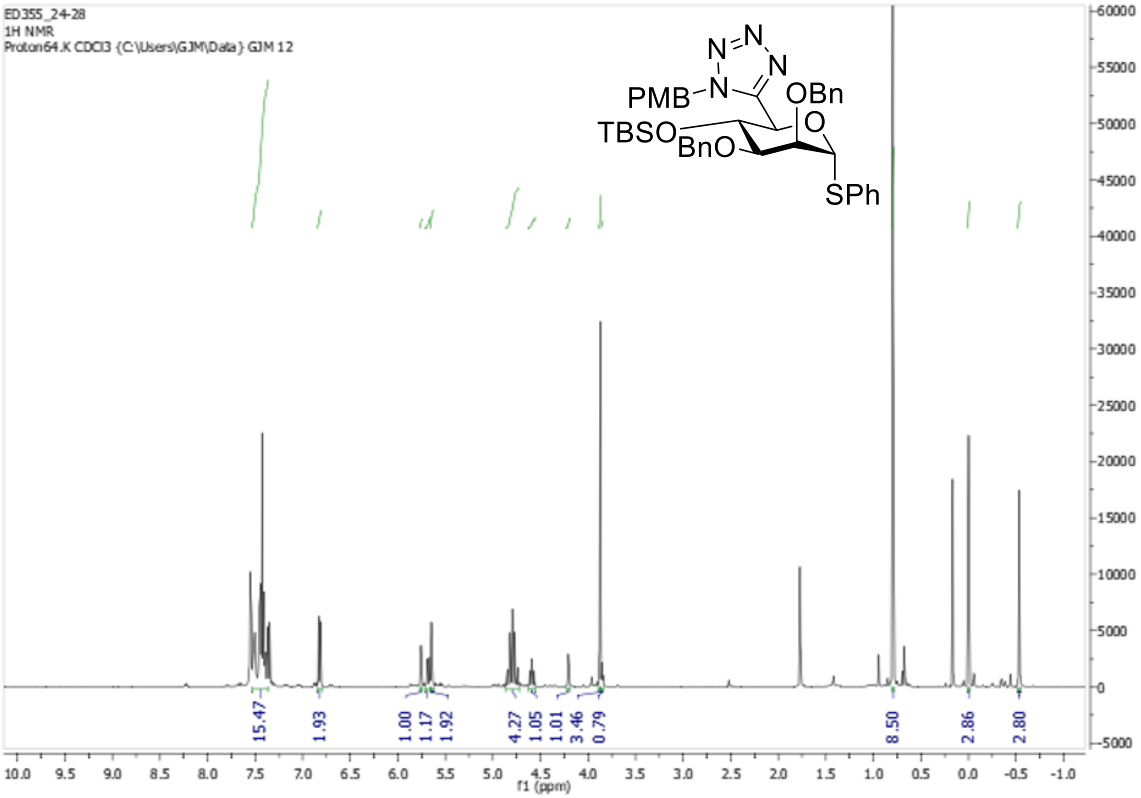

ED355_24-28
1H NMR
Proton64.K CDCl3 {C:\Users\GJM\Data} GJM 12
PMB
TBSO
BnO
OBn
O
SPh
N
15.47
1.93
1.00
1.17
1.92
4.27
1.05
1.01
3.46
0.79
8.50
2.86
2.80
f1 (ppm)

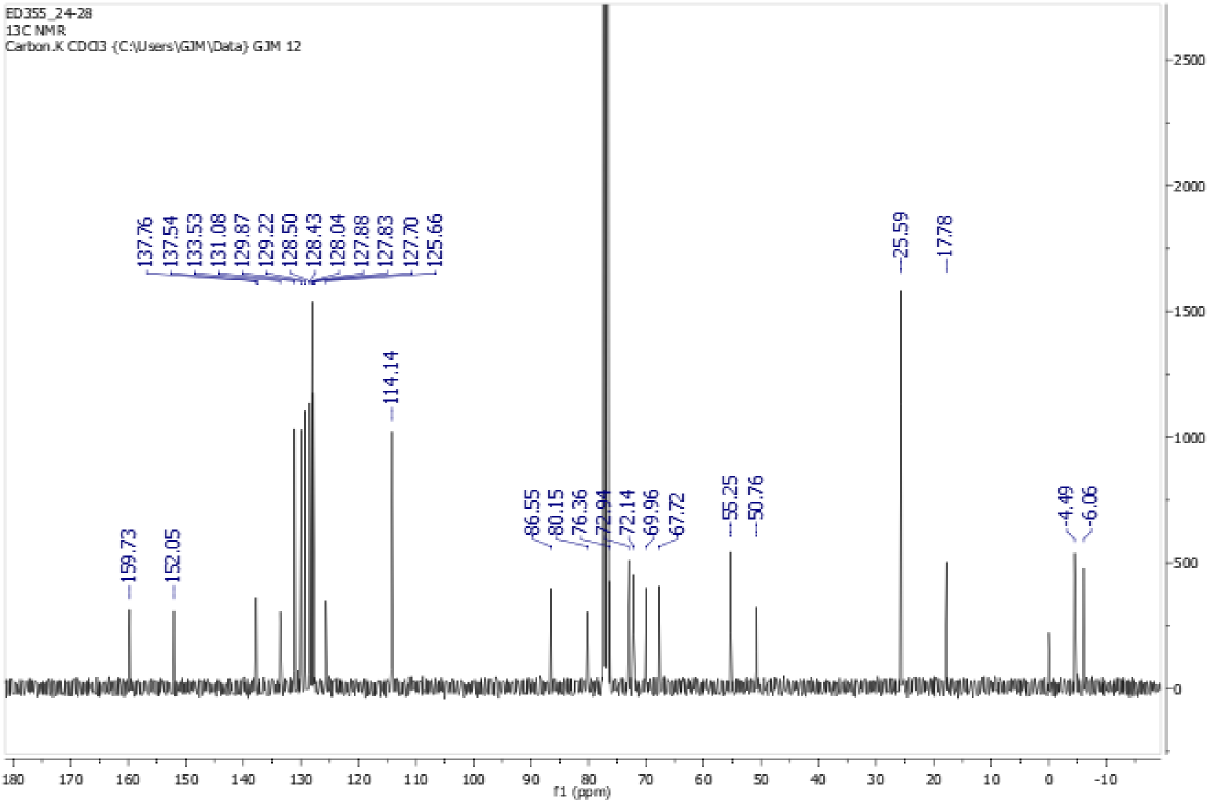

ED355_24-28
13C NMR
Carbon.K CDCl3 {C:\Users\GJM\Data} GJM 12
159.73
152.05
137.76
137.54
133.53
131.08
129.87
129.22
128.50
128.43
128.04
127.88
127.83
127.70
125.66
114.14
86.55
80.15
76.36
72.94
72.14
69.96
67.72
55.25
50.76
25.59
17.78
-4.49
-6.06
f1 (ppm)

Coupled HSQC

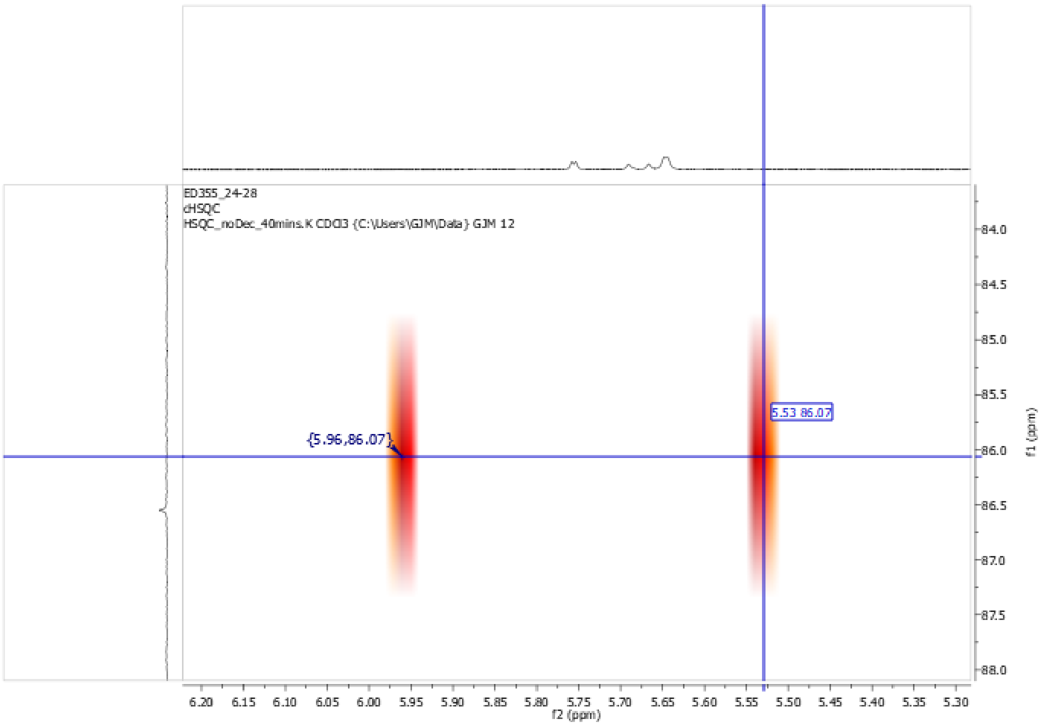

HMBC

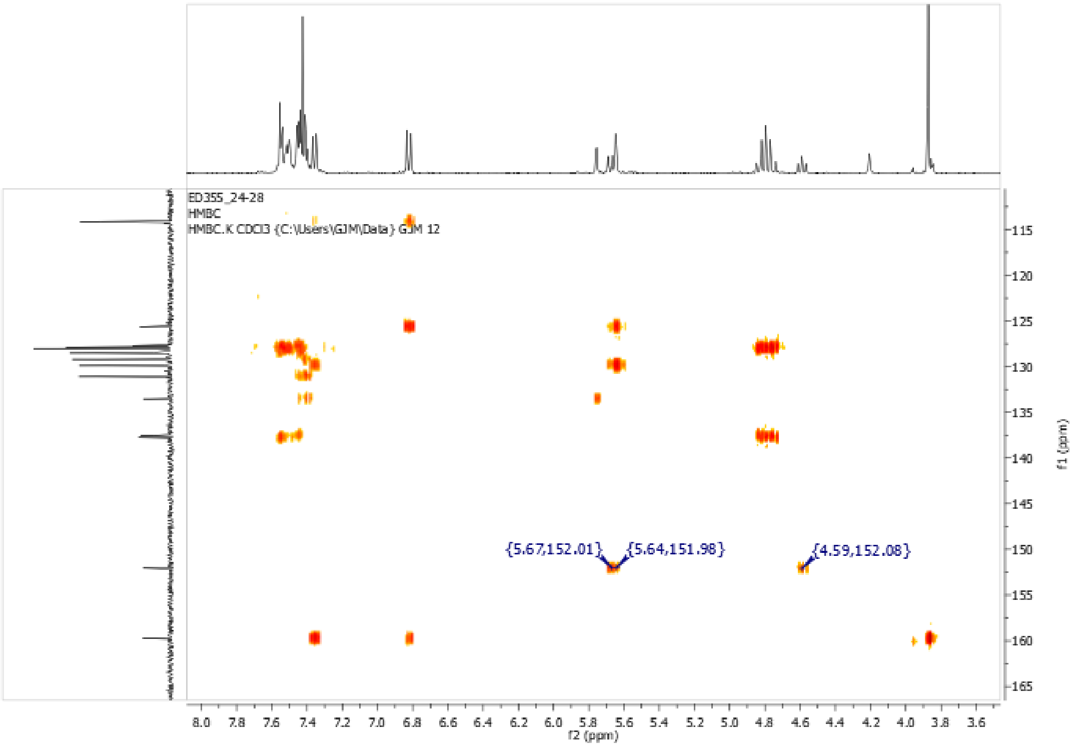

Compound 12

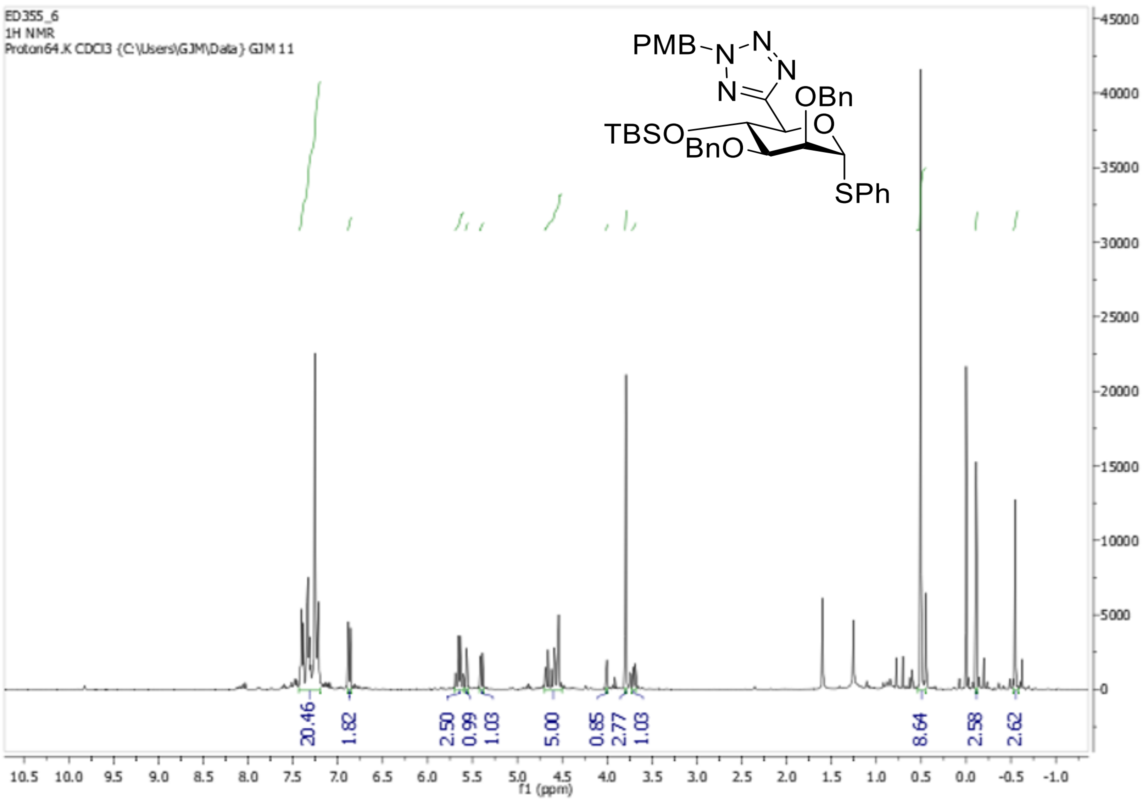

ED355_6
1H NMR
Proton64.K CDCl3 {C:\Users\GJM\Data} GJM 11
PMB
OBn
TBSO
BnO
O
SPh
20.46
1.82
2.50
0.99
1.03
5.00
0.85
2.77
1.03
8.64
2.58
2.62
f1 (ppm)

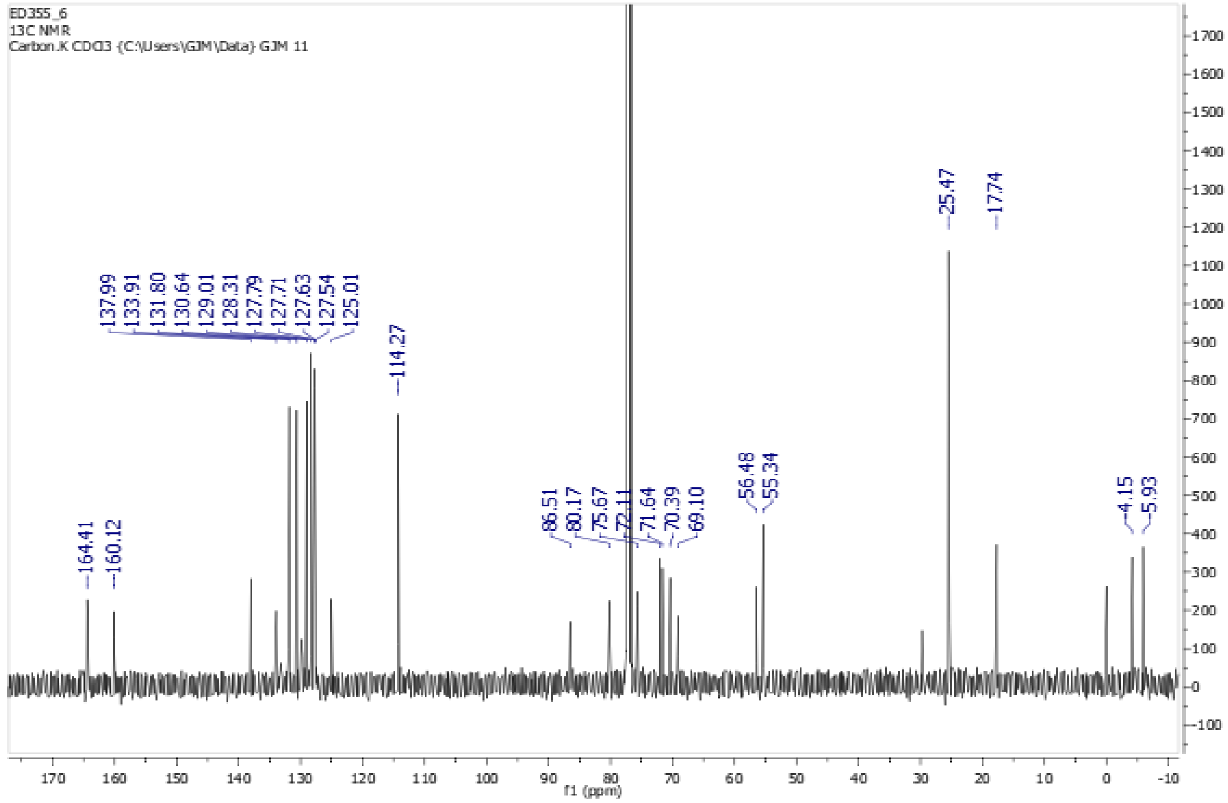

ED355_6
13C NMR
Carbon.K CDCl3 {C:\Users\GJM\Data} GJM 11
164.41
160.12
137.99
133.91
131.80
130.64
129.01
128.31
127.79
127.71
127.63
127.54
125.01
114.27
86.51
80.17
75.67
72.11
71.64
70.39
69.10
56.48
55.34
25.47
17.74
-4.15
-5.93
f1 (ppm)

Coupled HSQC

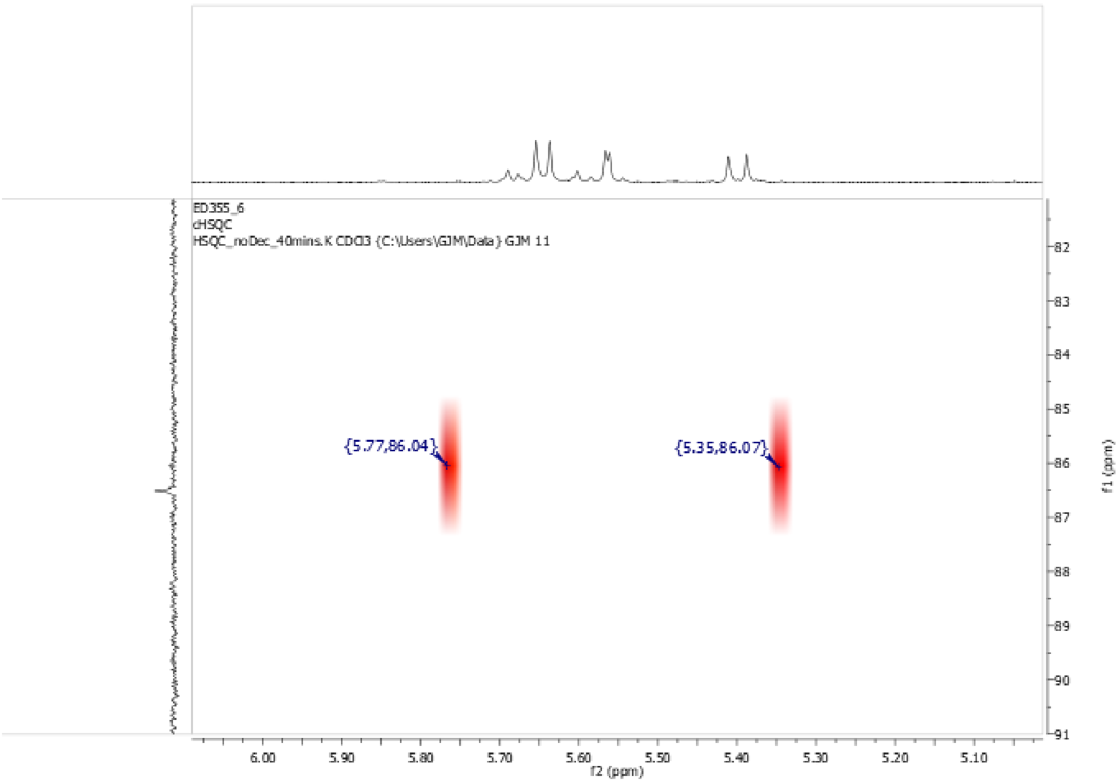

HMBC

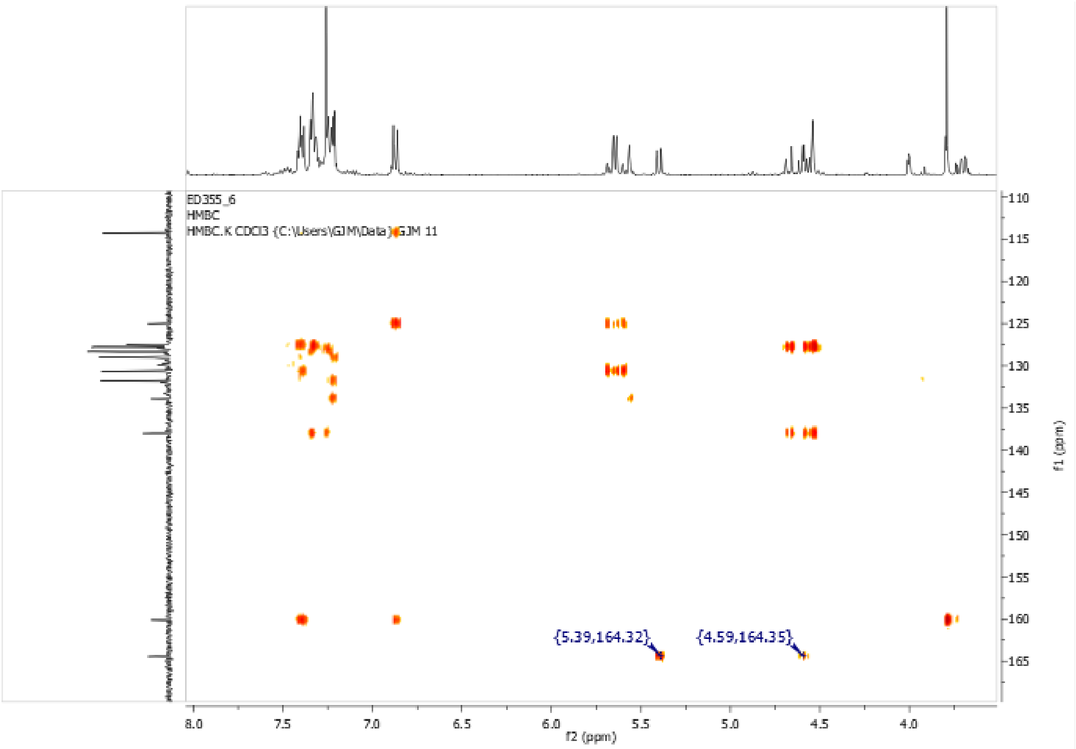

Phenyl 2,3-di-*O*-benzyl-4-*O*-*tert*-butyldimethylsilyl-6-*C*-(1*H*-tetrazolyl)-1-thio-α-D-mannopyranoside triethylammonium salt

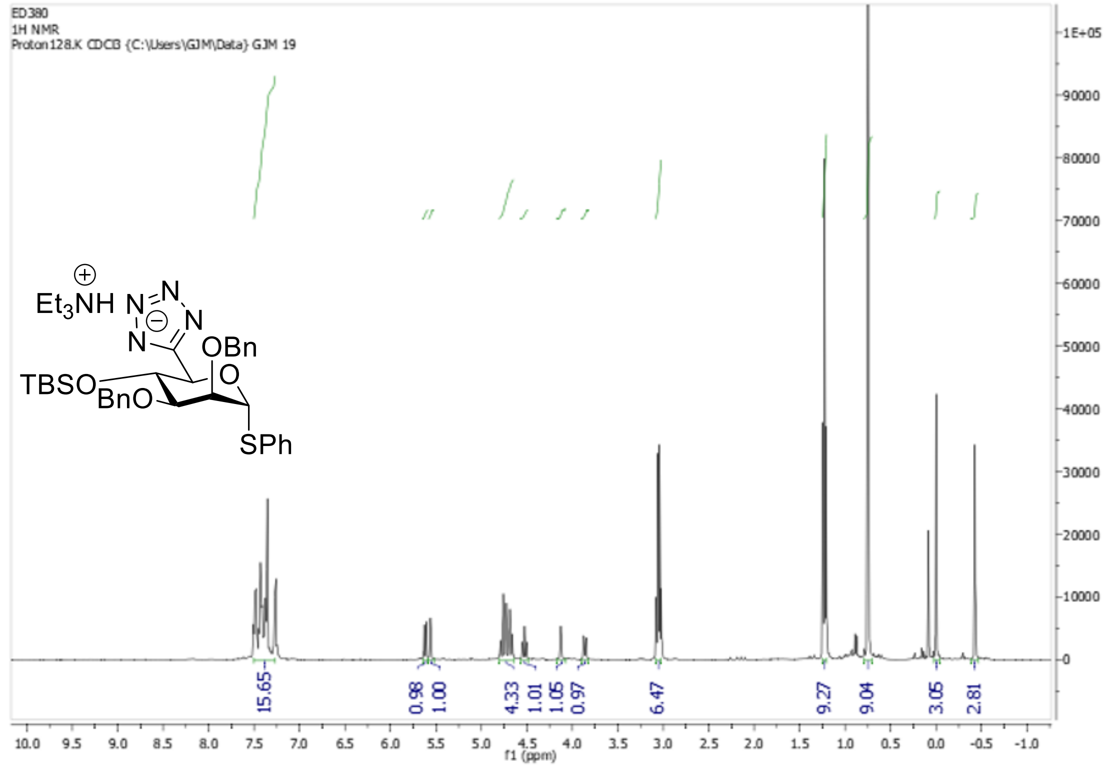

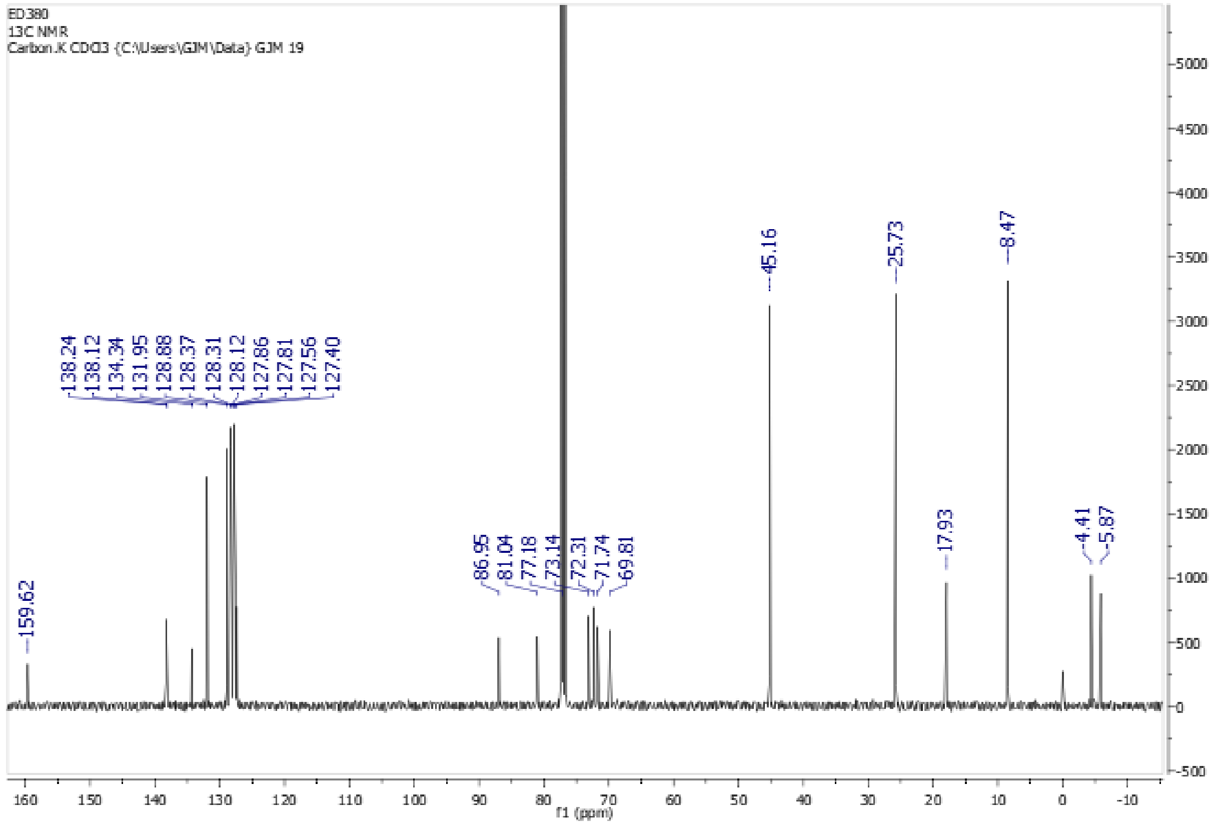

Compounds 13 and 14

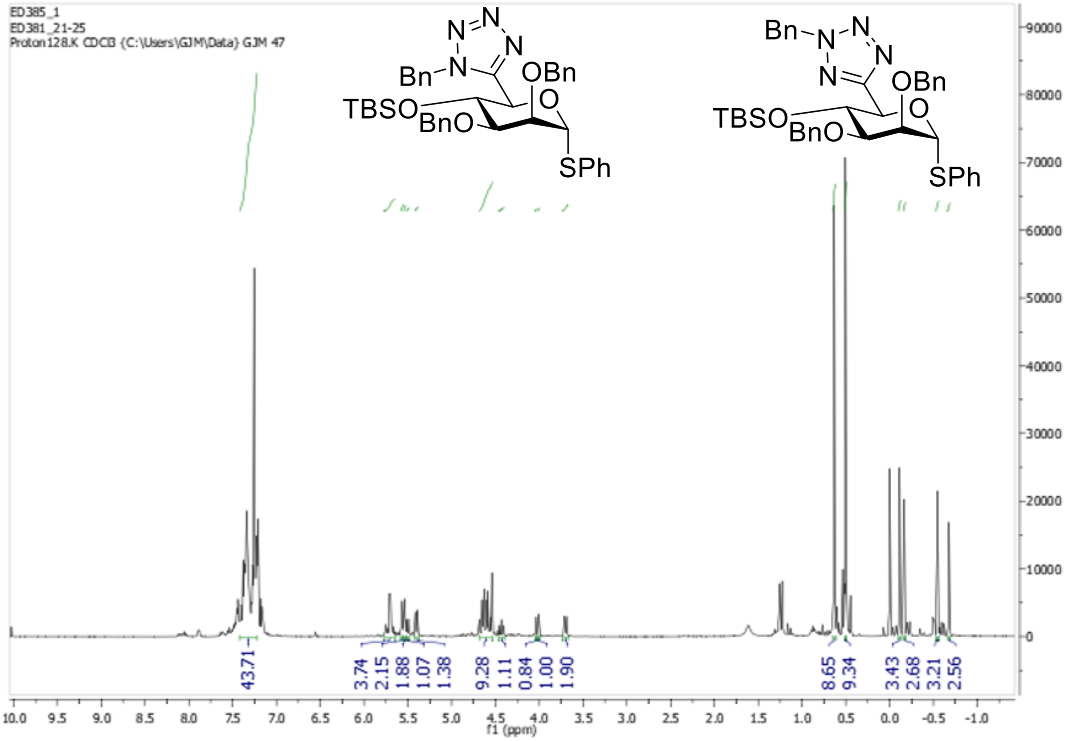

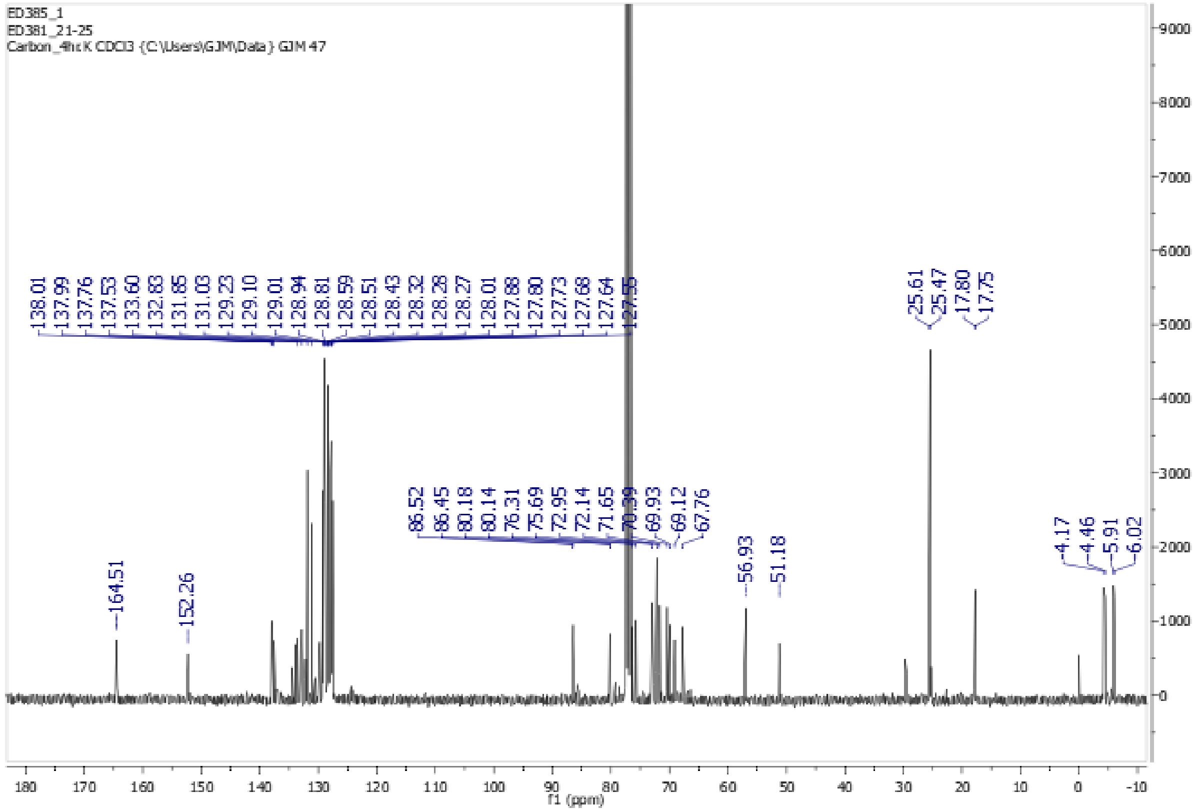

Coupled HSQC

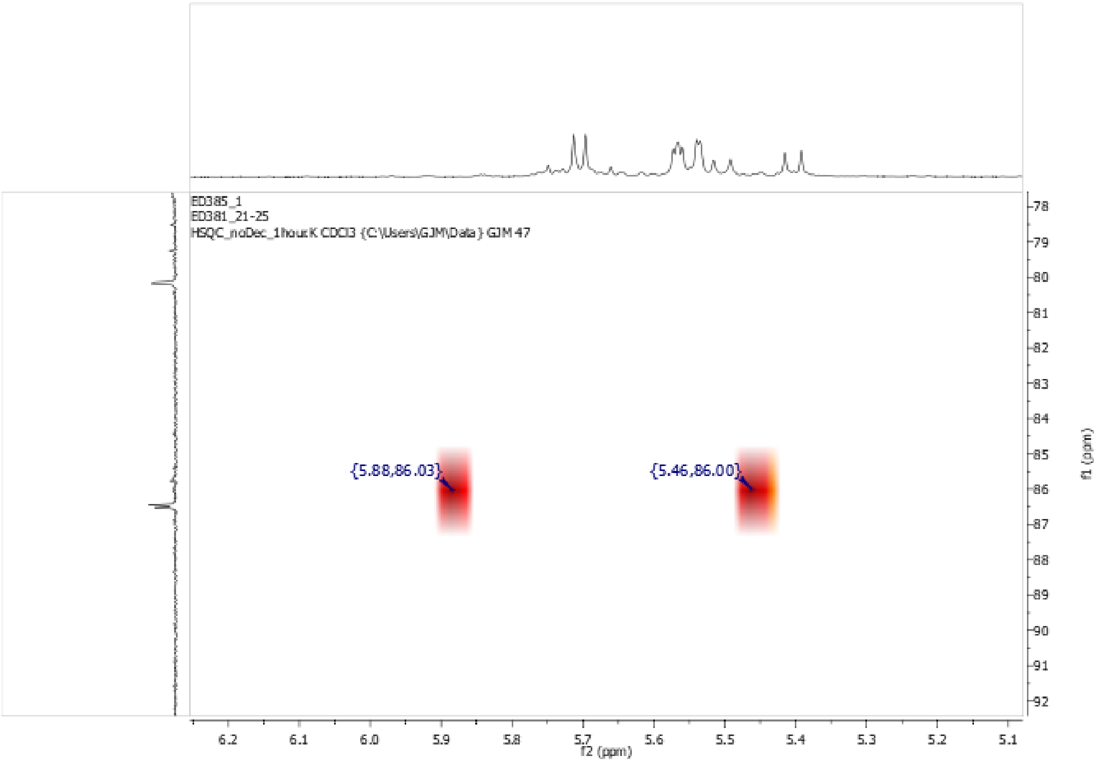

HMBC

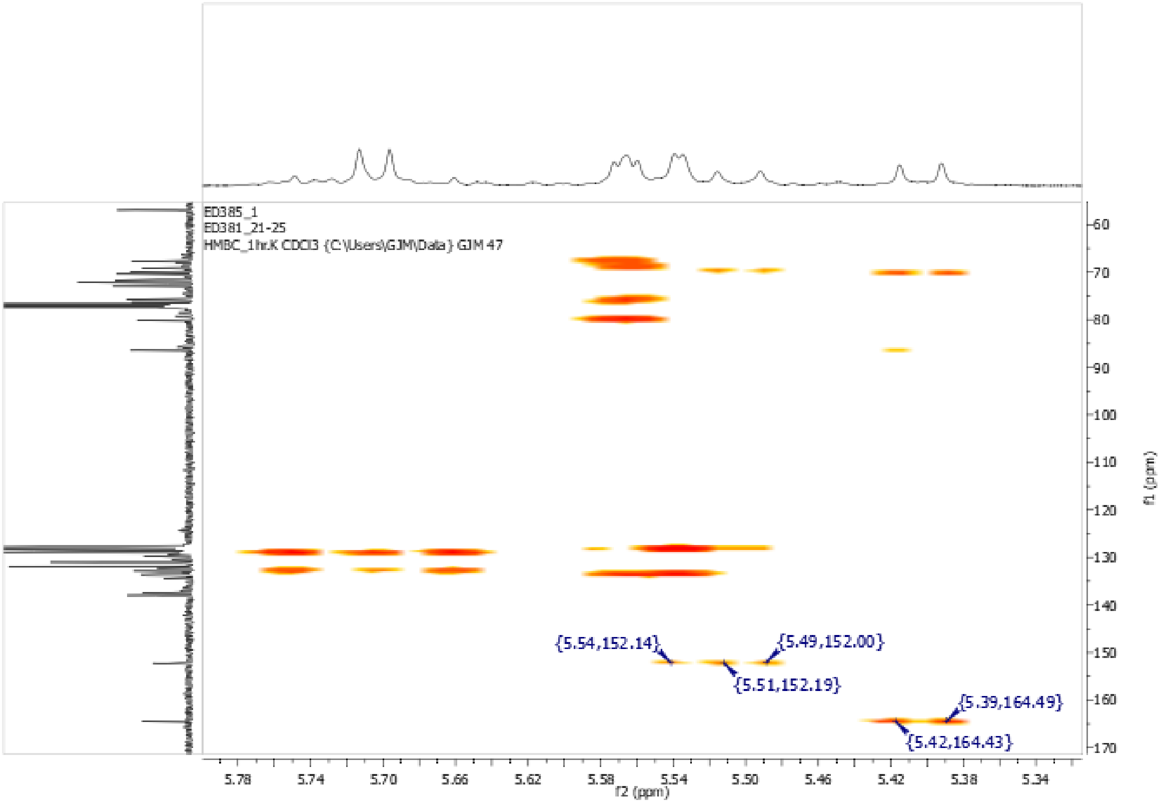

C-6 Aldehyde thioglycoside intermediate

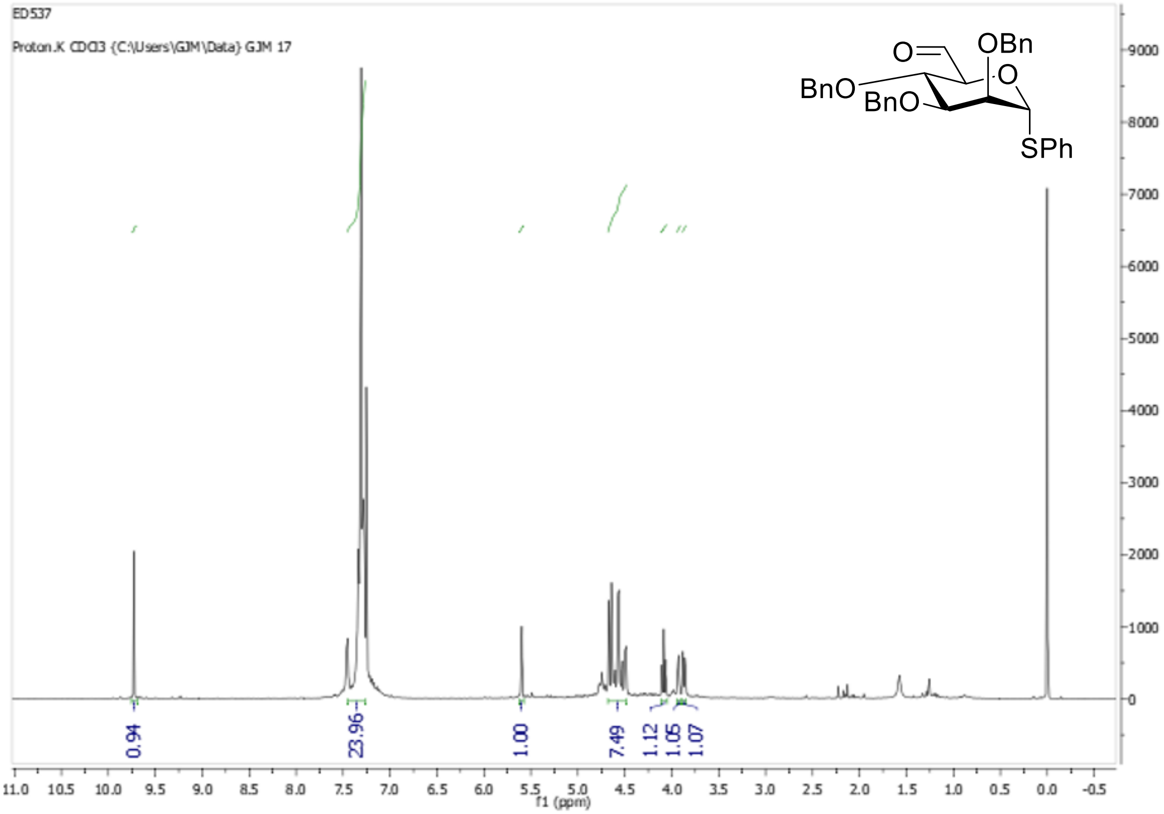

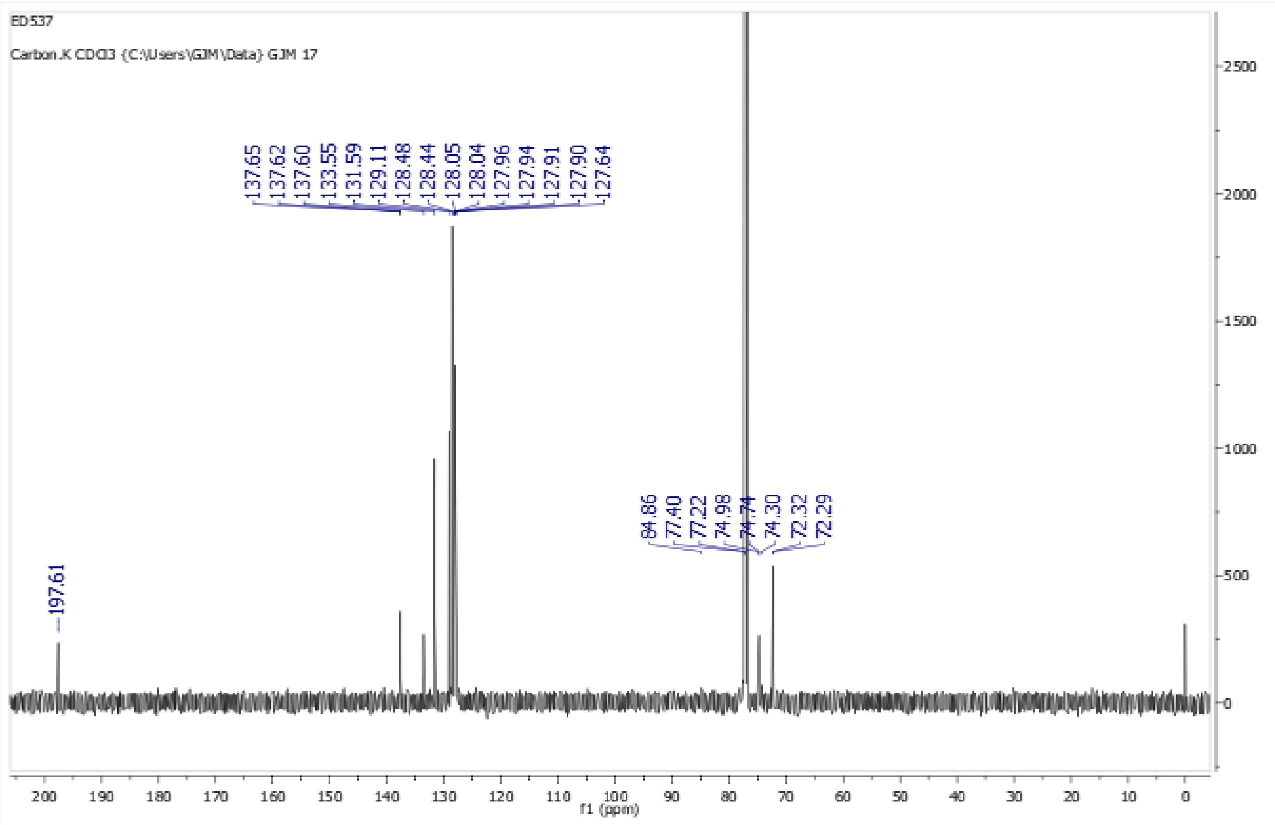

C-6 Oxime thioglycoside intermediate

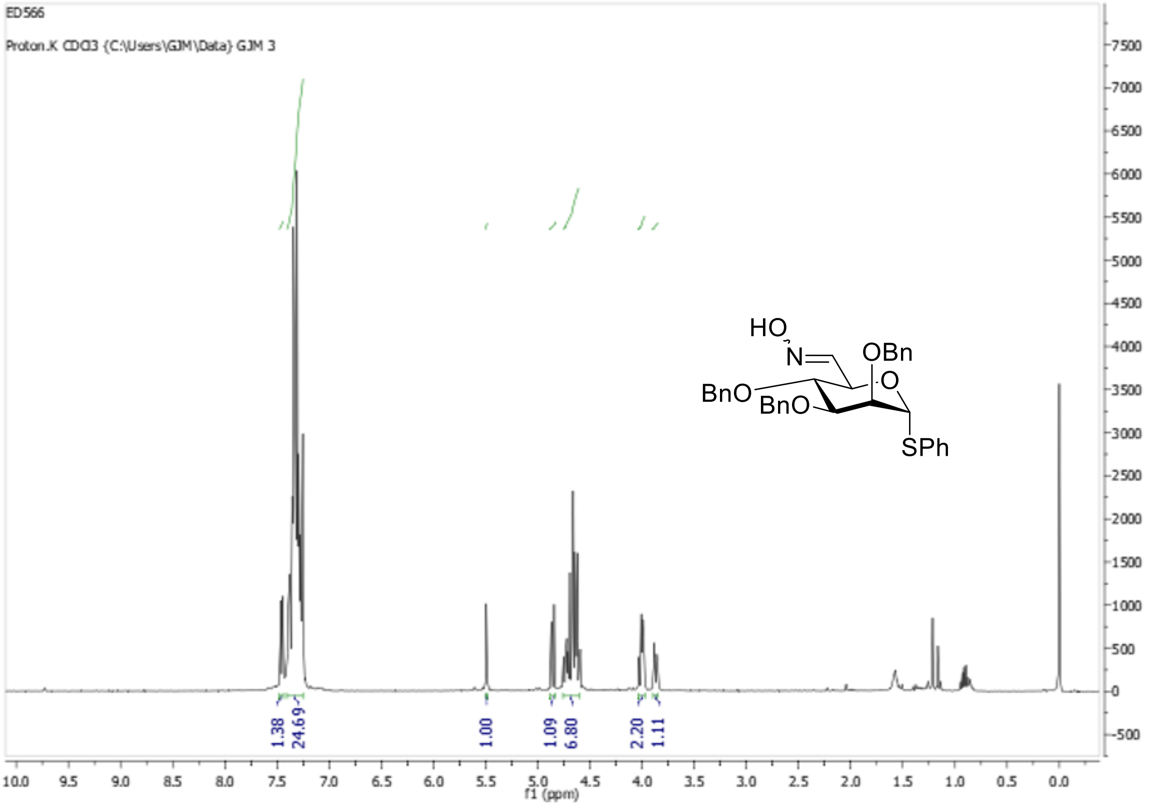

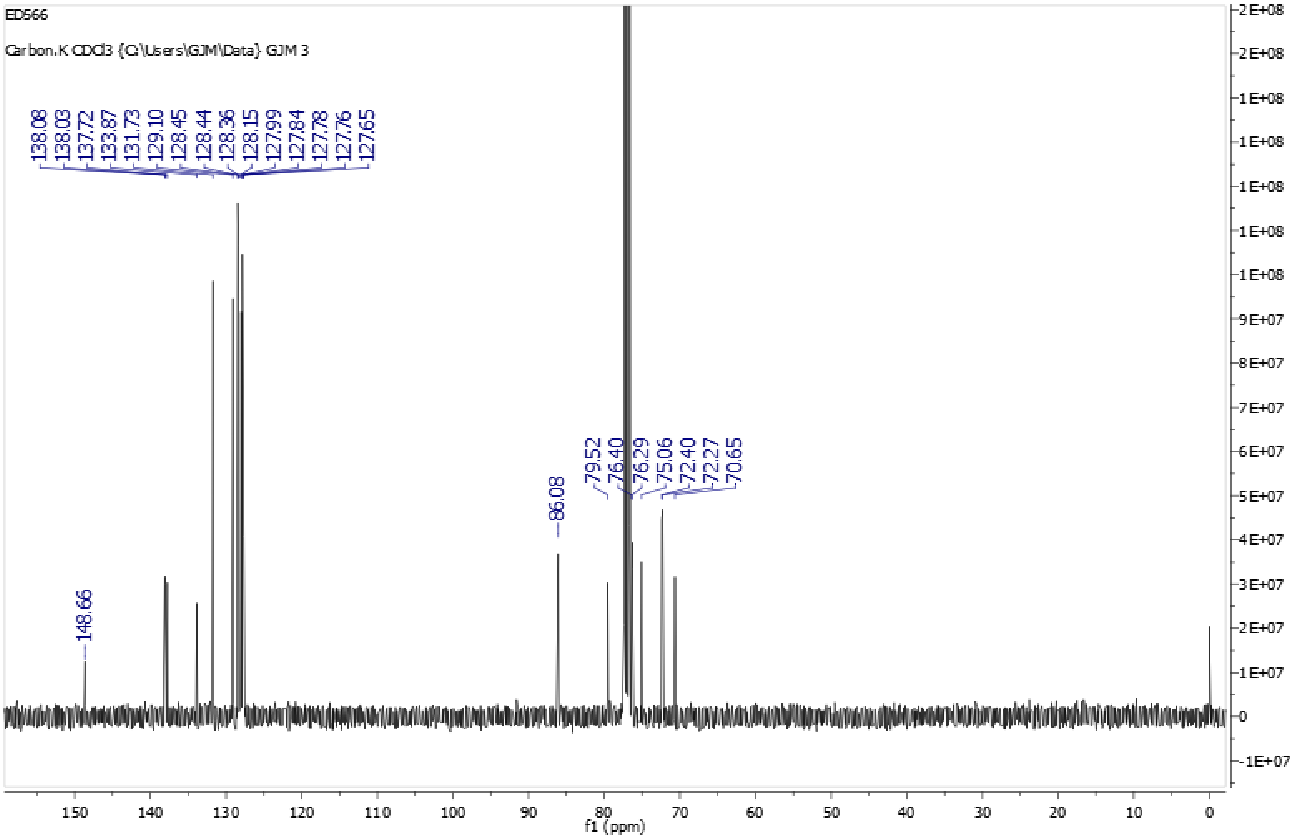

C-6 nitrile thioglycoside 16

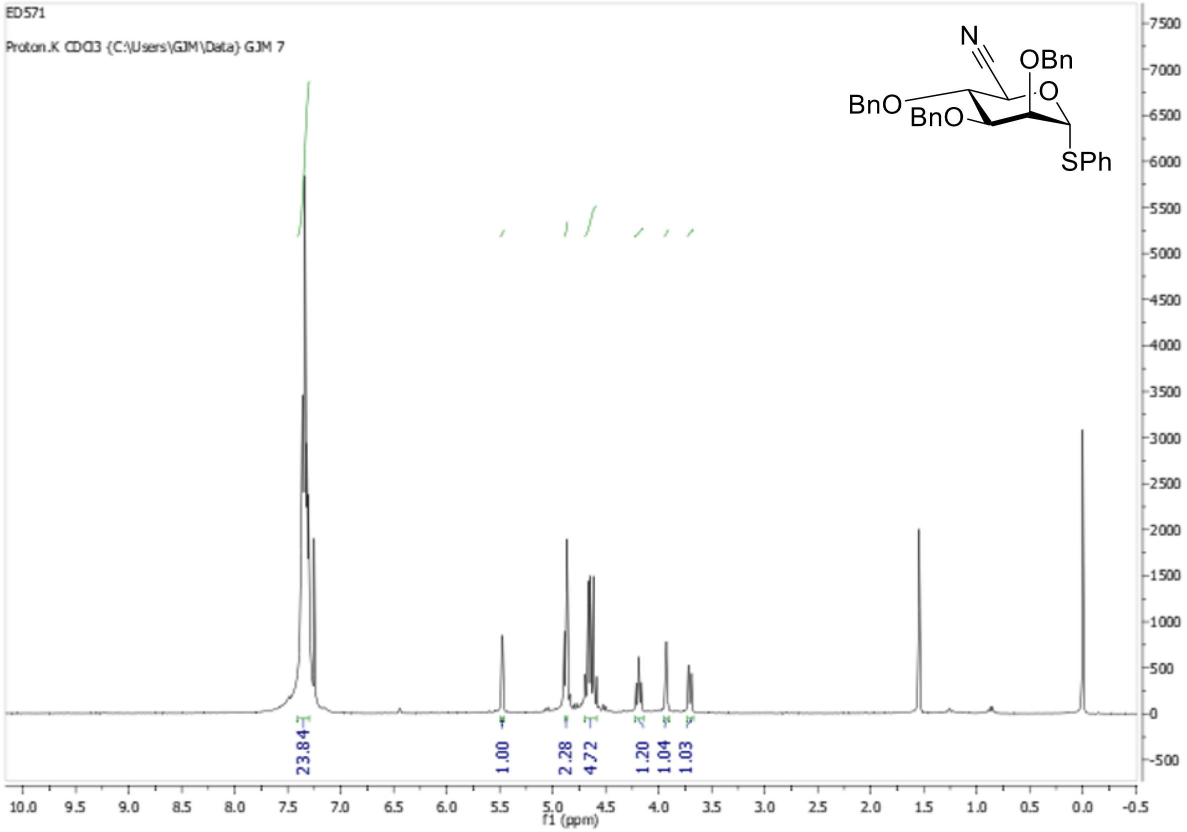

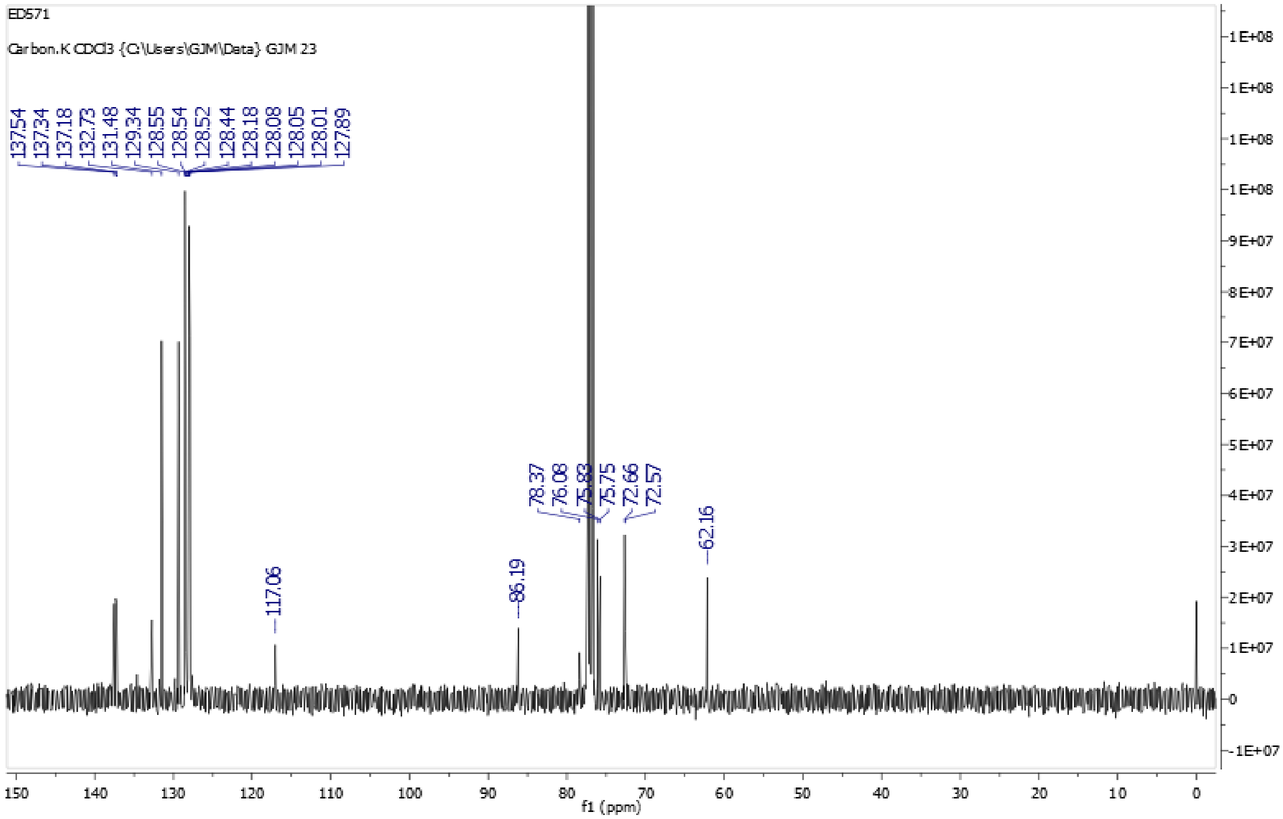

Compound 17

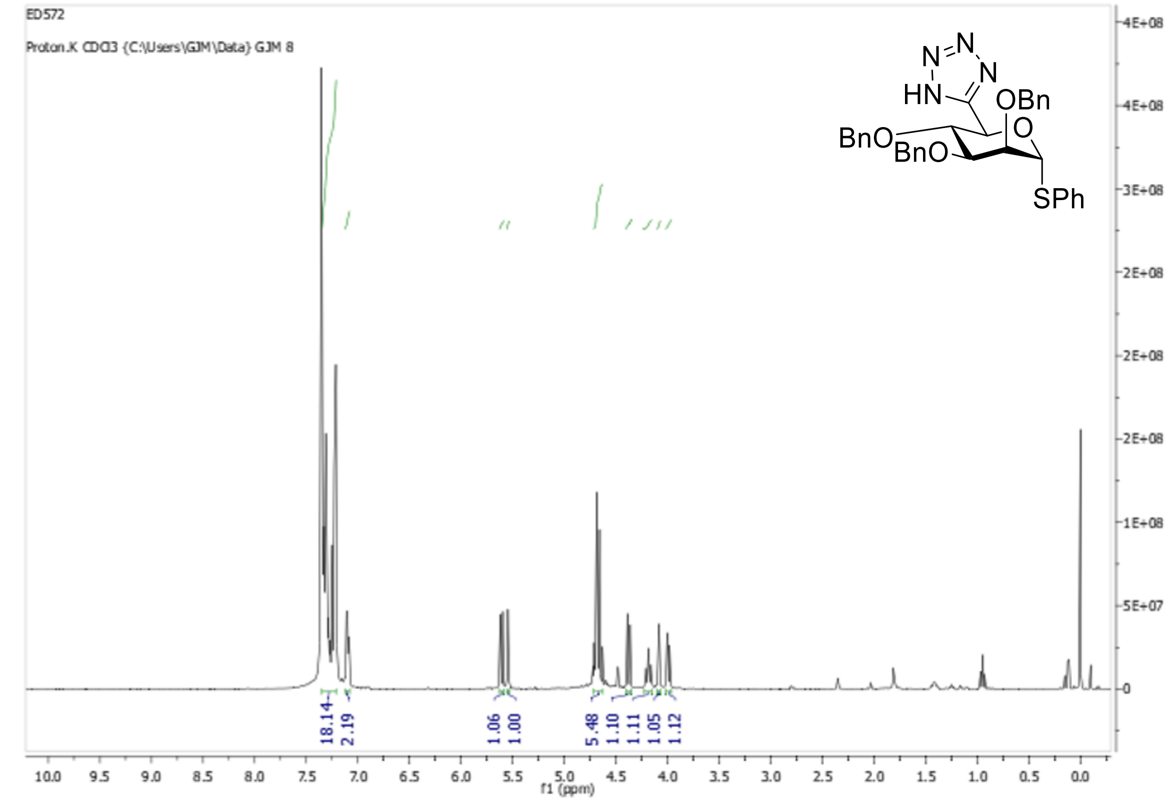

ED572
Proton.K CDCl3 {C:\Users\GJM\Data} GJM 8
N=N
N
HN
OBn
BnO
BnO
O
SPh
18.14
2.19
1.06
1.00
5.48
1.10
1.11
1.05
1.12
f1 (ppm)

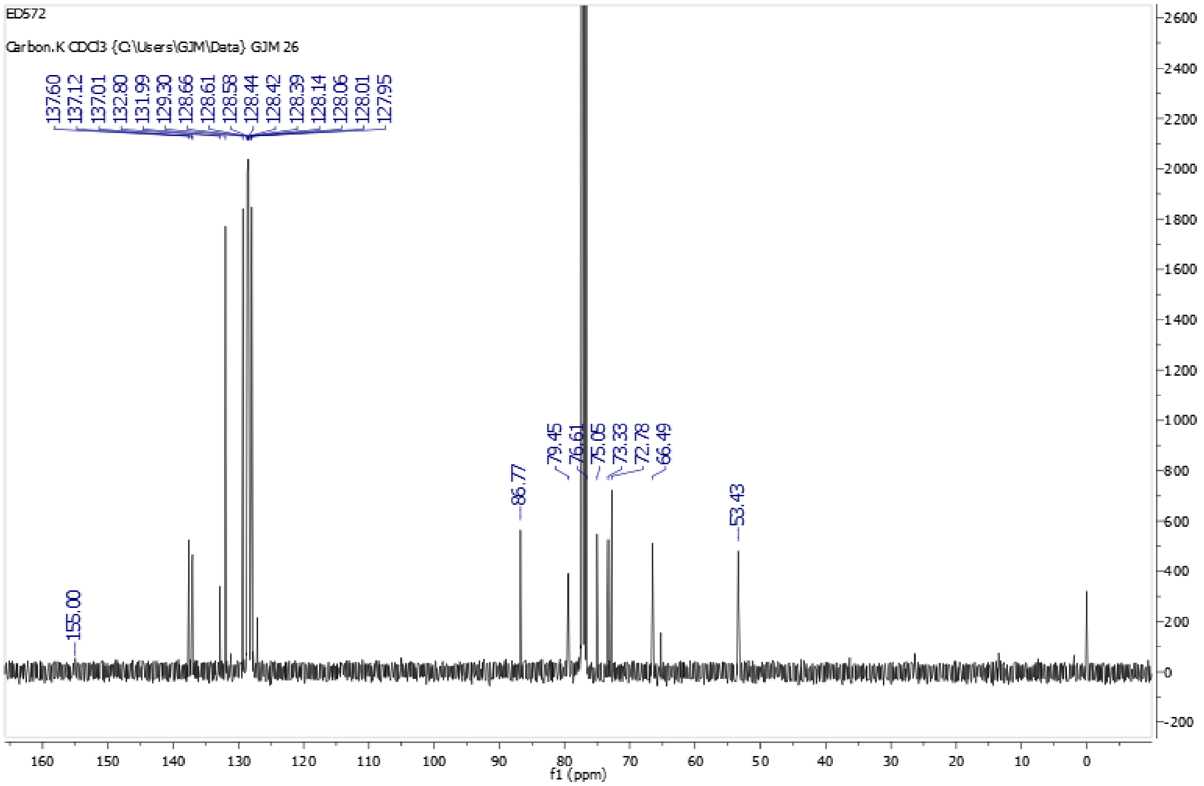

ED572
Carbon.K CDCl3 {C:\Users\GJM\Data} GJM 26
137.60
137.12
137.01
132.80
131.99
129.30
128.66
128.61
128.58
128.44
128.42
128.39
128.14
128.06
128.01
127.95
155.00
86.77
79.45
76.61
75.05
73.33
72.78
66.49
53.43
f1 (ppm)

Compound 18

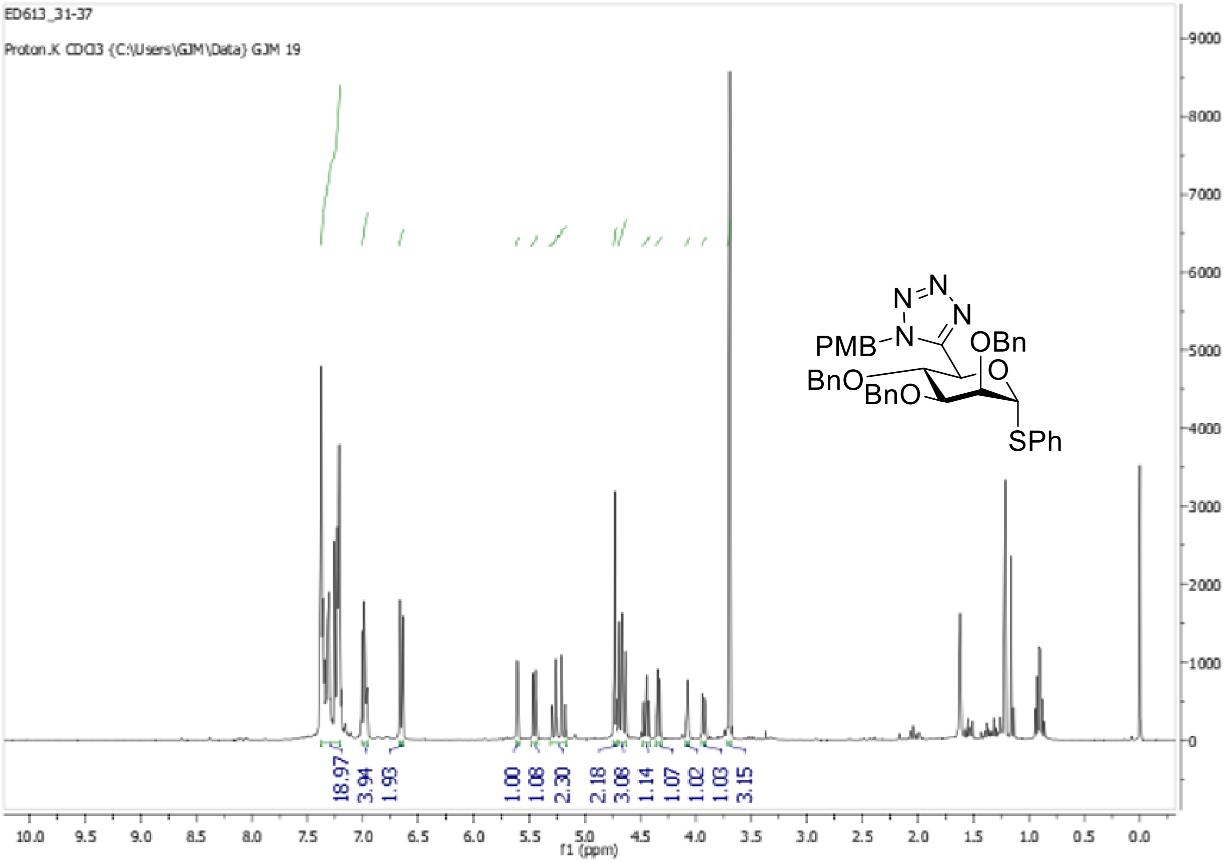

ED613_31-37
Proton.K CDCl3 {C:\Users\GJM\Data} GJM 19
18.97
3.94
1.93
1.00
1.08
2.30
2.18
3.08
1.14
1.07
1.02
1.03
3.15
f1 (ppm)
PMB
N
BnO
BnO
OBn
O
SPh

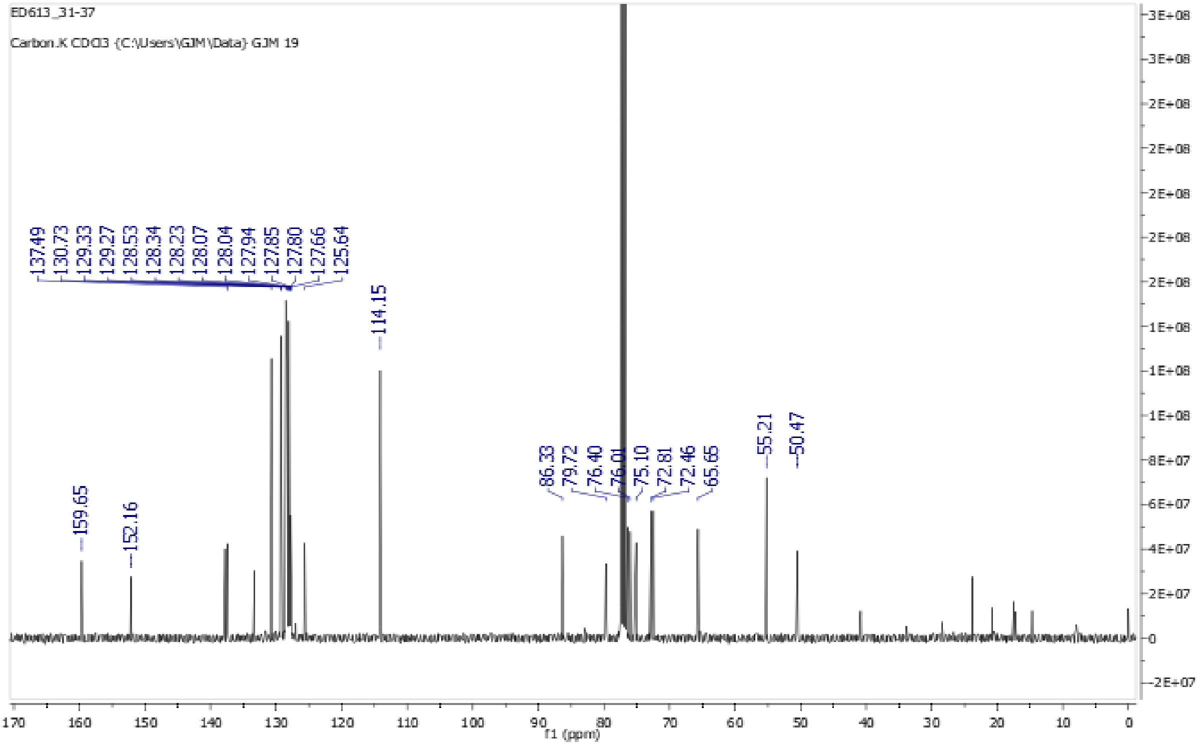

ED613_31-37
Carbon.K CDCl3 {C:\Users\GJM\Data} GJM 19
137.49
130.73
129.33
129.27
128.53
128.34
128.23
128.07
128.04
127.94
127.85
127.80
127.66
125.64
114.15
159.65
152.16
86.33
79.72
76.40
76.01
75.10
72.81
72.46
65.65
55.21
50.47
f1 (ppm)

Coupled HSQC of **N1-isomer**

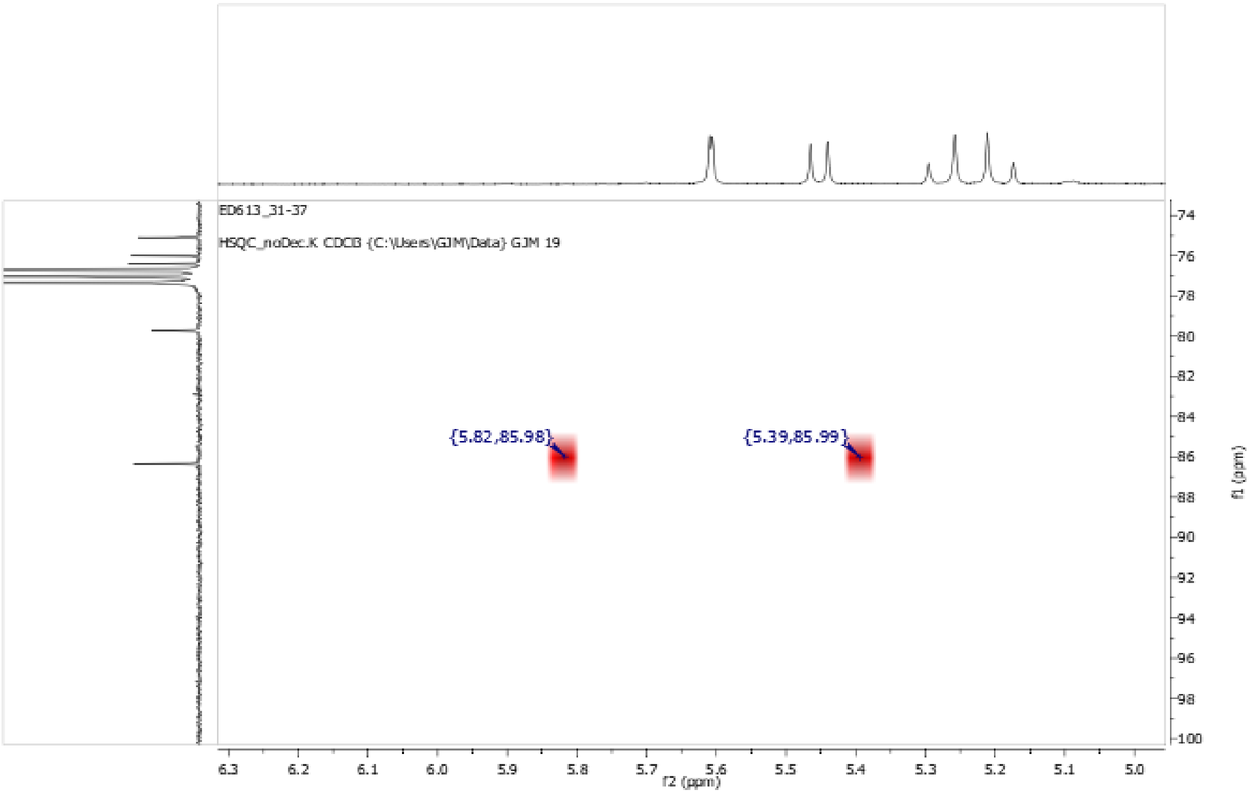

Coupled HSQC of **N2-isomer**

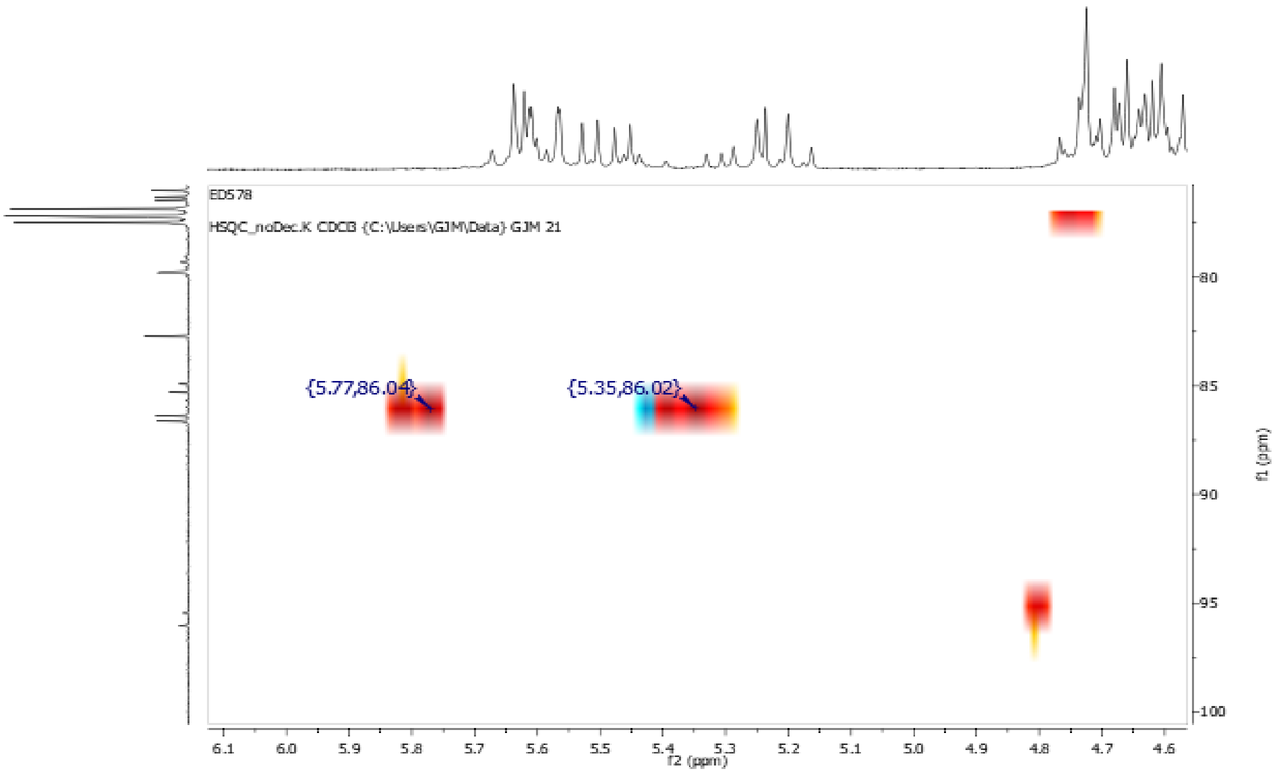

HMBC of both isomers

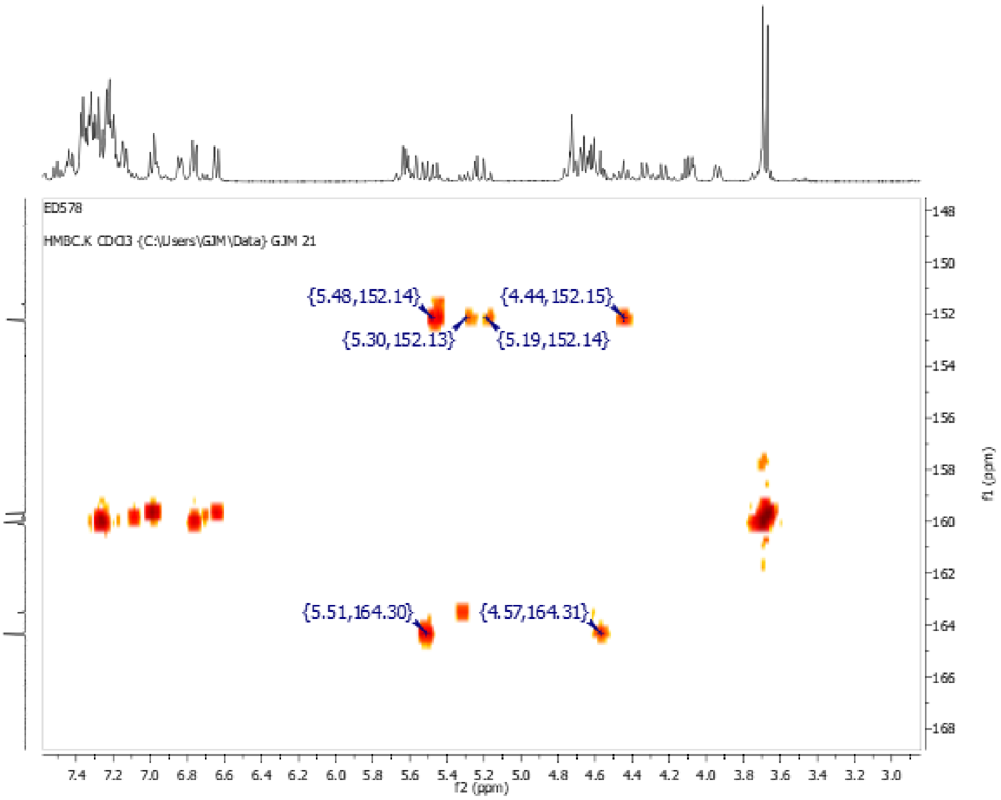

3-(Benzyloxycarbonylamino)propyl (2,3,4-tri-*O*-benzyl-6-*C*-(2-para-methoxybenzyl -tetrazolyl)-α/β-D-lyxopyranoside

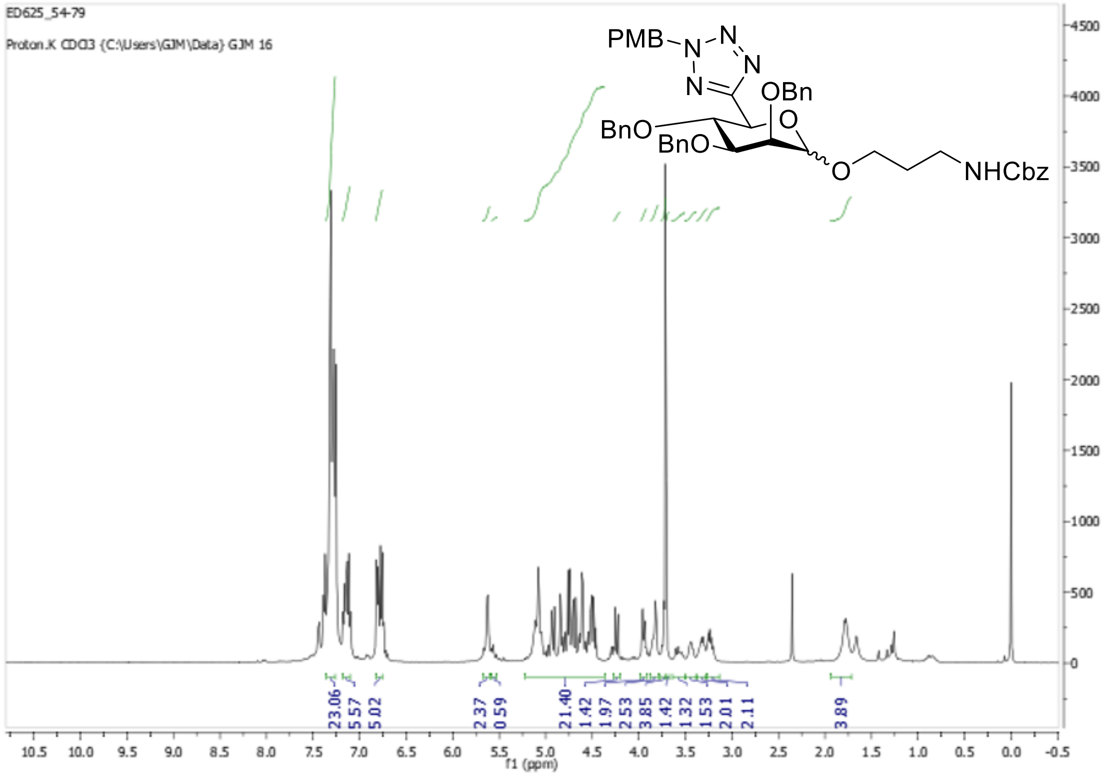

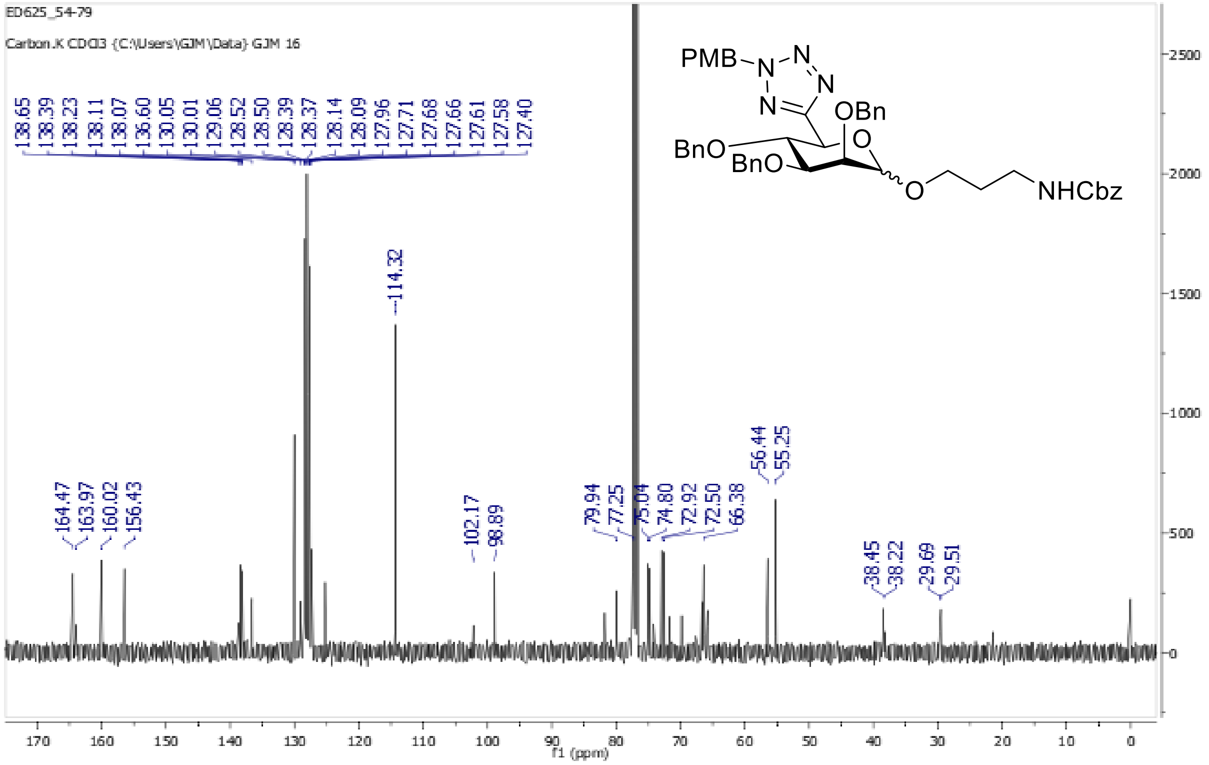

Coupled HSQC (showing only β-anomer $^{1}J_{C1\text{-}H1}$ coupling)

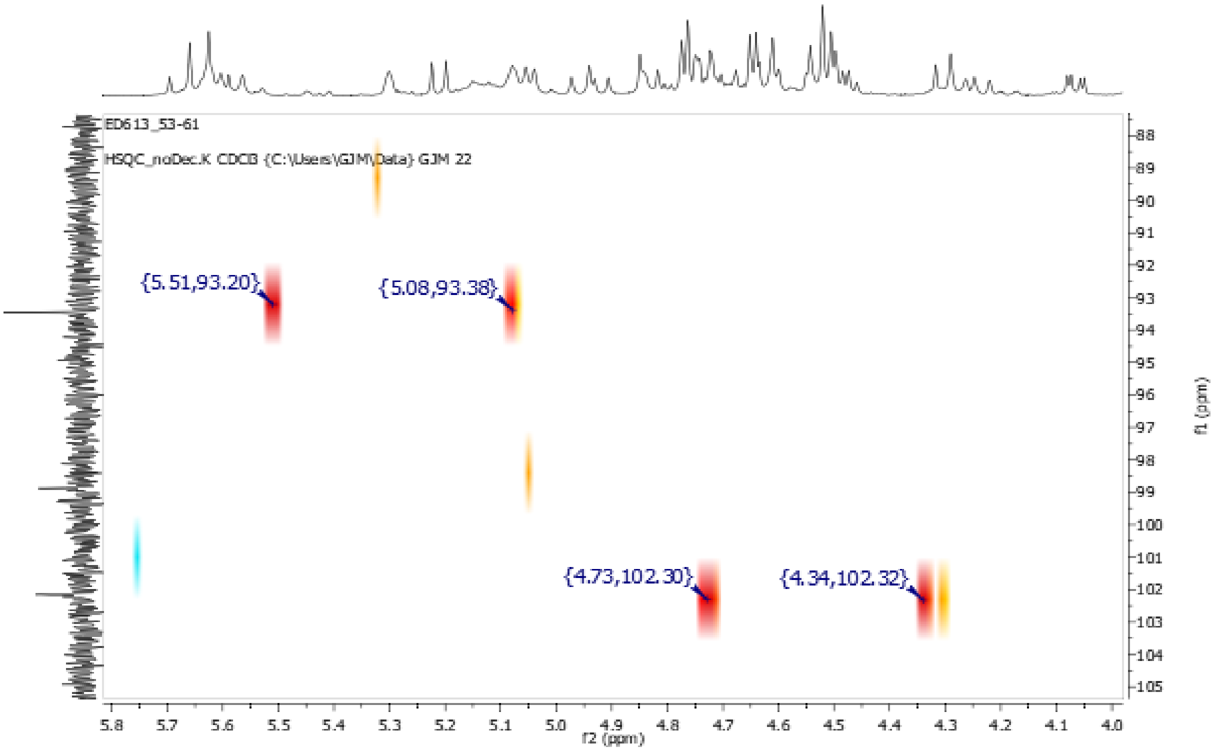

Compound 20

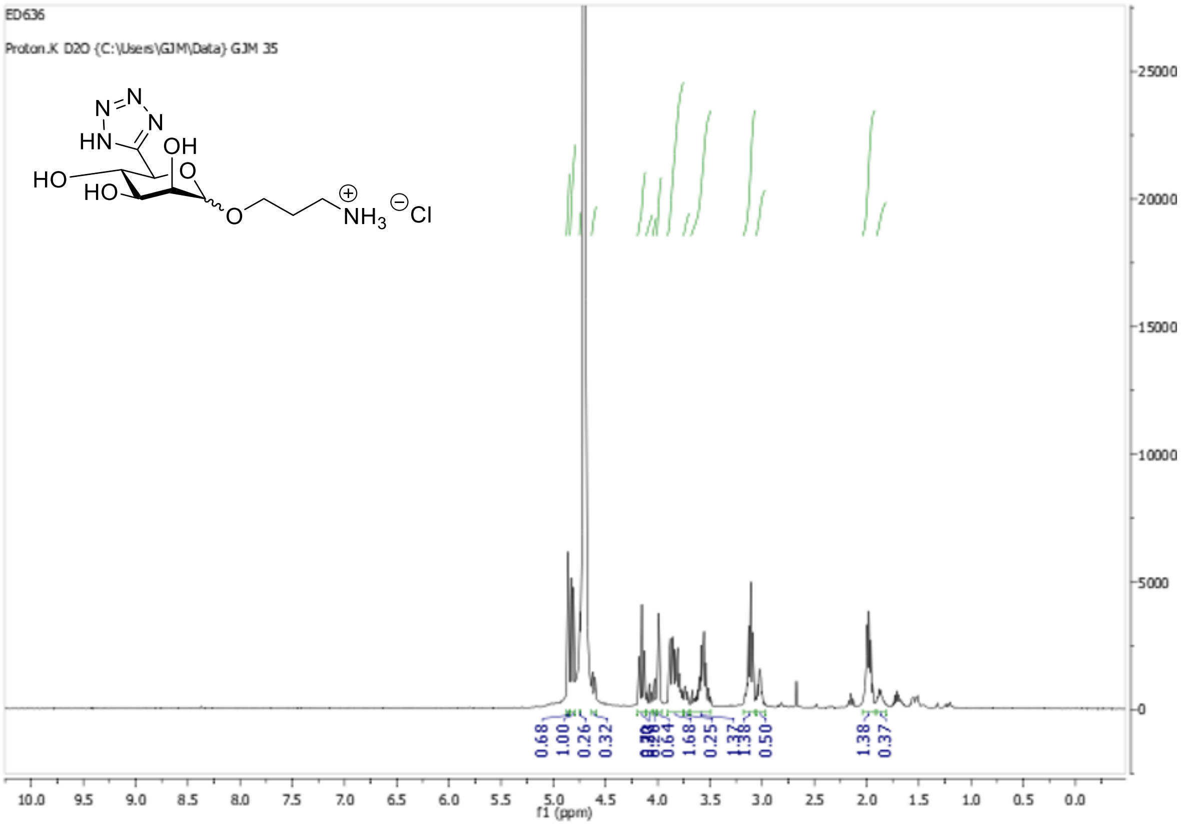

ED636
Proton.K D2O {C:\Users\GJM\Data} GJM 35
0.68
1.00
0.26
0.32
0.64
1.68
0.25
1.37
1.38
0.50
1.38
0.37
f1 (ppm)

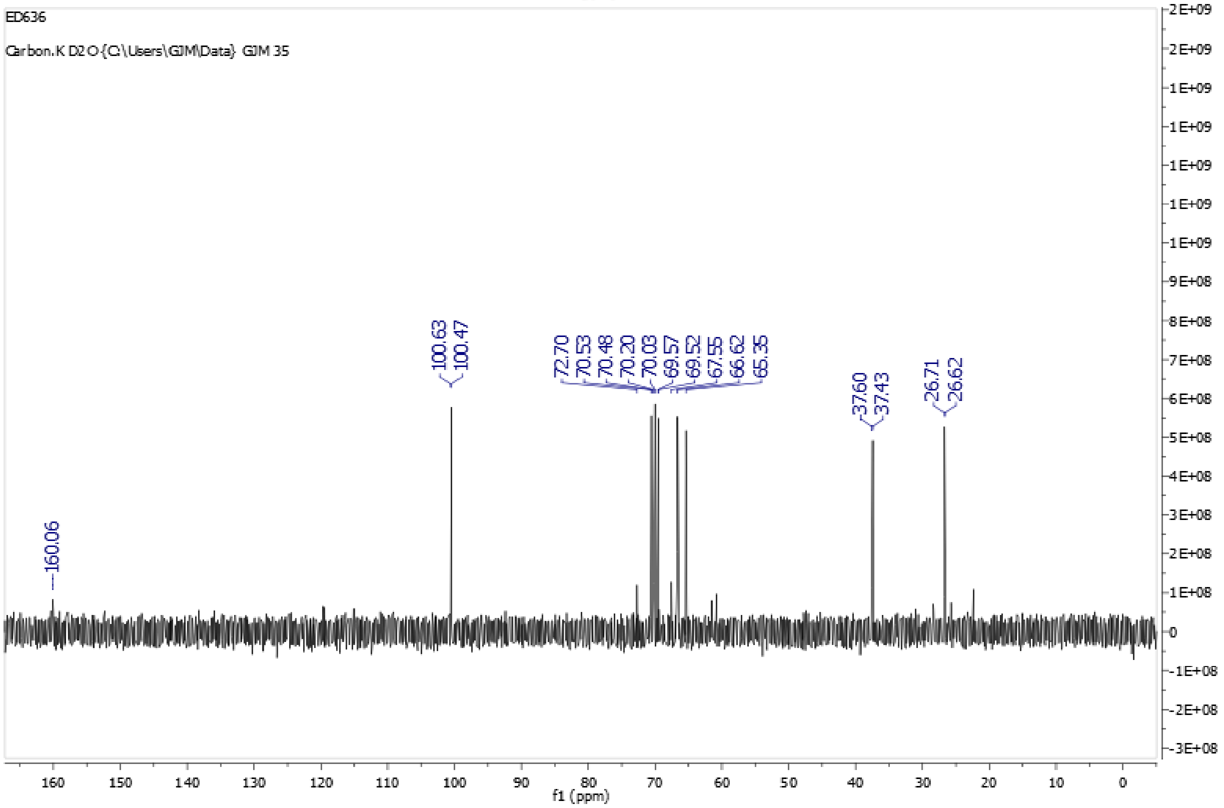

ED636
Carbon.K D2O {C:\Users\GJM\Data} GJM 35
160.06
100.63
100.47
72.70
70.53
70.48
70.20
70.03
69.57
69.52
67.55
66.62
65.35
37.60
37.43
26.71
26.62
f1 (ppm)

Coupled HSQC

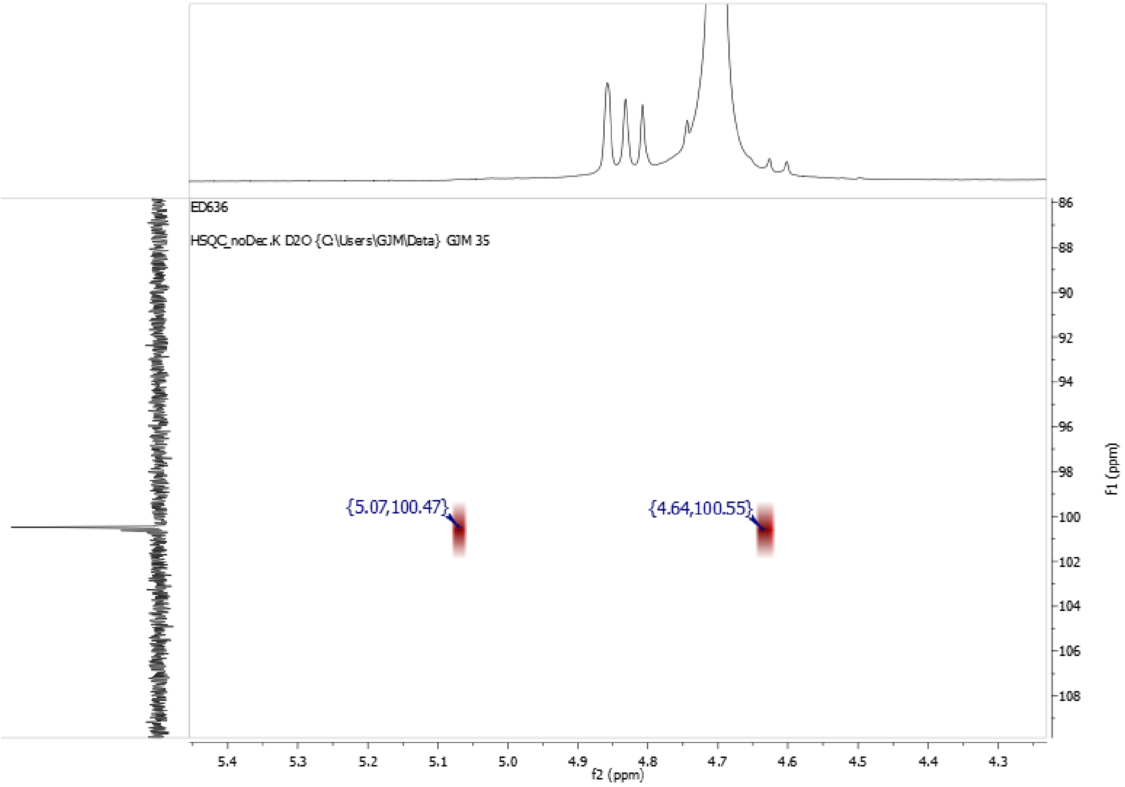

Fully protected C6-tetrazole 1-phosphates

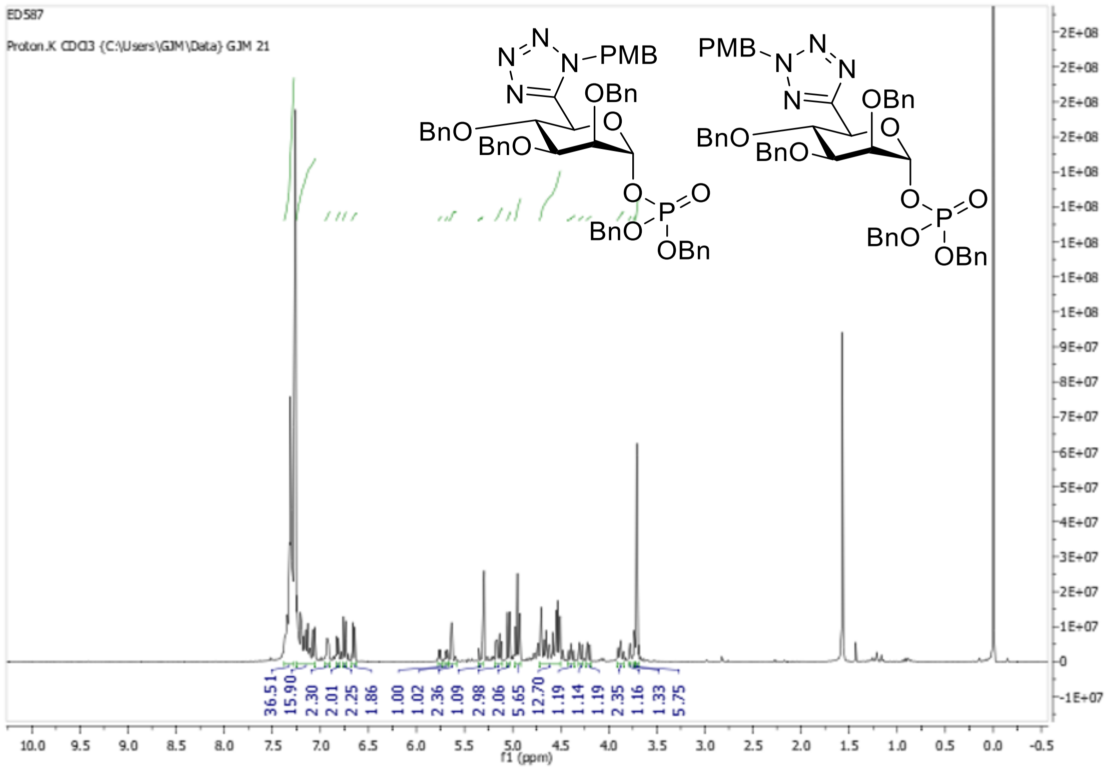

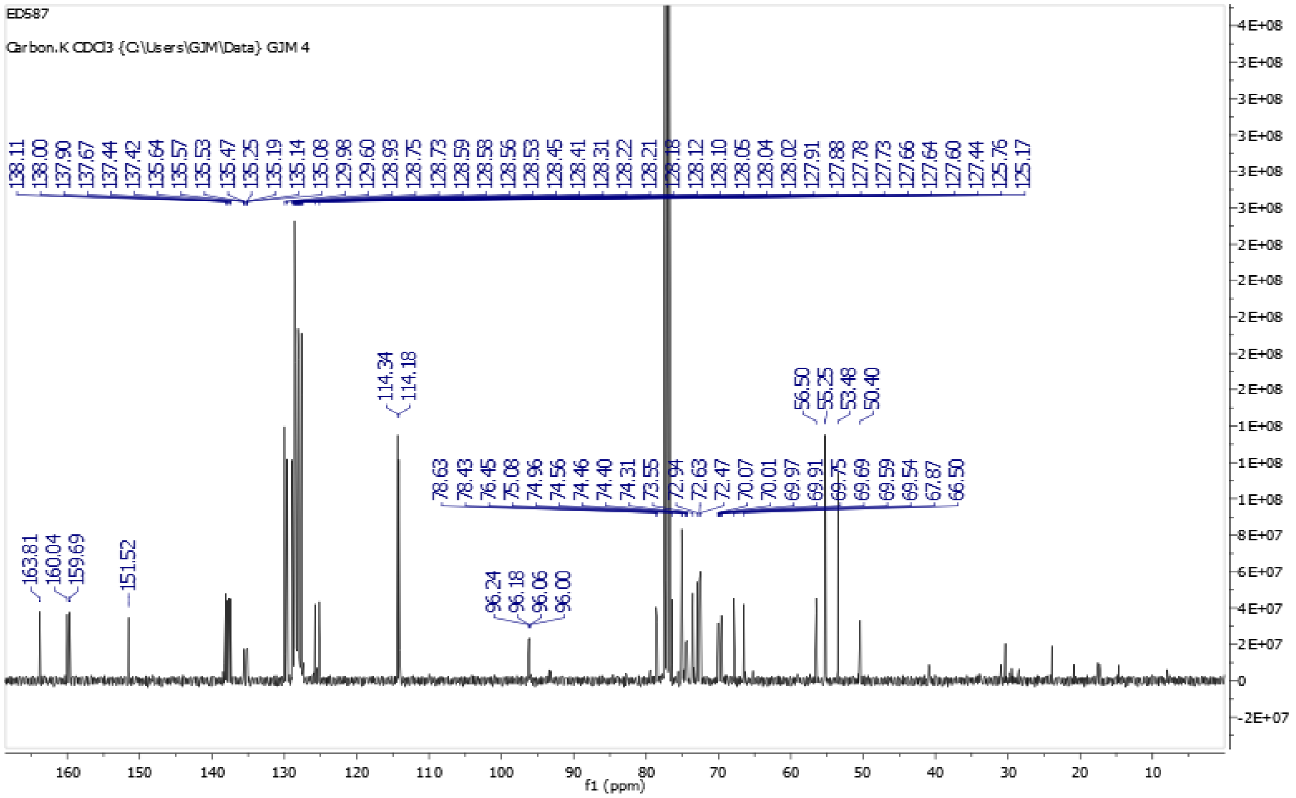

^{31}P NMR

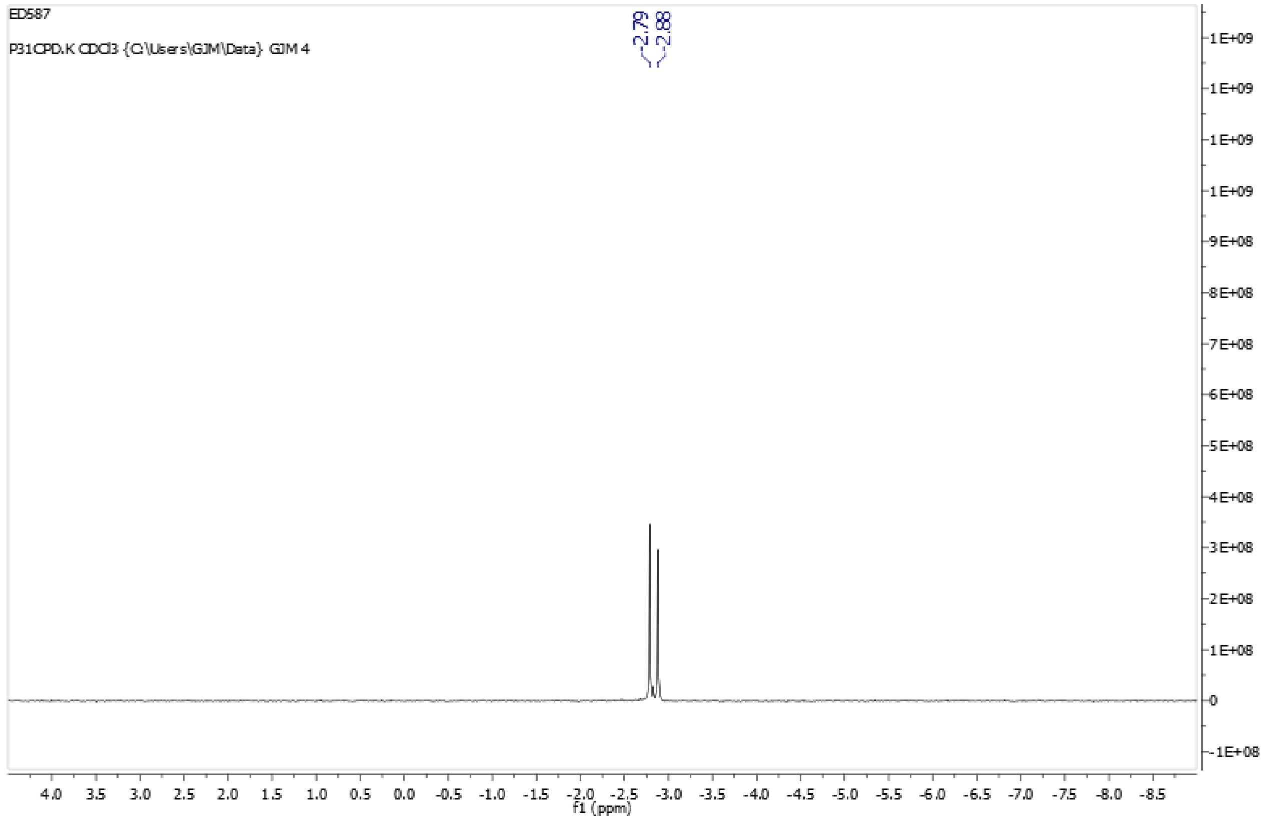

Compound 21

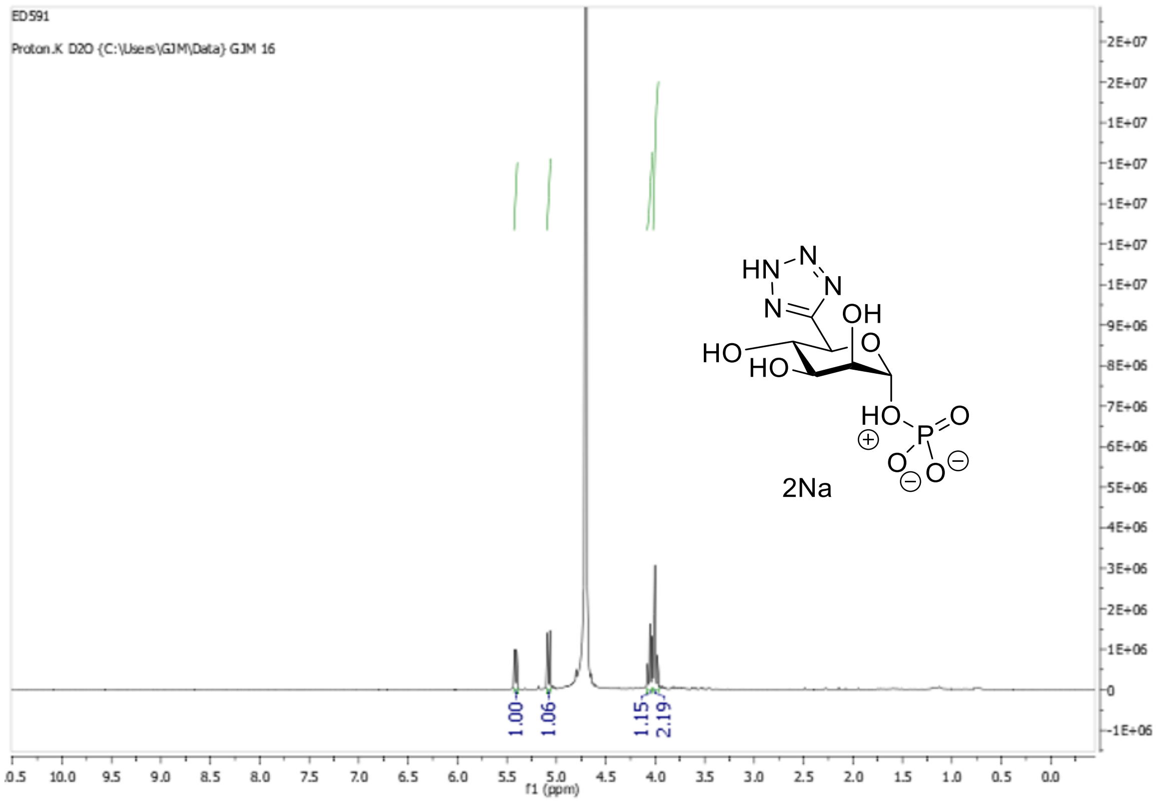
ED591
Proton.K D2O {C:\Users\GJM\Data} GJM 16
HN
N
N
N
OH
HO
O
HO
HO
P
O
O
O
2Na
1.00
1.06
1.15
2.19
f1 (ppm)

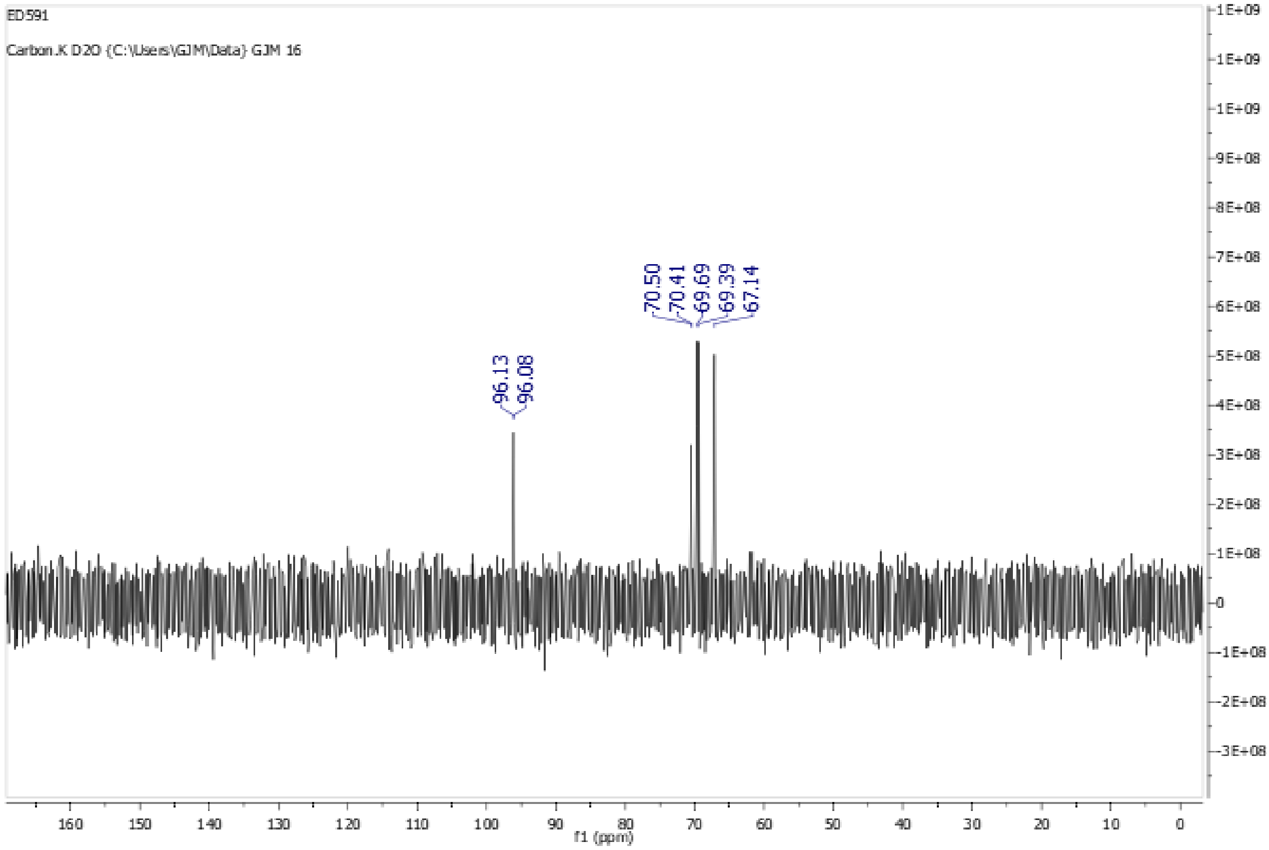
ED591
Carbon.K D2O {C:\Users\GJM\Data} GJM 16
96.13
96.08
70.50
70.41
69.69
69.39
67.14
f1 (ppm)

^{31}P NMR

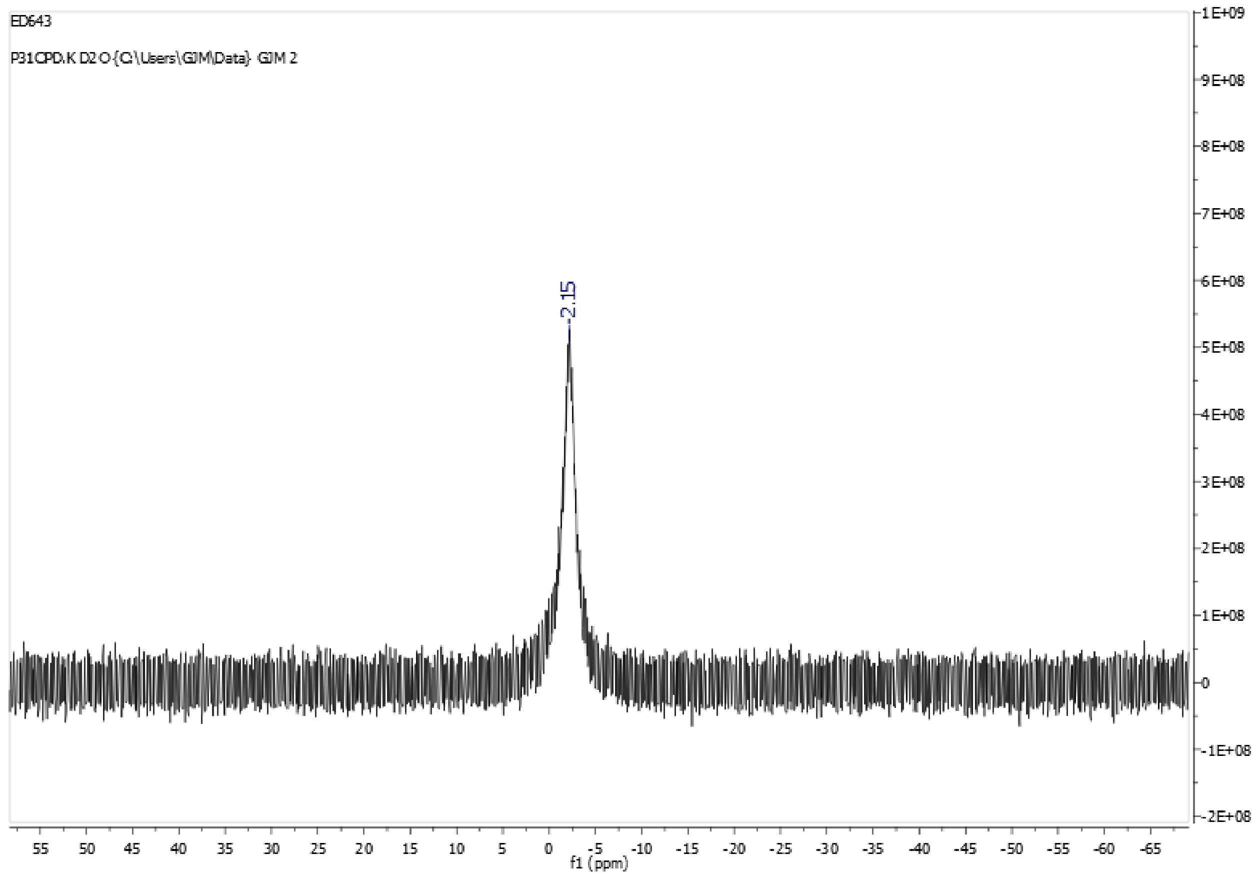

Supplement: File 1 — Detailed experimental protocols and characterisation data; spectral NMR data (1H, 13C, 31P and HSQC NMR for compounds 2–5, 7–14, 16–18, 20 and 21). [file Beilstein_J_Org_Chem-17-1527-s001.pdf]
